# Emerging 2D Nanomaterials‐Integrated Hydrogels: Advancements in Designing Theragenerative Materials for Bone Regeneration and Disease Therapy

**DOI:** 10.1002/advs.202403204

**Published:** 2024-06-14

**Authors:** Melanie Zorrón, Agustín López Cabrera, Riya Sharma, Janani Radhakrishnan, Samin Abbaszadeh, Mohammad‐Ali Shahbazi, Omid Aghababaei Tafreshi, Solmaz Karamikamkar, Hajar Maleki

**Affiliations:** ^1^ Institute of Inorganic Chemistry Department of Chemistry Faculty of Mathematics and Natural Sciences University of Cologne Greinstraße 6 50939 Cologne Germany; ^2^ Department of Biotechnology National Institute of Animal Biotechnology Hyderabad 500 049 India; ^3^ Department of Pharmacology and Toxicology School of Pharmacy Urmia University of Medical Sciences Urmia 571478334 Iran; ^4^ Department of Biomaterials and Biomedical Technology University Medical Center Groningen University of Groningen Antonius Deusinglaan 1 Groningen AV, 9713 The Netherlands; ^5^ Microcellular Plastics Manufacturing Laboratory Department of Mechanical and Industrial Engineering University of Toronto Toronto Ontario M5S 3G8 Canada; ^6^ Smart Polymers & Composites Lab Department of Mechanical and Industrial Engineering University of Toronto Toronto Ontario M5S 3G8 Canada; ^7^ Terasaki Institute for Biomedical Innovation 11570 W Olympic Boulevard Los Angeles CA 90024 USA; ^8^ Center for Molecular Medicine Cologne CMMC Research Center Robert‐Koch‐Str. 21 50931 Cologne Germany

**Keywords:** 2D nanomaterials, bone cancer therapy, bone regeneration, composites, hydrogel, theragenerative materials

## Abstract

This review highlights recent advancements in the synthesis, processing, properties, and applications of 2D‐material integrated hydrogels, with a focus on their performance in bone‐related applications. Various synthesis methods and types of 2D nanomaterials, including graphene, graphene oxide, transition metal dichalcogenides, black phosphorus, and MXene are discussed, along with strategies for their incorporation into hydrogel matrices. These composite hydrogels exhibit tunable mechanical properties, high surface area, strong near‐infrared (NIR) photon absorption and controlled release capabilities, making them suitable for a range of regeneration and therapeutic applications. In cancer therapy, 2D‐material‐based hydrogels show promise for photothermal and photodynamic therapies, and drug delivery (chemotherapy). The photothermal properties of these materials enable selective tumor ablation upon NIR irradiation, while their high drug‐loading capacity facilitates targeted and controlled release of chemotherapeutic agents. Additionally, 2D‐materials ‐infused hydrogels exhibit potent antibacterial activity, making them effective against multidrug‐resistant infections and disruption of biofilm generated on implant surface. Moreover, their synergistic therapy approach combines multiple treatment modalities such as photothermal, chemo, and immunotherapy to enhance therapeutic outcomes. In bio‐imaging, these materials serve as versatile contrast agents and imaging probes, enabling their real‐time monitoring during tumor imaging. Furthermore, in bone regeneration, most 2D‐materials incorporated hydrogels promote osteogenesis and tissue regeneration, offering potential solutions for bone defects repair. Overall, the integration of 2D materials into hydrogels presents a promising platform for developing multifunctional theragenerative biomaterials.

## Graphical abstract

2D nanomaterials, with their ultrathin structures and unique properties, have revolutionized hydrogel‐based therapies for bone regeneration and disease treatment. This review discusses advancements in integrating 2D materials like graphene and MXene into hydrogels, enhancing their mechanical properties, drug release capabilities, and therapeutic efficacy in bone repair, cancer treatments, and antibacterial applications.

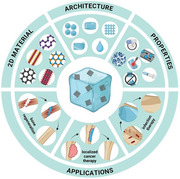

## 1 Introduction

The main functions of bone are structural support and movement, storage of energy and minerals, and the production of new blood cells in the bone marrow.^[^ ^1^ ^]^ Therefore, its well‐being is crucial for the good functioning of the body. Fortunately, bone possesses an intrinsic ability to regenerate, both during its natural development and remodeling processes, and when injured.^[^ ^2^ ^]^ In fact, bone defects can usually heal to such an extent that regenerated bone frequently eventually becomes indistinguishable from uninjured bone.^[^ ^2^ ^]^ However, when bone defects are too large, or other diseases are present in the body, natural bone healing can be insufficient.^[^ ^3^ ^]^ For instance, oral and maxillofacial surgeries sometimes cause significant bone defects, that cannot naturally be healed fully.^[^ ^3^ ^]^ In these cases, bone regeneration can be aided with tissue engineering approaches, which use biomedical implants to stimulate and guide the healing process.^[^ ^3^ ^]^

Another common problem affecting the health of bones are malignant tumors. Despite bone cancer not being among the most common types of cancer, metastasis to the bone is quite common: most patients with advanced prostate or breast cancer eventually develop bone metastases.^[^ ^1^, ^4^ ^]^ As a matter of fact, it is estimated that over 350,000 people die annually in the United States from cancer that has spread to the bone.^[^ ^4^ ^]^ Moreover, once metastasis to the bone develops, the likelihood of the cancer being cured falls significantly.^[^ ^1^ ^]^ Much research is being conducted in this field, mainly on photothermal therapy for the removal of residual cancer cells and tissue engineering for aiding bone regeneration following surgery.^[^ ^5^ ^]^

Furthermore, severe fractures, malignant tumors, and bone surgery (particularly the implantation of medical devices) are predisposing factors developing bone infections.^[^ ^6^ ^]^ One such infectious condition is osteomyelitis, an infection with inflammation of the bone and bone marrow which, if improperly treated, can lead to the development of a chronic infection.^[^ ^6^, ^7^ ^]^ This kind of infection tends to be resistant and recurrent, has high morbidity, and requires expensive medical care.^[^ ^8^ ^]^ For these reasons, infectious conditions of the bone are another focal point of the research being conducted by the scientific community into the treatment of bone diseases.^[^ ^5^, ^9^ ^]^

2D materials are nanomaterials consisting of nano‐scaled sheets. These nanosheets have thicknesses ranging from several angstroms to a few nanometers, and lateral lengths and widths that can reach the micrometric scale.^[^ ^10^ ^]^ Their 2D provides them with unique properties that differ from those of analogous 3D materials, such as large specific surface areas, excellent loading capacities, high thermal conductivities, and special optical properties, like broad light absorption and good photothermal conversion.^[^ ^10b^ ^]^ Additionally, majority of 2D materials are biocompatible and biodegradable, which extends their application range to the biomedical field.^[^ ^11^ ^]^ In particular, 2D materials such as graphene and its derivatives, MXenes, black phosphorus (BP), hexagonal boron nitride (hBN), and transition metal dichalcogenides (TMDs), have been widely studied for the treatment of the aforementioned bone diseases.^[^ ^5^, ^12^ ^]^ Most of these 2D materials have shown good cell adhesion abilities and release of degradation products which can aid the biomineralization process in bone.^[^ ^13^ ^]^ These properties make them good candidates for bone tissue engineering applications. Additionally, their strong near‐infrared (NIR) light absorption capabilities and photothermal conversion and drug loading capacities are promising for bone cancer therapy and bone infections.^[^ ^12^, ^14^ ^]^

Despite their numerous advantages, 2D materials have significant flaws which limit their applicability. Due to their small particle size, they exhibit an initial burst release of component species or loaded drugs, which leads to high concentrations that can cause toxic effects.^[^ ^15^ ^]^ Furthermore, they frequently show fast dispersion away from the target site and undergo rapid elimination from the body, which limits their therapeutic outcome.^[^ ^15b^ ^]^ Therefore, a transition from these 2D materials to 3D materials is necessary to minimize these shortcomings. Hydrogels, which consist of 3D networks containing very high amounts of water, have been studied widely for this purpose due to their particularly advantageous properties.^[^ ^5^, ^12^ ^]^ They usually possess excellent biocompatibility and biodegradability, good drug loading and sustained release capabilities, are easy to functionalize, and closely resemble the extracellular matrix.^[^ ^15^, ^16^ ^]^ Incorporating 2D materials into hydrogels significantly addresses the limitations of hydrogels for biomedical applications. These limitations often include weak mechanical properties, limited responsiveness to stimuli, and a lack of specific biomedical functionalities, such as inadequate activity against tumors or infections.^[^ ^15^, ^17^ ^]^ However, the inclusion of 2D materials substantially improves these aspects. Consequently, a synergistic system is formed, in which the hydrogel serves as a carrier, enhancing the retention and stability of the 2D material, while the 2D material itself contributes the primary functionality and enhances the mechanical properties of the hydrogel system.^[^ ^15b^ ^]^

**Scheme 1:**
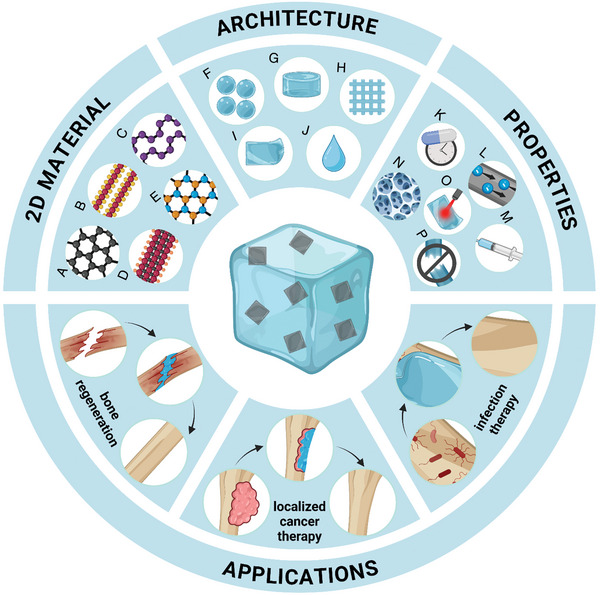
Schematic representation of the main aspects of current research on 2D materials‐based hydrogels with applications for the treatment of bone diseases and defects regeneration. The most relevant 2D materials, architectures and properties are highlighted: A) graphene and its derivatives, B) TMDs, C) BP, D) MXenes, and E) hBN; F) microgels, G) bulk, H) 3D‐printed and other complex architectures, I) coatings and J) in situ‐gelling hydrogels; K) controlled and sustained drug release, L) electrical conductivity, M) injectability, N) porosity, O) photothermal activity and P) mechanical strength.

Some reviews have been published, which focus on the biomedical applications of hydrogels incorporating specific 2D materials, highlighting their properties. Additionally, the biomedical applications of nanocomposite hydrogels in general,^[^ ^15b^ ^]^ and the applications of 2D materials for bone disease therapy have also been reviewed.^[^ ^18^ ^]^ However, to date there is no comprehensive review explicitly addressing all major groups of 2D materials and their applications as part of hydrogels for bone defect regeneration and therapy of bone diseases. Herein, we review the main groups of 2D materials‐based hydrogels, focusing on the current state of the art regarding their fabrication, properties and applications for bone regeneration, the treatment of bone infections and bone cancer, as well as theragenerative and combined treatment approaches (*cf*. **Scheme** **1**). Finally, comprehensive insights on the future research direction in the field of 2D materials‐based hydrogels for the aforementioned application areas are provided.

## 2 2D Materials: Synthesis, Properties, and Cytotoxicity

### 2.1 Graphene and its Derivatives

**Figure 1:**
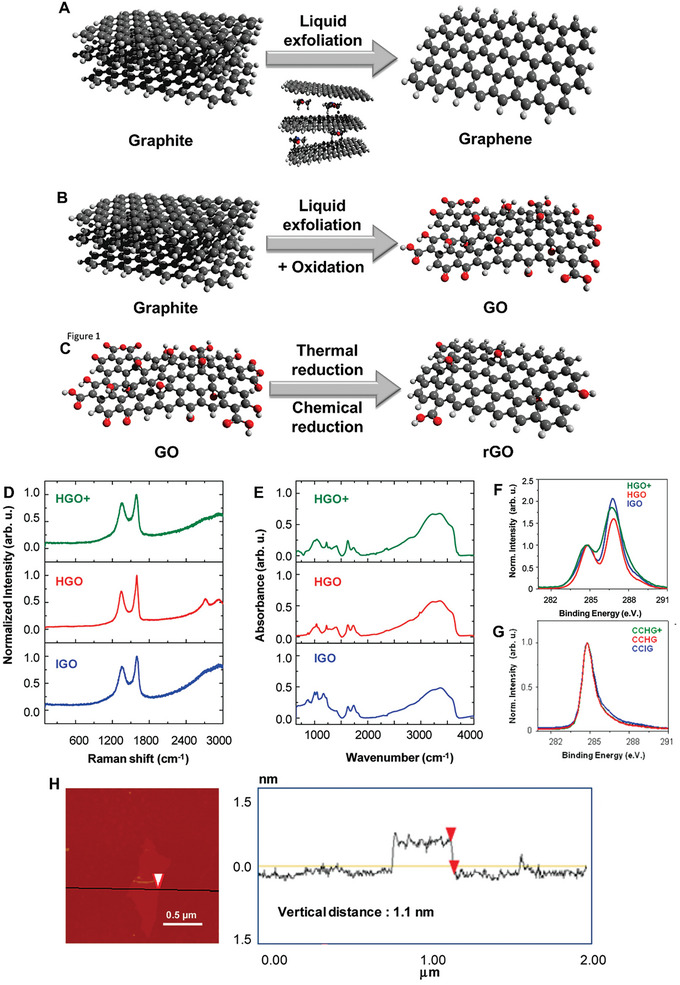
Schematic representation of the synthesis processes of A) graphene, B) graphene oxide, and C) reduced graphene oxide. Color code: black = C, light gray = H, red = O. D) Raman and E) infrared spectra of GO synthesized using Hummer's method (HGO), modified Hummer's method (HGO+) and improved Hummer's method (IGO). C1s XPS spectra of GO synthesized using different methods both F) before and G) after chemical reduction with hydrazine. H) AFM image of a GO flake synthesized using the improved Hummer's method. Height profile along the black path is on the right. Figures (D,H) Reproduced with permission.^[^ ^27b^ ^]^ Copyright 2010, American Chemical Society.

The first 2D material, graphene, was discovered in 2004 by Andre Geim and Konstantin Novoselov.^[^ ^19^ ^]^ Graphene is, for this reason, the most extensively studied 2D material. It consists of single sheets of sp^2^‐hybridized carbon atoms in a hexagonal honeycomb‐like structure (**Figure** **1**A), and its thermal, mechanical, and electrical properties are superior to those of graphite, its parent 3D material.^[^ ^20^ ^]^ A single layer of graphene has a thermal conductivity of ≈4500–5200 W Km^−1^ and an electrical conductivity of 10^4^ S cm^−1^, making it a promising material for tissue engineering applications.^[^ ^21^ ^]^ Specifically, the high electrical conductivity and surface area of graphene can offer a greater osteogenic activity to bone repair materials.^[^ ^22^ ^]^ Additionally, the full spectrum absorption properties of graphene and its derivatives make them excellent candidates for photothermal therapy applications.^[^ ^23^ ^]^

Even though high‐purity graphene can be synthesized using bottom‐up methods,^[^ ^24^ ^]^ these are not usually used for biomedical applications due to their poor scalability. Instead, graphene is commonly synthesized by top‐down methods, for instance by liquid phase exfoliation. During this process, graphite is dispersed in a solvent (such as *N*,*N*‐dimethyl‐formamide, *N*‐methyl‐2‐pyrrolidone, or dichlorobenzene) in order to weaken the interaction between its sheets, forming single‐ and multi‐layered graphene sheets. This process is accompanied with ultrasonication or shearing to produce further exfoliation.^[^ ^24^, ^25^ ^]^

However, the application of synthesized graphene is often limited by its poor dispersibility in water.^[^ ^26^ ^]^ The most common way to overcome this problem is by treating graphite with strong oxidants, which add oxygen‐containing functional groups such as epoxy, hydroxyl, and carboxylate groups (Figure 1G), yielding graphene oxide (GO) (Figure 1B). The presence of these oxygenated groups in GO allows for easy dispersion in a wide range of polar solvents, including water.^[^ ^26^ ^]^ The most common synthesis routes of GOs are the so‐called Hummers’ method and its variants.^[^ ^27^ ^]^ For example, in the improved Hummers’ method^[^ ^27b^ ^]^ a mixture of concentrated H_2_SO_4_ and H_3_PO_4_ (9:1 volume ratio) is mixed with a solid mixture of graphite flakes and KMnO_4_ (1:6 mass ratio) and stirred at 50 °C for 12 h. The reaction mixture is then cooled down to room temperature, poured onto distilled water ice with 30% H_2_O_2_, and filtrated. Finally, the filtrate is centrifugated, and the remaining solid material is washed with water, 30% HCl and ethanol.

This oxidation process, however, has some disadvantages: the presence of the oxygenated groups often modifies the electrical properties of graphene, making the synthesized GOs electrically insulating – in comparison with graphene, the electrical conductivity of GOs is usually ≈10000 times smaller, in the order of 10^−1^ S cm^−1^.^[^ ^21^ ^]^ The percentage of oxygen in GO also affects the mechanical and thermal properties. For instance, an oxygen content of only 5% causes a reduction of ≈90% in thermal conductivity with respect to graphene.^[^ ^28^ ^]^ A subsequent chemical reduction, either by reducing agents (L‐ascorbic acid,^[^ ^29^ ^]^ sodium borohydride,^[^ ^30^ ^]^ or hydrazine^[^ ^29^, ^31^ ^]^) or thermal treatment (300 °C for 5 min,^[^ ^32^ ^]^ 350 °C for 30 min,^[^ ^33^ ^]^ or using microwaves^[^ ^34^ ^]^), can be performed in order to recover, to some extent, the pristine graphene's properties.^[^ ^35^ ^]^ This produces a material called reduced GO (rGO) (Figure 1C).

It has been demonstrated in several studies that graphene and its derivatives have toxicity toward both eukaryotic and prokaryotic cells. A summary of the most important findings is presented in **Table** **1**. Gurunathan et al.^[^ ^36^ ^]^ have already discussed the toxicity aspects of graphene and its derivatives in their comprehensive review. However, the toxicity of graphene is strongly correlated with its concentration in cell culture media, and the type of functional groups on the surface. For example, Sasidharan et al. showed that graphene accumulates in the cell membrane of Vero cells, causing high oxidative stress that eventually leads to apoptosis, whereas GO with carboxylic groups on the surface is internalized into the cell without causing cell toxicity.^[^ ^37^ ^]^

**Table 1:** 2D materials’ toxicity to different prokaryotic and eukaryotic cell lines.

2D material	Cell line	Incubation time	Approx. cell viability [%]	References
GO	*E. coli*	Cells spread on surface of sample, 60 min	40	[161]
rGO			15	
GO	*S. aureous*		30	
rGO			<10	
GO	*P. aeruginosa*	75 µg mL^−1^, 4 h	85	[162]
rGO		75 µg mL^−1^, 4 h	85	
Graphene	*E. coli*	1.0 mg mL^−1^, 3 h	30	[163]
	*B. subtillis*		25	
GO	*E. coli* DH5α	85 µg mL^−1^, 2 h	1.5	[164]
rGO			<10	
rGO	A594	20 µg mL^−1^, 2 h	47	
		85 µg mL^−1^, 2 h	15	
Graphene	Vero	300 µg mL^−1^, 24 h	40	[37]
GO			100	
Graphene	Neuronal PC12	1.0 µg mL^−1^, 24 h	60	[165]
GO	U87	100 µg mL^−1^, 3 h	72	[166]
rGO			36	
GO	U118		78	
rGO			49	
Graphene	U87	200 µg mL^−1^, 24 h	46	[167]
	HS‐5		40	
GO	KMS‐12‐BM	100 µg mL^−1^, 24 h	20	[168]
	H929		20	
	U266		20	
	MM1S		15	
	DOHH‐2		25	
	KARPAS299		30	
Graphene	MDA‐MB‐231	80 µg mL^−1^, 24 h	75	[169]
	PC3		75	
	B16F10		85	
GO	MDA‐MB‐231		95	
	PC3		85	
	B16F10		100	
Graphene (30 nm diameter)	HEK‐293T	100 µg mL^−1^, 24 h	15	[170]
Graphene (300 nm diameter)			75	
GO (30 nm diameter)			65	
GO (300 nm diameter)			85	
BNNS (2.0 µm diameter)	SaOS2	1 mg mL^−1^, 7 days	65	[50]
hBNNS (100 µm diameter)			15	
hBNNS‐OH	L929	3–200 µg mL^−1^, 24 h	100	[51]
hBNNS film	*E. coli* K‐261	Cell dispersion over film, 8 h	10	[171]
		Cell dispersion over film, 24 h	1	
hBNNS	HeLa	2–200 µg mL^−1^, 24 h	100	[172]
hBNNS	CRL 2120	25–100 µg mL^−1^, 24 h	100	[173]
		400 µg mL^−1^, 24 h	75	
		25–50 µg mL^−1^, 48 h	100	
		400 µg mL^−1^, 48 h	65	
	MDCK	25–100 µg mL^−1^, 24 h	100	
		400 µg mL^−1^, 24 h	80	
		25–100 µg mL^−1^, 48 h	100	
		400 µg mL^−1^, 48 h	70	
hBNNS	mHippo E‐14	88 µg mL^−1^, 24 h	85	[174]
		440 µg mL^−1^, 24 h	45	
hBNNS	HEK‐293T	20 µg mL^−1^, 48 h	100	[175]
		100 µg mL^−1^, 48 h	90	
	CHO	20 µg mL^−1^, 48 h	95	
		100 µg mL^−1^, 48 h	95	
hBNNS	NTERA‐2	0.19–0.78 µg mL^−1^, 24 h	100	[176]
		25 µg mL^−1^, 24 h	75	
hBNNS	HDF	10 µg mL^−1^, 24 h	100	[177]
		200 µg mL^−1^, 24 h	80	
		500 µg mL^−1^, 24 h	65	
	HUVEC	10 µg mL^−1^, 24 h	130	
		200 µg mL^−1^, 24 h	110	
		500 µg mL^−1^, 24 h	40	
BPNS (0.5 to 5 µm diameter)	*E. coli*	Cell dispersion over film, 2 h	2	[178]
	*P. aeruginosa*		4	
	MRSA		2	
	*S. typhimurium*		9	
	*B. cereus*		17	
	*C. albicans*		0	
	*C. auris*		15	
	*C. neoformans (Sensitive)*		10	
	*C. neoformans (F^R^)*		1	
	*C. neoformans (A^R^)*		2	
	L929	Cell dispersion over film, 48 h	105	
	BJ‐5TA		100	
BP quantum dots (5.3 nm diameter)	A549	20 µg mL^−1^, 24 h	70	[179]
	Beas‐2B		55	
BPNS	MDA‐MB‐231	0.2 µg mL^−1^, 4 h	100	[180]
		0.2 µg mL^−1^, 4 h, 10 min irradiation (660 nm, 0.5 W cm^−2^)	40	
BP quantum dots (2.1 nm diameter)	HeLa	500 µg mL^−1^, 48 h	92	[181]
		100 mg mL^−1^, 12 h, 10 min irradiation (808 nm, 1.0 W cm^−2^)	5	
BPNS‐PEG (3.2 nm diameter)	4T1	70 µg mL^−1^, 24 h	95	[182]
		70 µg mL^−1^, 24 h, 10 min irradiation (808 nm, 2.0 W cm^−2^)	5	
BPNS (208 nm diameter)	HeLa	100 µg mL^−1^, 72 h	90	[183]
		100 µg mL^−1^, 4 h, 10 min irradiation (808 nm, 1.0 W cm^−2^)	25	
BPNS	NIH 3T3	0 to 50 µg mL^−1^, 48 h	>90	[184]
		100 µg mL^−1^, 48 h	60	
		200 to 400 µg mL^−1^, 48 h	<10	
BP quantum dots (3.3 nm diameter)	HeLa	0 to 40.5 µg mL^−1^, 24 h	100	[185]
	LO2		100	
	HeLa	1.6 µg mL^−1^, 4 h	100	
		1.6 µg mL^−1^, 4 h, 4 min irradiation (670 nm, 0.16 W cm^−2^)	70	
		1.6 µg mL^−1^, 4 h, 16 min irradiation (670 nm, 0.16 W cm^−2^)	20	
		1.6 µg mL^−1^, 4 h, 16 min irradiation (670 nm, 0.08 W cm^−2^)	60	
BPNS	A549	50 µg mL^−1^, 24 h	35	[186]
BPNS (200 nm diameter)	U251	0 to 100 µg mL^−1^, 72 h	>95	[187]
	HUVEC			
	4T1			
	LLC			
BPNS (500 nm diameter)	Beas‐2B	100 µg mL^−1^, 24 h	80	[188]
		100 µg mL^−1^, 48 h	40	
BPNS (900 nm diameter)	NIH3T3	1.0 µg mL^−1^, 24 h	94	[189]
	nHDF		>98	
	HT1080		92	
	NIH3T3	125 µg mL^−1^, 24 h	25	
	nHDF		72	
	HT1080		40	
BPNS (960 nm diameter)	L‐929	125 µg mL^−1^, 24 h	30	[76]
BPNS (209 nm diameter)	NIH 3T3	50 µg mL^−1^, 24 h	<5	[79]
	HCoEpiC		60	
	293T		0	
BPNS (426 nm diameter)	NIH 3T3		55	
	HCoEpiC		75	
	293T		10	
BPNS (884 nm diameter)	NIH 3T3		85	
	HCoEpiC		105	
	293T		90	
BPNS (394 nm diameter)	LO2	50 µg mL^−1^, 48 h	90	[190]
BPNS (118 nm diameter)			87	
BPNS (4.5 nm diameter)			82	
BPNS	*E. coli*	500 µg mL^−1^, 3 h	25	[82a]
	*S. aureus*		0	
	LO2	500 µg mL^−1^, 48 h	100	
Single‐layer MoS_2_ (200–1000 nm diameter)	Human embryonic lung fibroblast	10 µg mL^−1^, 24 h	85	[191]
		25 µg mL^−1^, 24 h	80	
Single‐layer MoS_2_‐BSA (200–2500 nm diameter)		10 µg mL^−1^, 24 h	93	
		25 µg mL^−1^, 24 h	90	
MoS_2_	HeLa	30 µg mL^−1^, 24 h	90	[192]
		120 µg mL^−1^, 24 h	75	
MoSe_2_		30 µg mL^−1^, 24 h	80	
		120 µg mL^−1^, 24 h	60	
WS_2_		30 µg mL^−1^, 24 h	100	
		120 µg mL^−1^, 24 h	80	
WSe_2_		30 µg mL^−1^, 24 h	90	
		120 µg mL^−1^, 24 h	80	
Pristine WS_2_ (510 nm diameter)	T24	100 µg mL^−1^, 72 h	90	[193]
WS_2_‐COOH (370 nm diameter)			90	
MoS_2_	A549	200 µg mL^−1^, 24 h	68	[97]
WS_2_			93	
WSe_2_			50	
WS_2_	4T1	100 µg mL^−1^, 24 h	50	[100]
	HeLa		45	
	293T		45	
WS_2_‐PEG (50–100 nm diameter)	4T1		92	
	HeLa		95	
	293T		90	
	4T1	100 µg mL^−1^, 24 h, 5 min irradiation (808 nm, 0.8 W cm^−2^)	<10	
MoS_2_‐PEG (80 nm diameter)	4T1	0–500 µg mL^−1^, 4 h	>100	[88d]
		250 µg mL^−1^, 4 h, 5 min irradiation (808 nm, 1 W cm^−2^)	25	
	L929	0–500 µg mL^−1^, 24 h	>95	
WS_2_ quantum dots (3–4 nm diameter)	LN‐229	80 µg mL^−1^, 24 h	95	[194]
		80 µg mL^−1^, 48 h	90	
MoSe_2_‐PPP (165 nm diameter)	MDA‐MB‐231	30 µg mL^−1^, 5 min irradiation (808 nm, 1 W cm^−2^)	20	[195]
VS_2_ quantum dots (3.1 nm diameter)	HeLa	0‐400 µg mL^−1^, 24 h	>85	[196]
MoS_2_	KB	0‐400 µg mL^−1^, 24 h	>80	[12d]
	Panc‐1		>85	
MoS_2_‐CS	KB		>85	
	Panc‐1		>100	
	KB	20 µg mL^−1^, 4 h, 7 min irradiation (808 nm, 1 W cm^−2^)	55	
	Panc‐1		50	
MoS_2_‐CS‐DOX	KB	20 µg mL^−1^, 4 h	60	
	Panc‐1		35	
	KB	20 µg mL^−1^, 4 h, 7 min irradiation (808 nm, 1 W cm^−2^)	40	
	Panc‐1		10	
MoS_2_‐CS	HCT‐116	10 µg mL^−1^, 1 h	90	[197]
		10 µg mL^−1^, 1 h, 5 min irradiation (808 nm, 1 W cm^−2^)	45	
MoS_2_‐CS‐Cype		10 µg mL^−1^, 1 h	88	
		10 µg mL^−1^, 1 h, 5 min irradiation (808 nm, 1 W cm^−2^)	10	
MoS_2_‐CS	HDF	0–400 µg mL^−1^, 72 h	>85	[198]
MoS_2_	HepG2	0–30 µg mL^−1^, 24 h	>90	[199]
MoS_2_ quantum dots (1–4.5 nm diameter)	*E. coli*	50 µg mL^−1^, 1.5 h, under light irradiation	3	[200]
WS_2_ quantum dots (2–18 nm diameter)			4	
Bulk MoS_2_	Fibroblast	10 µg mL^−1^, 24 h	25	[80]
Single‐layer MoS_2_‐PAA			30	
Single‐layer MoS_2_‐PVP			30	
Single‐layer MoS_2_‐BSA			70	
WS_2_	HeLa	0–200 µg mL^−1^, 24 h	>85	[96]
WS_2_‐BSA				
MoS_2_ (exfoliated with MeLi)	A549	400 µg mL^−1^, 24 h	63	[201]
MoS_2_ (exfoliated with n‐BuLi)			58	
MoS_2_ (exfoliated with t‐BuLi)			53	
Single‐layer MoS_2_	*E. coli* DH5α	80 µg mL^−1^, 2 h	8	[202]
MoS_2_	Amp^r^ *E. coli*	100 µg mL^−1^, 4 h	70	[203]
	B. subtilis		30	
N‐doped MoS_2_	Amp^r^ *E. coli*		6	
	B. subtilis		15	
WS_2_	Amp^r^ *E. coli*		35	
	B. subtilis		25	
N‐doped WS_2_	Amp^r^ *E. coli*		15	
	B. subtilis		20	
Ti_3_C_2_T_x_	*E. coli*	250 µg mL^−1^, 6 h	>90	[127e]
	HeCaT	250 µg mL^−1^, 24 h	88	
	A375		100	
Ti_3_C_2_T_x_‐PLL	*E. coli*	250 µg mL^−1^, 6 h	<5	
	HeCaT	250 µg mL^−1^, 24 h	80	
	A375		82	
Ti_3_C_2_T_x_	*E. coli*	50 µg mL^−1^, 4 h	20	[127a]
	*B. subtilis*		8	
TiVCT_x_	*E. coli*	100 µg mL^−1^, 4 h	<1	[127f]
	*E. coli*	40 µg mL^−1^, 30 min, 5 min irradiation (808 nm, 0.1 W cm^−2^)	0	
	*B. subtilis*		<0.2	
Ti_2_NT_x_	MCF‐10A	250 µg mL^−1^, 24 h	82	[129]
	MCF‐7		40	
	HaCaT		80	
	A375		58	
Ti_3_C_2_T_x_	MCF‐10A	250 µg mL^−1^, 24 h	84	[204]
	MCF‐7		80	
	HaCaT		72	
	A375		60	
Ti_3_C_2_T_x_ sonicated	MCF‐10A		75	
	MCF‐7		63	
	HaCaT		81	
	A375		50	
Ti_3_C_2_T_x_ sonicated and thermally oxidated	MCF‐10A		70	
	MCF‐7		47	
	HaCaT		80	
	A375		30	
Ti_3_C_2_T_x_	MSU1.1	400 µg mL^−1^, 48 h	92	[205]
	HeLa		92	
Few‐layer Ti_3_C_2_T_x_	MSU1.1		90	
	HeLa		91	
Single‐layer Ti_3_C_2_T_x_	MSU1.1		106	
	HeLa		91	
Ti_3_C_2_T_x_	L929	0–175 µg mL^−1^, 24 h	>90	[206]
		250 µg mL^−1^, 24 h	65	
Ti_3_C_2_T_x_	HaCaT	500 µg mL^−1^, 24 h	83	[207]
	A375		56	
	MRC‐5		67	
	A549		22	
Ti_2_CT_x_	HeLa	50 µg mL^−1^, 24 h	69	[208]
		100 µg mL^−1^, 24 h	51	
		300–500 µg mL^−1^, 24 h	43	
V_2_CT_x_	HaCaT	1 µg mL^−1^, 24 h	98	[209]
	A375		86	
	HaCaT	1 µg mL^−1^, 48 h	74	
	A375		72	
	HaCaT	100 µg mL^−1^, 24 h	70	
	A375		52	
	HaCaT	100 µg mL^−1^, 48 h	42	
	A375		27	
Ti_2_CT_x_‐PEG	MCF‐10A	250 µg mL^−1^, 24 h	70	[210]
	MCF‐7		56	
	HaCaT		77	
	A375		76	
Ti_3_C_2_T_x_	HTR‐8/SVneo	0–12 µg mL^−1^, 24 h	>90	[211]
		25 µg mL^−1^, 24 h	75	
		100 µg mL^−1^, 24 h	30	
Ti‐supported MgAl‐LDH	*E. coli*	Cell dispersion over film, 24 h	70	[137a]
	*S. aureus*		50	
MgAl‐LDH (100 nm diameter)	mESCs	0–40 µg mL^−1^, 24 h	100	[212]
		0–40 µg mL^−1^, 48 h		
MgAl‐LDH	Preosteoblasts	2 µg mL^−1^, 24 h	175	[136c]
		20 µg mL^−1^, 24 h	140	
		200 µg mL^−1^, 24 h	55	
ZnAl‐LDH		2 µg mL^−1^, 24 h	100	
		20 µg mL^−1^, 24 h	100	
		200 µg mL^−1^, 24 h	20	
MgAl‐LDH (100 nm diameter)	A549	500 µg mL^−1^, 1–24 h	100	[135]
MgAl‐LDH (90 nm diameter)	mESCs	0–100 µg mL^−1^, 48 h	100	[213]
MgAl‐LDH	MC3T3‐E1	50 µg mL^−1^, 24 h	150	[134]
	HUVEC		90	
	*E. coli*		140	
	*S. aureus*		120	
MgFe‐LDH	MC3T3‐E1		90	
	HUVEC		105	
	*E. coli*		125	
	*S. aureus*		105	
ZnAl‐LDH	MC3T3‐E1		50	
	HUVEC		75	
	*E. coli*		0	
	*S. aureus*		5	
ZnFe‐LDH	MC3T3‐E1		60	
	HUVEC		100	
	*E. coli*		0	
	*S. aureus*		0	
g‐C_3_N_4_ (surface area 12 m^2^ g^−1^)	*E. coli* K‐12	1000 µg mL^−1^, 4 h, under visible light irradiation	45	[145a]
g‐C_3_N_4_ (surface area 128 m^2^ g^−1^)			25	
g‐C_3_N_4_ (surface area 230 m^2^ g^−1^)			0	
g‐C_3_N_4_ nanosheets	*E. coli*	400 µg mL^−1^, 4 h	85	[145c]
		400 µg mL^−1^, 4 h, under visible light irradiation	0	
Bulk g‐C_3_N_4_			25	
Bulk g‐C_3_N_4_	SaOS2	100 µg mL^−1^, 48 h	50	[147]
	Human foreskin fibroblast cells		100	
g‐C_3_N_4_ (70 nm diameter)	SaOS2	25 µg mL^−1^, 48 h	55	
	Human foreskin fibroblast cells		95	
g‐C_3_N_4_ (280 nm diameter)	A549	100 µg mL^−1^, 4 h	90	[146]
	MDA‐MB‐435		100	
	A549	100 µg mL^−1^, 4 h, 1 h of visible light irradiation	45	
	MDA‐MB‐435		35	
SnS nanosheets	SMCC‐7721	25 µg mL^−1^, 48 h	87	[142]
	B16		90	
	HeLa		95	
	A549		93	
SnS‐PEG	SMCC‐7721	30 µg mL^−1^, 6 h	92	
		30 µg mL^−1^, 6 h, 10 min irradiation (808 nm)	70	
SnS‐PEG‐FA‐DOX		30 µg mL^−1^, 6 h	46	
		30 µg mL^−1^, 6 h, 10 min irradiation (808 nm)	23	
AM nanosheets	4T1	10 µg mL^−1^, 6 h	92	[143b]
		50 µg mL^−1^, 6 h	70	
		100 µg mL^−1^, 6 h	45	
AM‐PEG	A549	50 µg mL^−1^, 24 h	92	[140]
	HeLa		92	
	MCF‐7		96	
	HEK‐293		92	
	PC3		91	
	MCF‐7	50 µg mL^−1^, 1 h	97	
		50 µg mL^−1^, 1 h, 5 min irradiation (808 nm, 0.8 W cm^−2^)	54	
AM‐PEG‐DOX		50 µg mL^−1^, 1 h	62	
		50 µg mL^−1^, 1 h, 5 min irradiation (808 nm, 0.8 W cm^−2^)	10	
Al‐TCPP‐PEG nanosheets	1T4	0–100 µg mL^−1^, 24 h	>98	[150]
		50 µg mL^−1^, 6 min ultrasound (50 kHz, 1.6 W cm^−2^), 24 h	9	

### 2.2 hBN

hBN is a commercially available material with a structure closely related to graphite. It consists of layers of boron and nitrogen atoms with an sp^2^ hybridization in a hexagonal honeycomb‐like structure,^[^ ^38^ ^]^ where each layer contains the same amount of boron and nitrogen. However, instead of an ABA stacking as in graphite (where adjacent layers are staggered), hBN presents an AAA stacking pattern, resulting in the direct stacking of boron and nitrogen atoms from adjacent layers.^[^ ^39^ ^]^ The interaction between layers is mainly due to weak van der Waals forces, but some ionic interactions also exist, both within each layer as well as between layers.^[^ ^39^ ^]^ As a consequence, hBN has a large bandgap making it an electric insulator both across and within the layers.^[^ ^40^ ^]^ Although there is some controversy about whether hBN presents a direct or indirect bandgap,^[^ ^39^ ^]^ its value is well known (5.92 to 5.97 eV).^[^ ^41^ ^]^ The last report by Cassabois et al.^[^ ^41c^ ^]^ gives evidence for an indirect bandgap of 5.955 eV. Bulk hBN also presents a relatively high thermal conductivity at room temperature of ≈ 400 W Km^−1^, whereas that of single‐layer hBN is ≈ 600 W Km^−1^.^[^ ^42^ ^]^ Additionally, hBN nanosheets (hBNNS) present exceptional mechanical properties^[^ ^43^ ^]^; hence, they are usually used to reinforce polymeric matrices.^[^ ^44^ ^]^

**Figure 2:**
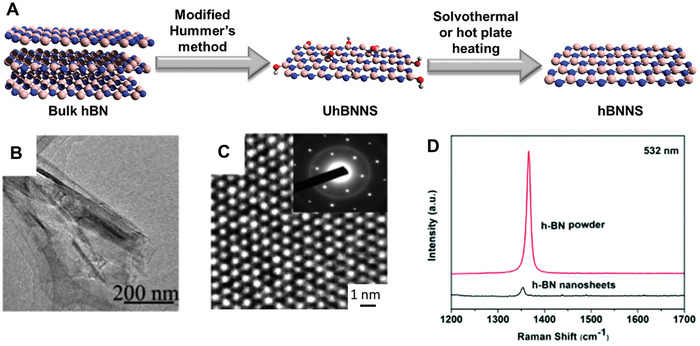
A) Schematic representation of the modified Hummer's method and subsequent reduction for hBNNS production. Color code: blue = N, pink = B, red = O, light gray = H. B) TEM and C) HRTEM (with selected area electron diffraction (SAED) as an inset) images of the hBNNS produced by sonication of bulk hBN in water for 24 h. D) Raman spectra of bulk hBN and hBNNS produced by sonication in water for 24 h. Figures (B–D) Reproduced with permission.^[^ ^45a^ ^]^ Copyright 2016, Royal Society of Chemistry.

Similar to graphene, hBNNS can be synthesized by either bottom‐up or top‐down methods,^[^ ^39^ ^]^ the latter having a greater yield and better scalability. Single‐ and few‐layered hBNNS can be obtained from bulk hBN by liquid exfoliation.^[^ ^45^ ^]^ For example, Lin et al.^[^ ^45d^ ^]^ obtained aqueous suspensions of polyethylene glycol (PEG)‐functionalized single‐ and few‐layered hBNNS. For this, a simple process was used that involved heating bulk hBN powder and PEG at 160–180 °C for 4–6 days under nitrogen flow, followed by sonication and extraction using water. Additionally, the modified Hummer's method (**Figure** **2**A) can also be used to exfoliate bulk hBN.^[^ ^46^ ^]^ In a similar manner to the exfoliation of graphite, this method consists of mixing bulk hBN with KMnO_4_ (1:8 mass ratio) in a solution of concentrated H_2_SO_4_ and H_3_PO_4_ (9:1 volume ratio) at 65 °C for 24 h. After cooling to room temperature, the reaction mixture is added into distilled water ice with 30% H_2_O_2_. Finally, the mixture is purified by washing with water, 30% HCl and ethanol. This results in unreduced hBNNS (UhBNNS) with a thickness of ≈ 3 nm, with hydroxyl and oxygen functionalities that can be eliminated by either solvothermal or hot plate heating.^[^ ^46^ ^]^ These chemical changes during the reduction process can be observed using both FTIR and Raman spectroscopy.

Additionally, there are several approaches to functionalize the surface of hBNNS with hydroxyl^[^ ^47^ ^]^ or amino^[^ ^45^, ^48^ ^]^ groups. For example, Lin et al.^[^ ^45d^ ^]^ obtained hydroxyl‐functionalized hBNNS by sonicating hBN powder in water for 8 h. Using a different approach, Lee et al.^[^ ^47c^ ^]^ reported the synthesis of hydroxyl‐functionalized hBNNS by ball‐milling hBN powder with a 2 m solution of NaOH. The obtained hBNNS‐OH particles had a lateral size of ≈1.5–2 µm and a thickness of 2.62 nm, determined using atomic force microscopy (AFM).^[^ ^47c^ ^]^ Similarly, Lei et al.^[^ ^48^ ^]^ showed that by ball‐milling hBN powder in the presence of urea amino‐functionalized hBNNS (hBNNS‐NH_2_) are obtained.

The biocompatibility of hBNNS depends on several factors, such as cell type, concentration, surface functionalization, and aspect ratio.^[^ ^49^ ^]^ For example, Mateti et al.^[^ ^50^ ^]^ showed that the toxicity of pristine hBNNS toward SaOS‐2 cells is strongly dependent on the diameter of the particles, with the larger particles (e.g., 2.0 µm of diameter) having lower toxicity. Using the electron spin resonance (ESR) technique, they determined that the reason for this is the presence of unsaturated boron atoms at the nanosheet edges or particle surface, which induces the production of reactive oxygen species (ROS), leading to cell death.^[^ ^50^ ^]^ Another study reported that hydroxyl‐functionalized hBNNS (hBNNS‐OH) have no cytotoxic effects toward L929 mouse cells up to a concentration of 200 µg mL^−1^.^[^ ^51^ ^]^ The most important findings of several works regarding the biocompatibility of hBNNS are summarized in Table 1.

### 2.3 BP

**Figure 3:**
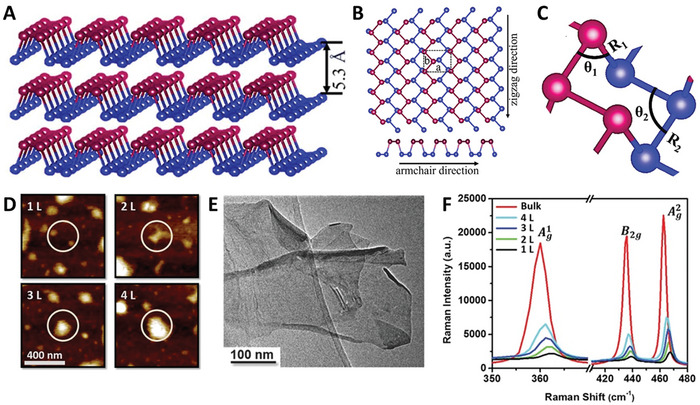
A) Side view of multilayer BP. B) Top view of monolayer BP, with the two special directions indicated. C) Representation of the structural arrangement of phosphorus atoms. (A–C) Reproduced with permission.^[^ ^54^ ^]^ Copyright 2022, IOP Publishing. Characterization, using D) AFM, E) TEM, and F) Raman spectroscopy, of BPNS synthesized as reported by Guo et al.^[^ ^71c^ ^]^ Reproduced with permission.^[^ ^71c^ ^]^ Copyright 2015, Wiley‐VCH.

BP is the most stable allotropic form of phosphorus, discovered more than a century ago.^[^ ^52^ ^]^ It presents a similar structure to graphite: each layer is composed of two sublayers of phosphorus atoms, where each atom binds covalently to three other adjacent atoms.^[^ ^53^ ^]^ Within each sublayer, the phosphorus atoms form parallel zigzag chains, and when taken together, both sublayers form a chair network within each layer^[^ ^54^ ^]^ (**Figure** **3**A). This structure has two special directions: the zigzag direction, defined by the direction along which the zigzag chains run, and the armchair direction, defined by the interconnected chairs within each layer^[^ ^54^ ^]^ (Figure 3B). Remarkable in‐plane anisotropic properties emerge from this unique structural arrangement,^[^ ^55^ ^]^ which contribute to its electrical conductivity,^[^ ^56^ ^]^ and to the optical,^[^ ^57^ ^]^ mechanical,^[^ ^58^ ^]^ and thermoelectric^[^ ^59^ ^]^ properties. Even though the interactions between layers in BP are partly due to the presence of lone pairs of electrons on the phosphorus atoms,^[^ ^60^ ^]^ for simplicity purposes they are often treated as van der Waals interactions.^[^ ^60^, ^61^ ^]^ BP is a direct bandgap material, with a value that strongly depends on the number of layers in the material^[^ ^56^, ^62^ ^]^: ≈0.3 eV for bulk BP, ≈1.88 eV for bilayer and ≈2.0 eV for monolayer BP (often referred to as phosphorene). Additionally, the bandgap of BP can be also controlled by strain,^[^ ^63^ ^]^ doping with heteroatoms,^[^ ^64^ ^]^ external electrical fields,^[^ ^65^ ^]^ and by the configuration of the edges of the nanosheets^[^ ^66^ ^]^ (zigzag or armchair) and their functional groups.^[^ ^67^ ^]^ All in all, the highly tunable nature of the bandgap allows BP to absorb light at wavelengths ranging from IR to UV, making it a potential candidate for photothermal therapy applications.^[^ ^68^ ^]^ Moreover, the degradation products of BP are non‐toxic phosphates, which are an essential component for bone regeneration.^[^ ^69^ ^]^

BP nanosheets (BPNS) can be synthesized using either top‐down or bottom‐up methods. The most commonly used methods for their synthesis have recently been comprehensively reviewed by Sultana et al.^[^ ^70^ ^]^ Since top‐down methods have greater yield and scalability, most of the works where BPNS are used for biomedical applications rely on them. Several liquid‐phase exfoliation methods, using different solvents such as N‐methyl‐2‐pyrrolidone (NMP),^[^ ^71^ ^]^ dimethylformamide (DMF),^[^ ^72^ ^]^ dimethyl sulfoxide (DMSO),^[^ ^72b^ ^]^ and N‐cyclohexyl‐2‐pyrrolidone^[^ ^71a^ ^]^ have been reported. For example, Guo et al.^[^ ^71c^ ^]^ developed a sonication‐assisted liquid‐phase exfoliation process to obtain single‐ to seven‐layered BPNS from bulk BP. The process consists of mixing bulk BP with a saturated solution of NaOH in NMP, followed by sonication for 4 h. Then, a series of centrifugation steps at different speeds were used to separate the thinner BPNS:3000 rpm for 10 min to remove the non‐exfoliated BP, 12000 rpm for 20 min to separate the thick BPNS (5–12 layers), and 18000 rpm for 20 min to separate the thinner BPNS (1‐7 layers). The obtained BP was washed and redispersed with water. With this procedure, hydroxyl‐functionalized few‐layer BPNS were obtained, enhancing the water dispersibility of the samples with respect to pristine BPNS.^[^ ^71c^ ^]^ The samples were then characterized using AFM, TEM, and Raman spectroscopy (Figure 3D–F, respectively). Furthermore, other studies demonstrated that it is possible to obtain few‐layer BPNS by a combination of mechanical exfoliation and plasma etching, showing that the thickness can be controlled by the etching time.^[^ ^73^ ^]^ Another widely used liquid‐phase method in the production of BPNS is electrochemical exfoliation, which offers better yields and higher 2D‐material quality.^[^ ^74^ ^]^

Similar to the already discussed 2D materials, the cytotoxicity of BPNS is strongly related to the cell type, dosage, surface functionalization, and aspect ratio. These aspects and the nano‐bio interactions of BPNS were recently reviewed by Wu et al.^[^ ^75^ ^]^ Song et al.^[^ ^76^ ^]^ reported that by disrupting the cell membrane, BPNS (lateral size of 960 nm) induced the apoptosis in L‐929 fibroblastic cells. A similar effect was also observed for *E. coli* and *B. subtilis*.^[^ ^77^ ^]^ This interaction was recently attributed to the nano‐knife effect.^[^ ^78^ ^]^ Zhang et al.^[^ ^79^ ^]^ demonstrated that BPNS with a smaller lateral size (209 nm) show no toxicity toward several cell lines, while larger particles (884 nm) do. Human serum albumin (HSA) functionalized BPNS (lateral size of 300 nm), synthesized using HSA‐directed exfoliation of bulk BP,^[^ ^80^ ^]^ displayed little toxicity toward U87MG cells, with a cell viability above 80% even when treated for 24 h at a concentration of 500 µg mL^−1^.^[^ ^81^ ^]^ Several authors also showed that the concentration of ROS upon exposure to BPNS is time‐ and dose‐dependent.^[^ ^82^ ^]^ The most important results regarding cell viability of different cell lines upon exposure to BPNS are summarized in Table 1.

### 2.4 TMDCs

**Figure 4:**
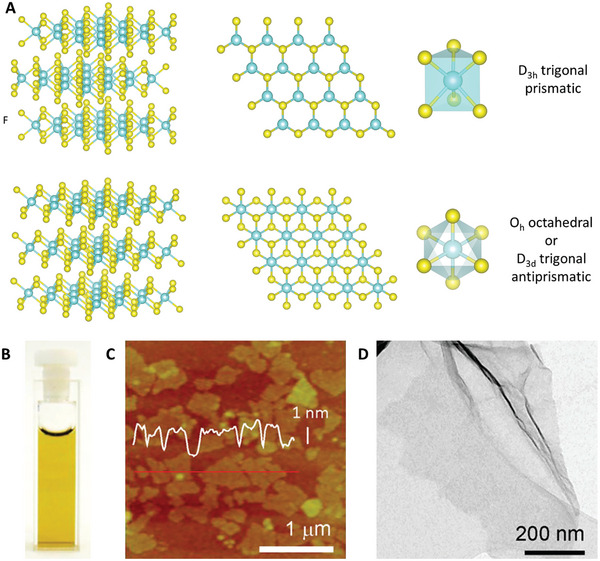
A) Bulk TMDC structure and top view of the structure of a single‐layer TMDCNS with trigonal prismatic and octahedral coordination around the metal atoms. Color code: light blue = metal, yellow = chalcogen. B) Photograph of a water dispersion of MoS_2_ nanosheets obtained by the lithium intercalation method. C) AFM (height profile along the red line is overlaid) and D) TEM images of MoS_2_ nanosheets obtained by the lithium intercalation method. Figures (B–D) Reproduced with permission.^[^ ^91e^ ^]^ Copyright 2011, American Chemical Society.

TMDCs are a group of 2D compounds with diverse properties, that share a general formula MX_2_, where M is a transition metal (usually from groups 4–10, such as Ti, Nb, Ta, Mo, or W) and X is a chalcogen (S, Se, or Te).^[^ ^83^ ^]^ For example, HfS_2_ is an insulating material, while MoS_2_ is a semiconductor and NbS_2_ presents metallic behavior.^[^ ^84^ ^]^ Bulk TMDCs crystallize in a layered structure, leading to strong anisotropy in their electrical, mechanical and thermal properties.^[^ ^85^ ^]^ Each layer is composed of a hexagonal arrangement of metal atoms sandwiched between two layers of chalcogen atoms (**Figure** **4**A), with octahedral or trigonal prismatic coordination geometries around the metal atoms.^[^ ^84^ ^]^ The interactions within layers are predominantly covalent, whereas only weak van der Waals forces keep the layers together, allowing for an easy exfoliation of bulk TMDCs into 2D structures only a few layers thick.^[^ ^86^ ^]^ Semiconducting 2D TMDC are generally direct‐band‐gap semiconductors, where the bandgap value strongly depends on the coordination environment of the metal and its *d*‐electron count.^[^ ^84^ ^]^ There is only a minor effect of the chalcogen atom on the electronic structure, but a trend can be identified nevertheless: for increasing chalcogen atomic numbers, a broadening of the *d* bands and decreasing bandgaps are observed.^[^ ^84^ ^]^ For example, the bandgap of MoX_2_ decreases from 1.3 to 1.0 eV when changing the chalcogen atom from S to Te.^[^ ^87^ ^]^ The high photothermal conversion coefficient of 2D TMDCs make them promising candidates for photothermal therapy applications.^[^ ^12^, ^88^ ^]^ Additionally, several studies have shown the potential of 2D TMDCs as drug delivery systems.^[^ ^12^, ^88^, ^89^ ^]^

In order to obtain large quantities of single‐ or few‐layer TMDC nanosheets (TMDCNS), liquid‐phase exfoliation methods are the most adequate approach.^[^ ^90^ ^]^ Lithium intercalation has been widely used for this purpose.^[^ ^91^ ^]^ For example, Joensen et al.^[^ ^91a^ ^]^ reported a method to synthesize single‐layer MoS_2_ from the bulk material. First, bulk MoS_2_ is soaked in a 1.6 m solution of n‐butyl lithium in hexane for at least 48 h under argon atmosphere, after which the solid is washed with hexane and dried. Immediately after, the solid is immersed in water and the suspension is sonicated for 1 h. The mixture is then centrifuged and washed with water several times in order to remove the formed LiOH as well as the unexfoliated material. As a result, a water suspension of nanosheets (Figure 4B) with a lateral dimension of 300–800 nm (determined using TEM, Figure 4D) and a thickness of 1.0–1.2 nm (determined using AFM, Figure 4C) are obtained.^[^ ^91e^ ^]^ This method is known to yield electronic properties that differ from the pristine 2D material due to structural changes, but it has been reported that an annealing of the as‐synthesized material at 300 °C for 1 h restores the semiconducting properties.^[^ ^91e^ ^]^ Similar to other 2D materials, few‐layer TMDCNS can also be obtained by a simple solvent exfoliation process.^[^ ^92^ ^]^ Using this method, Coleman et al.^[^ ^92a^ ^]^ conducted a solvent survey using more than 20 solvents for the exfoliation of MoS_2_ and WS_2_. The general procedure consisted of sonicating the bulk material in the solvent for 1 h, followed by centrifugation at 500 rpm for 90 min to remove the remaining unexfoliated and thicker materials. They concluded that good dispersibility is achieved when the surface tension of the solvent is in the range of 30–40 mJ m^−2^, N‐vinyl‐2‐pyrrolidone (NVP) and DMSO being the best solvents for the exfoliation of MoS_2_ and WS_2_, respectively.^[^ ^92a^ ^]^

Surface modification is also possible in order to enhance the dispersibility or biocompatibility, or to add additional functionality to TMDCNS.^[^ ^93^ ^]^ For example, Liu et al.^[^ ^88b^ ^]^ reported a simple method to obtain MoS_2_ nanosheets functionalized with PEG and PEG‐folic acid (PEG‐FA). The procedure was simple: lipoic‐acid‐functionalized PEG (LA‐PEG) or folic‐acid‐conjugated LA‐PEG (LA‐PEG‐FA) was added to an aqueous suspension of MoS_2_ nanosheets, sonicated for 20 min and stirred overnight (excess PEG was then removed by centrifugal filtration). The obtained MoS_2_‐PEG and MoS_2_‐PEG‐FA particles displayed enhanced dispersibility in water with respect to pristine MoS_2_ nanosheets.^[^ ^88b^ ^]^ Additionally, doxorubicin‐loaded PEGylated MoS_2_ nanosheets were also obtained by stirring a mixture of the nanosheets and doxorubicin for 24 h, followed by filtration and rinsing with water several times.^[^ ^88b^ ^]^ Other similar approaches have also been used to functionalize TMDCNS with PEG‐FA.^[^ ^94^ ^]^ Furthermore, several other molecules can be used to functionalize the surface of TMDCNS, such as chitosan (CS),^[^ ^12d^ ^]^ thiobarbituric acid,^[^ ^95^ ^]^ and bovine serum albumin (BSA).^[^ ^80^, ^96^ ^]^

The cytotoxicity of TMDCNS is, in general, lower than that of graphene.^[^ ^97^ ^]^ In particular, it has been reported that the nano‐knife effect (the efflux of intracellular materials due to the penetration of a sharp‐edged material in the cell membrane) plays almost no role in the toxicity of this group of 2D materials.^[^ ^98^ ^]^ Additionally, there is a large variability in the cytotoxicity of different non‐functionalized TMDCNS. For example, the cytotoxicity of MoS_2_, WS_2_, and WSe_2_ toward A549 cells follow the order WS_2_ < MoS_2_ < WSe_2_.^[^ ^97^ ^]^ It is believed that, since the chalcogen atoms are located in the surface of the nanosheets allowing more interaction of these atoms with the cells, selenium might be more toxic than sulfur in these materials, as is the case of H_2_Se and H_2_S.^[^ ^99^ ^]^ Surface functionalization also plays an important role in the cytotoxicity of TMDCNS. Yin et al.^[^ ^12d^ ^]^ reported a significant improvement in the biocompatibility of MoS_2_ nanosheets toward KB and Panc‐1 cells when functionalized with chitosan. Similarly, PEG‐functionalized WS_2_ nanosheets show almost no toxicity toward 4T1, HeLa and 293T cells, even at high concentrations, while non‐functionalized nanosheets do.^[^ ^100^ ^]^ Lastly, Guan et al.^[^ ^80^ ^]^ reported that bovine serum albumin (BSA) induces the exfoliation of bulk MoS_2_, producing highly biocompatible MoS_2_‐BSA nanosheets. The most important reports regarding cytotoxicity of TMDCNS to different cell lines are summarized in Table 1.

### 2.5 MXenes

**Figure 5:**
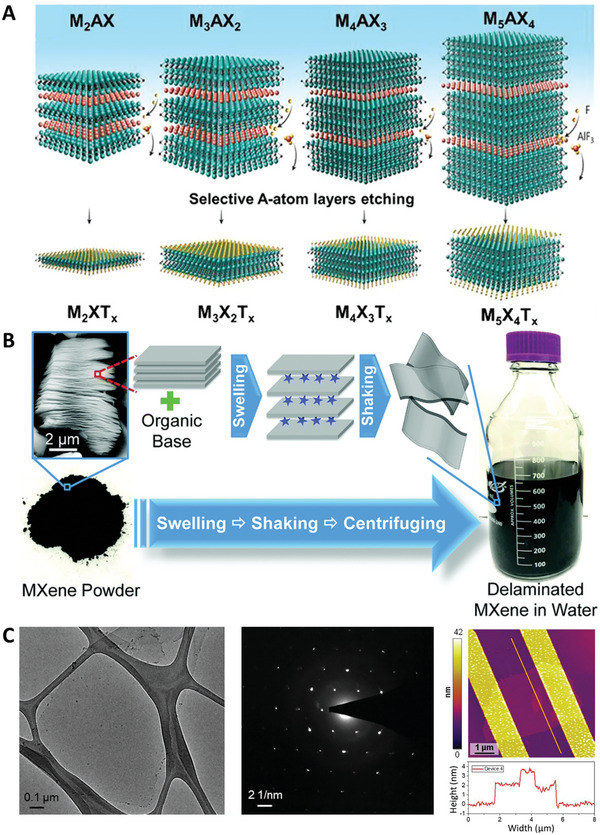
A) Schematic representation of different MAX phases (M_2_AX, M_3_AX_2_, M_4_AX_3_, M_5_AX_4_) and the corresponding MXenes (M_2_XT_x_, M_3_X_2_T_x_, M_4_X_3_T_x_, M_5_X_4_T_x_), obtained after selective etching of the A‐atom layers. Reproduced with permission.^[^ ^101^ ^]^ Copyright 2022, American Chemical Society. B) Schematic representation of the delamination process with an organic base. Reproduced with permission.^[^ ^123^ ^]^ Copyright, Royal Society of Chemistry. C) TEM image, selected area electron diffraction (SAED) pattern and AFM image (height profile along the orange path is shown below) of Ti_3_CNT_x_ MXene synthesized using LiF and HCl. Reproduced with permission.^[^ ^113^ ^]^ Copyright 2019, American Chemical Society.

MXenes are a group of 2D materials derived from 3D bulk materials called MAX phases.^[^ ^101^ ^]^ The general formula of a MAX phase is M_n+1_AX_n_, where M is an early transition metal (Sc, Ti, Zr, Hf, V, Nb, etc.), A is an element from group 13 or 14, and X is C and/or N.^[^ ^102^ ^]^ A general representation of their crystal structure is shown in **Figure** **5**A. Up to date, more than 155 MAX phases have been reported.^[^ ^102^ ^]^ There is a wide variety of methods to synthesize these MAX phases, such as physical^[^ ^103^ ^]^ and chemical vapor deposition,^[^ ^104^ ^]^ combustion synthesis,^[^ ^105^ ^]^ high‐temperature self‐propagating synthesis,^[^ ^106^ ^]^ sintering,^[^ ^107^ ^]^ and so on. Additionally, many MAX phases are commercially available. The M─A bonds in MAX phases are chemically active metallic bonds, which are easier to break than the M─X bonds.^[^ ^108^ ^]^ Because of this, etching agents can be used to selectively break M‐A bonds, leading to MX layers (MXenes),^[^ ^109^ ^]^ as shown in Figure 5A. Depending on the etching method used, different functional groups (─O, ─F, ─OH, etc.) are left on the surface of MXenes,^[^ ^110^ ^]^ often denoted as T_x_.

Pristine MXenes are metallic compounds.^[^ ^101^ ^]^ However, the surface functionalization produced by etching can alter their electronic properties making them semiconducting materials.^[^ ^101^ ^]^ For example, Ti_2_CT_2_ MXene is a metallic compound when the surface is functionalized with ─F (T = ─F) or ─OH (T = ─OH) groups, while it is a semiconductor with a bandgap of 0.33 eV if ─O functional groups are in the surface.^[^ ^111^ ^]^ Since the optical properties are strongly related to the electronic properties, they can also be tuned by surface functionalization.^[^ ^112^ ^]^ Additionally, substitutions on either the M or X sites allow for further tuning of the optical properties of MXenes.^[^ ^113^ ^]^ The highly tunable nature of their electronic and optic properties, high hydrophilicity, flexibility and light‐to‐heat conversion performance, make MXenes promising candidates for numerous biomedical applications, including cancer treatment and bone regeneration.^[^ ^114^ ^]^

The different synthesis methods of MXenes have recently been comprehensively reviewed by Murali et al.^[^ ^101^ ^]^ Briefly, the available methods for the etching of MAX phases can be divided into two categories: HF‐based methods and HF‐free methods. In the first category, the A layers of MAX phases are selectively etched by HF, either directly added as such or produced in situ.^[^ ^110^, ^115^ ^]^ For example, Naguib et al.^[^ ^109^ ^]^ reported that Ti_3_C_2_T_x_ (T_x_ = ─F and ─OH) can be synthesized from Ti_3_AlC_2_ by simply immersing it into a 50% HF aqueous solution for 2 h, followed by washing with water and centrifugation. Ghidiu et al.^[^ ^116^ ^]^ achieved the etching of Ti_3_AlC_2_ by in situ‐produced HF, by using a mixture of LiF and HCl, yielding Ti_3_C_2_T_x_ as a product. Energy‐dispersive spectroscopy (EDS) and X‐ray photoelectron spectroscopy (XPS) of the obtained MXene suggest a combination of ─O and ─F groups on the surface. Using a hydrothermal method, Wang et al.^[^ ^117^ ^]^ synthesized Ti_3_C_2_T_x_ (T_x_ = ─F, ─O, and ─OH) by heating an aqueous mixture of Ti_3_AlC_2_ powder and NH_4_F at 150 °C for 24 h. To avoid using HF, Li et al.^[^ ^118^ ^]^ reported the synthesis of Ti_3_C_2_T_x_ (T_x_ = ─OH and ─O) by hydrothermal reaction of Ti_3_AlC_2_ and a 27.5 m NaOH solution at 270 °C under argon atmosphere. Molten salt assisted etching of MAX phases can also be used to produce MXenes. For instance, Ti_2_CCl_2_^[^ ^119^ ^]^ and Ti_3_C_2_Cl_2_^[^ ^120^ ^]^ (containing exclusively ─Cl groups on their surfaces), were obtained by using molten ZnCl_2_ as the etching agent.

In order to obtain single‐ or few‐layer MXenes, exfoliation of the as‐synthesized MXenes is necessary. In this regard, taking advantage of the negatively charged surface of the layers,^[^ ^121^ ^]^ several approaches have been used: intercalation of polar organic solvents,^[^ ^121^, ^122^ ^]^ large organic bases,^[^ ^113^, ^123^ ^]^ and metal cations.^[^ ^124^ ^]^ After intercalation of organic molecules or cations, stirring or sonication in water produces a colloidal dispersion of single‐ or few‐layer MXenes^[^ ^115^, ^121^, ^123^ ^]^ (Figure 5B). Further surface modification is also possible.^[^ ^125^ ^]^ For example, Lin et al.^[^ ^126^ ^]^ functionalized the surface of Ti_3_C_2_T_x_ nanosheets with soybean phospholipids (SBP), obtaining highly biocompatible Ti_3_C_2_T_x_‐SBP nanosheets.

Several studies regarding biocompatibility and cytotoxicity of MXenes toward both eukaryotic and prokaryotic cells have been reported; a recent systematic review by Sagadevan et al.^[^ ^114^ ^]^ on this subject summarizes the most relevant works. MXenes have been widely studied as antibacterial agents, showing promising results for several prokaryotic cells, both for Gram positive and Gram negative bacteria, such as *E. coli*,^[^ ^127^ ^]^ *S. aureus*,^[^ ^127a,c,d^ ^]^ and *B. subtilis*.^[^ ^127a,b,d,f^ ^]^ For example, Rozmysłowska‐Wojciechowska et al.^[^ ^127e^ ^]^ showed that Ti_3_C_2_T_x_ has almost no antibacterial activity toward *E. coli*, whereas Ti_3_C_2_T_x_ functionalized with poly‐L‐lysine (PLL) reduces the living cell population by approximately two orders of magnitude when used at a concentration of 200 µg mL^−1^ for 6 h. Similarly, several studies have been published regarding the biocompatibility of MXenes. Zhang et al.^[^ ^128^ ^]^ studied the cytotoxic effects of Ti_3_C_2_T_x_ toward HUVEC cells at different concentrations. They reported that increased doses of MXene influenced the tricarboxylic acid pathway within the mitochondria, leading to a disruption in mitochondrial function. Additionally, Ti_2_NT_x_ MXene shows no cytotoxic effects toward non‐malignant cells (MCF‐10A and HeCaT), whereas a dose‐dependent toxicity is observed toward cancerous cells (MCF‐7 and A375).^[^ ^129^ ^]^ The main mechanisms of toxicity are the generation of ROS and the internalization of Ti_2_NT_x_ nanosheets. Some of the most important works regarding cytotoxicity of MXenes are summarized in Table 1.

### 2.6 Other 2D Materials

Beyond the 2D materials mentioned so far, several other 2D materials have been reported to have relevant properties for biomedical applications in the field of bone therapy. Among them are layered double hydroxides^[^ ^130^ ^]^ (LDHs), graphitic carbon nitride^[^ ^131^ ^]^ (g‐C_3_N_4_), several BP analogs,^[^ ^132^ ^]^ and 2D metal‐organic frameworks^[^ ^133^ ^]^ (MOFs). In this section, a brief description of these materials and their properties will be given. The biocompatibility and cytotoxicity of these materials are presented in Table 1.

**Figure 6:**
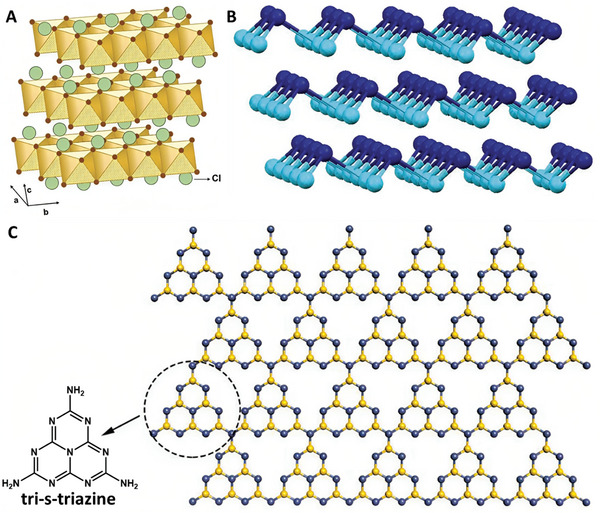
Structure of other relevant 2D materials with potential biomedical applications in the field of bone defect regeneration. A) Structure of a general LDH. Reproduced with permission.^[^ ^136c^ ^]^ Copyright 2017, Wiley‐VCH. B) Structure of antimonene. Reproduced with permission.^[^ ^139^ ^]^ Copyright 2016, Wiley‐VCH. C) Structure of g‐C_3_N_4_, with tri‐s‐triazine as the building block. Reproduced with permission.^[^ ^131a^ ^]^ Copyright 2021, Elsevier B.V. All rights reserved.

LDHs are a group of layered clays with the general formula [M^II^_1‐x_M^III^_x_(OH)_2_](A^n−^)_x/n_, composed of hydroxide layers of divalent and trivalent metal cations and interlayer anions A^n−^ (**Figure** **6**A).^[^ ^130^ ^]^ Pristine LDHs have similar elastic moduli (9–35 GPa) to cortical bone (5–23 GPa), which makes them good candidates for bone tissue engineering.^[^ ^134^ ^]^ Additionally, they are highly biocompatible, presenting low toxicity toward several cell lines.^[^ ^135^ ^]^ LDHs have been reported to have osteogenic,^[^ ^136^ ^]^ antibacterial^[^ ^137^ ^]^ and angiogenic properties.^[^ ^136^, ^138^ ^]^ The positively charged surface of LDHs further allows the loading of negatively charged drugs, providing additional functionality.^[^ ^130^ ^]^

Several BP analogs have also attracted attention in recent years due to their low toxicity and similar characteristics to BP.^[^ ^132b^ ^]^ These analog materials present higher surface areas^[^ ^139^ ^]^ and higher carrier mobilities^[^ ^140^ ^]^ than BP, as well as excellent stability.^[^ ^141^ ^]^ Some examples of these materials are tin sulfide^[^ ^141^, ^142^ ^]^ and antimonene^[^ ^139^, ^140^, ^143^ ^]^ (Figure 6B). Xie et al.^[^ ^142^ ^]^ developed PEGylated tin sulfide nanosheets, which showed great biocompatibility with SMCC‐7721 cells and higher doxorubicin loading capacity than BP. Based on PEGylated antimonene (AM) nanosheets, Tao et al.^[^ ^140^ ^]^ developed a photonic drug‐delivery platform for cancer treatment. This platform exhibited excellent photothermal properties, NIR‐controlled drug release and high drug‐loading capacity.

g‐C_3_N_4_, a metal‐free semiconductor material, has a graphene‐like structure, with sp^2^ carbon and nitrogen atoms forming aromatic C‐N six‐membered heterocycles^[^ ^131^ ^]^ (Figure 6C). The basic building block of g‐C_3_N_4_ can be either s‐triazine or tri‐s‐triazine, leading to two different structural isomers – the one based on tri‐s‐triazine being the most stable one.^[^ ^131a^ ^]^ It has been found that g‐C_3_N_4_ has antibacterial activity, which is due to two main effects: the nano‐knife effect^[^ ^144^ ^]^ and the photo‐induced production of ROS.^[^ ^145^ ^]^ Moreover, the latter is an interesting property for other applications such as the treatment of cancer.^[^ ^131b^ ^]^ Both Taheri et al.^[^ ^146^ ^]^ and Davardoostmanesh et al.^[^ ^147^ ^]^ demonstrated the potential application of g‐C_3_N_4_ in the treatment of different types of cancer. Finally, it is also possible to load g‐C_3_N_4_ with drugs, incorporating additional functionality or improving its intrinsic therapeutic properties.^[^ ^131c^ ^]^

MOFs are compounds constructed by the combination of organic linkers and metal ions, producing extended networks with pores of adjustable size and shape.^[^ ^133^ ^]^ Their high porosity, high surface areas and structural designability have attracted attention for several biomedical applications, such as targeted drug delivery^[^ ^148^ ^]^ and cancer treatment.^[^ ^149^ ^]^ 2D MOFs combine the general advantages of 2D materials with the high tunability of traditional MOFs. For example, Li et al.^[^ ^150^ ^]^ developed highly biocompatible PEG‐coated Al‐containing MOF nanosheets, using tetrakis(4‐carboxyphenyl)porphyrin (TCPP) as the linker molecule. The Al‐TCPP‐PEG nanosheets acted as sonosensitizers for sonodynamic cancer therapy, showing cell viabilities (1T4 cells) close to 100% for all tested concentrations and less than 10% when exposed to ultrasonic treatment for 6 min (for a concentration of 50 µg mL^−1^).

### 2.7 In Vitro and In Vivo Toxicity of 2D Materials

Even though 2D materials have several potential biological applications, their toxic effects may affect human health.^[^ ^151^ ^]^ Several reviews have been published in the last years addressing this issue.^[^ ^11^, ^152^ ^]^ In this section, a short summary of the main reasons for the toxicity of 2D materials and how their biocompatibility can be enhanced is presented.

The in vitro cytotoxicity of different families of 2D materials has already been discussed in previous sections. In summary, the cytotoxicity of these materials can usually be attributed to one or both of the following effects: generation of ROS, and cell membrane disruption from the increased stress induced by the sharp edges of the nanosheets (sometimes referred to as the nanoknife or nanoblade effect). As discussed before, the in vitro cytotoxicity of 2D materials can be reduced, in general, by tunning the size of the nanosheets,^[^ ^79^ ^]^ controlling the concentration in the culture medium,^[^ ^82^, ^153^ ^]^ functionalizing them with different groups,^[^ ^12^, ^37^, ^51^, ^100^ ^]^ exfoliating with biomolecule‐assisted methods,^[^ ^80^, ^81^ ^]^ or encapsulating the 2D material inside polymeric matrices.^[^ ^154^ ^]^

In order to balance optimal therapeutic results with minimal side effects, it is crucial to target the delivery of 2D materials to diseased areas to reach therapeutic concentrations, while ensuring these materials degrade and clear from the body sufficiently to prevent prolonged toxicity. Hence, understanding the in vivo biodistribution and fate of 2D materials becomes a matter of great importance. Many reviews have already addressed the issue of in vivo biodistribution, degradability and toxicity of different 2D materials.^[^ ^152^, ^155^ ^]^

The challenges for 2D materials in biomedical applications involve overcoming two main barriers^[^ ^152a^ ^]^: a) the formation of a protein corona which can reduce circulation time and block targeting ligands present on the surface of nanomaterials^[^ ^156^ ^]^; b) the mononuclear phagocytic system (MPS), which includes monocytes and macrophages, tends to sequester most administered nanomaterials, primarily accumulating them in the liver in Kupffer cells.^[^ ^157^ ^]^ Addressing these challenges involves strategies such as size tuning, surface modification, and element composition optimization, among others.^[^ ^152a^ ^]^ For example, Cao et al.^[^ ^158^ ^]^ compared the in vivo behavior of PEGylated GO nanosheets of different sizes. Smaller nanosheets (28 ± 10 nm) showed 2–5 times higher accumulation at tumor sites compared to larger ones (91 ± 34 nm). Additionally, they also showed faster liver‐related clearance. PEGylation of few‐layered graphene was also reported to reduce histological abnormalities in comparison to unmodified graphene, during three months in vivo.^[^ ^159^ ^]^ In another study, Zeng et al.^[^ ^160^ ^]^ coated polydopamine onto BP nanosheets to enhance their stability and reduce their degradation in vivo. After 3 days in water, the photothermal performance of the modified BP was attenuated less compared to bare BP, indicating slowed degradation due to reduced contact with oxygen facilitated by the polydopamine coating.

Last but not least, various reports have also indicated that embedding 2D materials within 3D hydrogel matrices enhances their stability and retention at the application site.^[^ ^15b^ ^]^ This approach could not only enhance the therapeutic efficiency of 2D materials, but also reduce the quantity used thanks to the enhanced retention, eventually leading to reduced off‐site and long‐term toxicities.

## 3 2D Materials‐Based Hydrogels

### 3.1 Bulk Hydrogels

Bulk hydrogels are macroscopic hydrogels with defined shapes. Due to their macroscopic size and preformed shape, they often require surgical intervention for implantation into a patient. However, they have the advantage of being easily fabricated, compared to other more complex hydrogel architectures.

Bulk hydrogels containing 2D materials are very versatile systems, usually composed of a polymeric matrix containing uniformly dispersed inorganic nanosheets. The large number of variables that can be controlled, such as the type and concentration of the polymer and of the 2D material, as well as the inclusion of additional components, leads to an extremely wide range of properties and potential applications that can be achieved with these systems^[^ ^15^, ^214^ ^]^ (**Table** **2**). 2D materials‐based bulk hydrogels are generally synthesized in a simple two‐step process. First, the 2D material is dispersed in a precursor solution of the polymer or its monomer, and then gelation is induced to produce the bulk hydrogel.^[^ ^5^, ^215^ ^]^

**Table 2:** Some representative examples of recently synthesized hydrogels incorporating 2D materials, their composition, architecture and main properties.

Polymer matrix	2D material	Additional components	Architecture	Key properties	References
Regenerated silk fibroin	Ti_3_C_2_ MXene	–	Bulk	Electrical conductivity, promotes bone regeneration	[5e]
Poly(N‐isopropylacrylamide)	BP	Mesenchymal stem cell derived exosomes	Bulk	NIR‐controlled release of exosomes, promotes bone regeneration	[5d]
Agarose	BP@PEG	Emetine	Bulk	PTT, NIR‐controlled release of emetine and sensitization of tumors to PTT	[215a]
Agarose	Ti_3_C_2_T_x_ MXene	Doxorubicin	Bulk	NIR‐controlled release of doxorubicin	[215b]
None	Ti_3_C_2_T_x_ MXene, rGO	Ethylenediamine (as cross‐linker)	Bulk	Cell adhesion, spreading and proliferation promising for tissue engineering	[219]
Maleilated chitosan	GO	β‐TCP nanoparticles, maleic anhydride (as cross‐linker) L‐cysteine (zwitterionic)	Bulk	Promotes bone regeneration	[215c]
DNA	WS_2_‐alginate	PEG diepoxide (as cross‐linker), Ca^2+^ (as cross‐linker)	Bulk	Mechanically tough, promising for medical implants	[215d]
Polypeptide	BP	Bufalin	Bulk	PTT and NIR‐controlled release of bufalin	[215e]
GelMA, PEG	BP@Mg	β‐TCP nanoparticles	Bilayer	Neurovascularized bone regeneration via periosteum biomimetic structure	[220]
GelMA, sodium alginate	Ti_3_C_2_ MXene	β‐TCP nanoparticles, Sr^2+^ (as cross‐linker)	3D‐printed grid	Shear thinning bioink, antimicrobial activity, promotes bone regeneration	[14a]
GelMA	rGO	–	3D‐printed grid	Shear thinning bioink, neuralized bone regeneration	[221]
DNA	BP	VEGF, PCL scaffold	Hydrogel‐filled 3D‐printed grid scaffold	Shear thinning hydrogel, mechanically strong scaffold, VEGF release, promotes vascularized bone regeneration for irregular defects	[222]
GelMA, DMA	PDA‐BP	Doxorubicin, PPENK substrate	Coating	Adhesive, promotes bone regeneration, NIR‐controlled photothermal antibacterial and anticancer activity	[9b]
GelMA	Ti_3_C_2_ MXene	Tobramycin, PDA, SP substrate	Coating	Antibacterial and photothermal anticancer activity, promotes bone regeneration	[223]
Silk fibroin	GO	Bone marrow mesenchymal stem cells, H_2_O_2_ and horseradish peroxidase (for cross‐linking)	In situ gelling	Injectable, promising for bone regeneration	[224]
OPF	BP	CNTpega, ammonium persulfate (cross‐linking initiator), L‐ascorbic acid (cross‐linking accelerator)	In situ‐gelling	Injectable, electrically conductive, mechanically strong, excellent bone regeneration under electrical stimulation	[225]
Alg‐MA, Alg‐DA	PDA‐modified Ti_3_C_2_ MXene	–	In situ‐gelling	Antibacterial activity, immunomodulation, promotes bone regeneration	[5c]
Chitosan	BPNS	PRP, methotrexate	In situ‐gelling	Photothermal activity, sustained release of methotrexate, promotes bone regeneration	[226]
Alginate	GO	Dexamethasone, Ca^2+^ (as cross‐linker)	Microgel	Efficient encapsulation and sustained release of dexamethasone, promotes bone regeneration.	[227]
Collagen	GO	EDS and NHS (as cross‐linkers), HAp	Microgel	Multi‐layer structure, uniform distribution of mineralized HAp, promotes bone regeneration.	[228]

The individual syntheses vary in the preparation process of the precursor solution, as well as of the 2D material, and in the mechanism employed to induce gelation. In terms of the interactions between polymer chains, hydrogels can be classified into chemical and physical hydrogels. Whereas polymer chains in chemical hydrogels are cross‐linked via covalent bonds, the assembly of physical hydrogels relies on non‐covalent interactions, such as hydrogen bonds and other Van der Waals forces. Simultaneously, 2D materials can be incorporated following different approaches, namely synthesizing a hydrogel from a suspension of the 2D material, physically incorporating the nanomaterial after gelation of the polymer, synthesizing the nanomaterial in situ after gelation (from precursors embedded in the hydrogel) or employing the nanomaterial as a cross‐linker to induce the formation of a hydrogel.

In the context of bulk hydrogels containing 2D nanosheets, particularly for regenerative and therapeutic purposes, the control of microstructural characteristics such as pore size and pore interconnectivity, as well as of the mechanical strength, are essential. These properties are vital for effective cell encapsulation, ensuring mechanical integrity, and enabling controlled release of therapeutic agents at the implantation site.

Even though the study of interactions at the molecular level between polymer chains and nanosheets in 2D‐materials‐based hydrogels is often overlooked, these interactions play a key role in improving the mechanical and optical properties of these materials. Nanomaterials incorporated in hydrogel matrices usually act as multifunctional cross‐linkers, where each polymer chain interacts with several nanoparticles, and each nanoparticle in turn binds to multiple polymer chains.^[^ ^216^ ^]^ Naturally, the affinity between the nanomaterial and the polymer chains is a significant factor on defining the certain properties of a hydrogel; however, even low interaction strengths can be significant due to the high number of interaction points between polymer chains and nanoparticles.^[^ ^216^ ^]^

Dannert et al.^[^ ^216^ ^]^ have reviewed the different mechanisms through which interactions between nanomaterials and polymers at the molecular level can affect the macroscopic properties of nanocomposite hydrogels. Briefly, the presence of nanoparticles can improve a hydrogel's resistance to mechanical stress, by dissipating part of the energy via the desorption of polymer chains from nanoparticles. Since this desorption is reversible, detached chains can further reattach to nanoparticles, thus avoiding permanent damage to the structure of the hydrogel. It is also through this mechanism that many nanocomposite hydrogels exhibit self‐healing behavior.^[^ ^217^ ^]^ Furthermore, the ability to form reversible physical interactions often provides these materials with adhesive properties. Beyond the improved mechanical properties, nanocomposite hydrogels sometimes exhibit improved optical properties, due to the additional cross‐linking leading to smaller, more uniformly sized pores, thus improving the transparency of the material. Additionally, as mentioned before, the incorporation of nanoparticles can introduce additional functionality such as light responsiveness into these systems, which can be very advantageous for the regenerative and therapeutic purposes discussed in this review.

**Figure 7:**
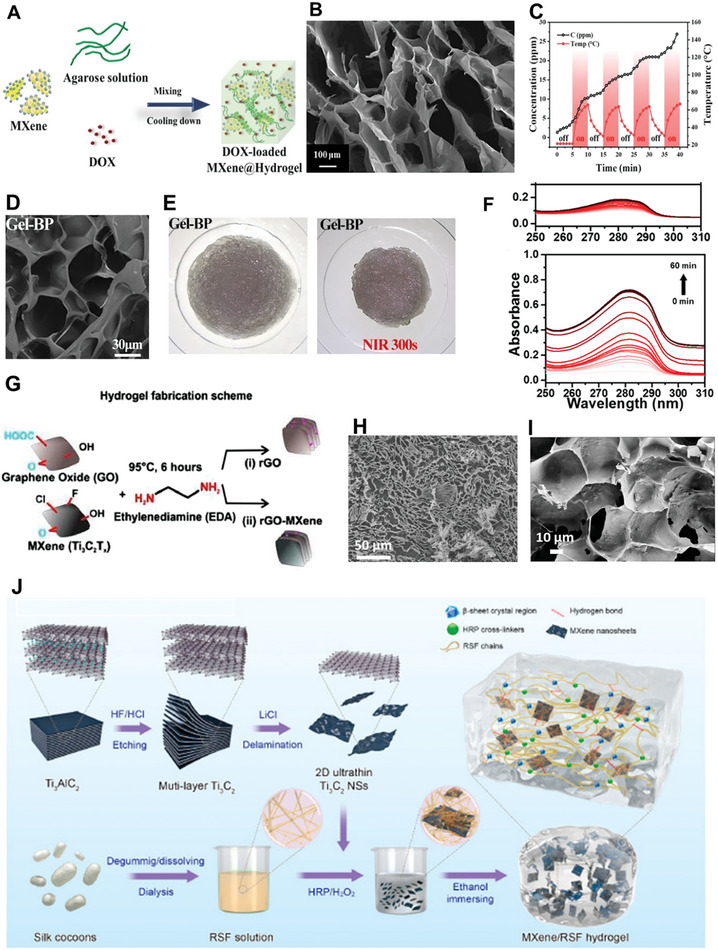
Selected examples of recently published 2D materials‐based bulk hydrogels with relevance for the treatment of bone diseases and defects. A–C) Synthesis, SEM image, and photothermal response of the doxorubicin‐loaded hydrogel developed by Dong et al.^[^ ^215b^ ^]^ Reproduced with permission.^[^ ^215b^ ^]^ Copyright, Elsevier. D,E) SEM image and photothermally induced shrinking of the hydrogel developed by Xu et al.^[^ ^5d^ ^]^ Reproduced with permission.^[^ ^5d^ ^]^ Copyright, Royal Society of Chemistry. F) Release of emetine with (bottom) and without (top) NIR irradiation from the hydrogel developed by Xie et al.^[^ ^215a^ ^]^ Reproduced with permission.^[^ ^215a^ ^]^ Copyright 2020, Wiley‐VCH. G,H) Synthesis and SEM image of the hydrogel developed by Wychowaniec et al.^[^ ^219^ ^]^ Reproduced with permission.^[^ ^219^ ^]^ Copyright 2020, Elsevier. I) SEM image of the hydrogel developed by Wang et al.^[^ ^215c^ ^]^ Reproduced with permission.^[^ ^215c^ ^]^ Copyright 2023, MDPI. J) Synthesis of the hydrogel developed by Hu et al.^[^ ^5e^ ^]^ Reproduced with permission.^[^ ^5e^ ^]^ Copyright 2022, Elsevier.

Scheme 1 shows a diagram of the main relevant properties through which 2D material‐based hydrogels can aid in the treatment of bone diseases and defects. The most studied therapeutic application is the controlled and localized release of drugs. For this purpose, drugs are preloaded onto the 2D material‐based bulk hydrogel, which is then implanted into the patient. The drugs are released locally as a response to an external or internal stimulus, frequently the irradiation with NIR radiation.^[^ ^218^ ^]^ Dong et al.^[^ ^215b^ ^]^ developed a doxorubicin‐loaded smart agarose hydrogel containing photothermally active Ti_3_C_2_T_x_ MXene nanosheets, by adding the anticancer drug doxorubicin and MXene nanosheets to an aqueous solution of low‐melting‐point agarose at 60 °C (**Figure** **7**A). Reversible physical gelation was achieved by allowing the mixture to cool down to room temperature. Cross section SEM showed a network of interconnected pores (Figure 7B), and EDS mapping confirmed the uniform distribution of the MXene nanosheets within the hydrogel. The rheological properties of the hydrogel were found to decline at temperatures above 60 °C, indicating the melting of agarose. The presence of MXene nanosheets had no significant influence on the melting temperature of the hydrogel. It did, however, provide photothermal activity to the system: irradiation with NIR radiation led to reversible temperature increases, which were enough to melt the hydrogel and induce the release of the preloaded doxorubicin (Figure 7C). The decrease of the temperature upon ending the irradiation caused the agarose to return to the gel state, effectively interrupting the drug release.

Recently, Xu et al.^[^ ^5d^ ^]^ synthesized a poly(N‐isopropylacrylamide) hydrogel containing BPNS by dissolving the polymer precursors and a photoinitiator in water, followed by the dispersion of BPNS in the precursor solution. Chemical gelation was achieved through the formation of covalent bonds by photopolymerization, and the hydrogel was lyophilized and immersed in a solution of mesenchymal stem cell derived exosomes. SEM images showed a porous network, with thicker walls and more regular pores in the presence of BPNS (Figure 7D). The evaluation of the swelling ratio indicated an excellent water absorption capacity, suggesting the possibility of high efficiency loading of exosomes. Due to the presence of photothermally active BPNS, irradiation with NIR led to temperature increases up to ≈45 °C. These temperature changes were reproducible along several irradiation cycles, confirming the thermal stability of the obtained material. Additionally, it was found that the fabricated hydrogel contracted when irradiated with NIR, causing a significant amount of water to be expelled from the hydrogel, carrying with it the preloaded exosomes (Figure 7E).

The ability of 2D materials‐based hydrogels for controlled drug release is frequently synergistically coupled with photothermal therapy to improve the hydrogels’ ability to treat tumors. In these cases, irradiation with NIR laser produces an increase in the local temperature, which helps kill cancerous cells and simultaneously induces the release of an anticancer drug.^[^ ^229^ ^]^ Bufalin is an anticancer drug that can treat various kinds of tumors, but it exhibits myocardial toxicity, which greatly limits its applications.^[^ ^230^ ^]^ To make use of the beneficial properties of this drug, while simultaneously minimizing its negative side effects, He et al.^[^ ^215e^ ^]^ obtained a photothermally active polypeptide‐based hydrogel containing BPNS, which was loaded with bufalin. The synthesis process was simple: a synthetic polypeptide (Nap‐GFFYGRGD) was dissolved in phosphate buffered saline (PBS) buffer by heating to ≈90 °C, the pH was adjusted to physiological values and a solution of bufalin was added. Finally, BPNS were suspended in the precursor solution, and physical gelation occurred when the mixture was allowed to cool down to room temperature. Observation with TEM showed a porous 3D network of intertwining nanofibers. The rheological properties were evaluated by doing a dynamic frequency sweep at room temperature. An increase in the value of G’ for increasing frequencies indicated good ductility and resistance to large shear forces, suggesting the obtained hydrogel is injectable. The presence of BPNS provided the hydrogel with photothermal properties: NIR irradiation led to temperature increases of biological relevance for cancer treatment, with a photothermal conversion efficiency of 27.4%. These temperature increases were found to be reversible and reproducible, and to cause the melting of the hydrogel matrix, inducing the fast release of bufalin. What's more, the drug release rate was observed to be dependent on the concentration of BPNS.

In another study, an agarose hydrogel containing PEG‐modified BPNS and emetine was developed by dissolving low‐melting‐point agarose in water at 60 °C, adding a dispersion of BP@PEG and emetine, then cooling down to 4 °C to induce physical gelation.^[^ ^215a^ ^]^ The obtained hydrogel was shown to be biocompatible, and the presence of BP@PEG imparted photothermal properties to it. The temperature increase produced by irradiation with NIR was shown to be reproducible over multiple cycles. The loading of the hydrogel with emetine, a stress granule inhibitor that can sensitize tumors to photothermal therapy, did not affect the photothermal behavior of the developed material. Moreover, the release of this drug was found to be slow under no irradiation, but could be accelerated significantly through irradiation with NIR, in a time‐dependent manner (Figure 7F).

Beyond the well‐known photothermal and controlled drug release properties that can be imparted to 2D materials‐based hydrogels, much work has been done on improving other properties of relevance for the treatment of bone diseases and defects. For instance, materials for use in medical implants for load‐bearing tissues need to be mechanically tough.^[^ ^231^ ^]^ One option for improving the mechanical properties of hydrogels is the generation of double networks to chemically reinforce the structure of the hydrogel. In this regard, Basu et al.^[^ ^215d^ ^]^ developed a mechanically reinforced DNA hydrogel containing 2D alginate‐functionalized WS_2_ nanosheets. For this purpose, DNA was dissolved in water, and WS_2_‐alginate nanosheets were dispersed in the solution. PEG diepoxide was added to act as a cross‐linker, and tetramethyl ethylenediamine (TEMED) was added due to its ability to induce DNA denaturation, initiating the gelation process. This mixture was heated to complete the gelation, and the obtained hydrogel was soaked in a CaCl_2_ solution to generate a second, ionically cross‐linked network. Observation of the hydrogel morphology with SEM showed a porous network, with a higher cross‐linking density after the addition of Ca^2+^ ions. The presence of WS_2_ nanosheets improved the mechanical properties of the material. Similarly, the formation of ionic bonds between alginate groups and Ca^2+^ ions further improved the mechanical properties of the hydrogel, making it a promising biomedical scaffold material.

Another key property of materials for use in biomedical implants is their porosity and surface roughness, which enable the adhesion, penetration and proliferation of cells inside the scaffold.^[^ ^231^ ^]^ This is a key feature for materials aimed at enhancing tissue regeneration, such as bone healing. Wychowaniec et al.^[^ ^219^ ^]^ developed a self‐standing, 2D materials‐based hydrogel with interconnected pores and surface chemistry appropriate for tissue regeneration applications. This hydrogel was synthesized by suspending monolayer rGO and Ti_3_C_2_T_x_ MXene nanosheets in water and adding ethylenediamine to act as a cross‐linker. The mixture was heated solvothermally to yield the final bulk hydrogel (Figure 7G). SEM images showed a well‐interconnected network of nanoflakes with large nanopores (Figure 7H). The size of the pores was determined to be 7 ± 5 µm, slightly larger than the value for the analogous, rGO‐only hydrogel (6 ± 4 µm). While long, accordion‐like stacks of nanosheets were observed for the latter, which contributed to its elasticity, these were significantly less frequent in the rGO‐MXene hydrogel, indicating the successful and uniform integration of both 2D materials into a single network. Lastly, the storage moduli of both materials were determined: even though the rGO‐only hydrogel yielded higher values, indicating superior mechanical properties, the rGO‐MXene hydrogel showed less deterioration of these properties after repeating the measurements. This was attributed to the higher hydrophilicity of MXene, which favored the recovery of the original hydrated structure after shearing.

A further key property of biomaterials for bone regeneration is their electrical conductivity. Since bone regeneration is greatly affected by the electrical microenvironment of the extracellular matrix (ECM), external electrical stimulation has been extensively studied to enhance this process.^[^ ^232^ ^]^ With this in mind, the electrical conductivity of materials used in biomedical implants is critical. The inclusion of conductive 2D materials into hydrogels can significantly improve their electrical conductivity, thus enhancing their bone regeneration potential. For example, Hu et al.^[^ ^5e^ ^]^ recently dispersed silk‐fibroin‐bound Ti_3_C_2_ MXene nanosheets in a silk fibroin solution containing horseradish peroxidase, and induced the gelation of the system by adding hydrogen peroxide. The obtained hydrogel was subsequently soaked in alcohol to improve its mechanical properties, resulting in a dual‐cross‐linked physical hydrogel (Figure 7J). TEM showed densely packed silk fibroin nanofibrils bound to the edges of MXene nanosheets. The uniform distribution of MXene nanosheets within the hydrogel was confirmed with EDS mapping. Furthermore, it was found that the presence of MXene nanosheets accelerated the gelation process, and that encapsulation in silk fibroin probably slows down the oxidation of MXene. The internal and surface morphology of the hydrogel were evaluated with SEM: the hydrogel possessed a porous internal morphology, with increasingly rougher surfaces for increasing amounts of MXene. Although the incorporation of MXene lowered the ductility of the hydrogel, it had no significant effect on its mechanical strength. More importantly, the MXene imparted an electrical conductivity of 4 × 10^−4^ S cm^−1^ to the system, which could fully and instantaneously be recovered after cutting and rejoining the hydrogel. Interestingly, it was found that the hydrogel could act as a sensor, detecting small human motions such as the bending of a finger.

Ultimately, the versatility and variability of 2D materials‐based bulk hydrogels goes well beyond what has been explored so far. The ability to functionalize both the 2D material and the polymer chains allows for a very wide range of modifications to tune the final properties of these hydrogels for targeted application. Additionally, the combination of the different approaches and special properties mentioned above, as well as the inclusion of additional components into the system, leaves ample room for further research and significant advances in this field. For instance, very recently Wang et al.^[^ ^215c^ ^]^ developed a GO‐containing maleilated‐chitosan hydrogel which was functionalized with zwitterionic L‐cysteine to take advantage of the excellent antimicrobial and non‐cytotoxic properties of zwitterionic materials. Furthermore, β‐tricalcium phosphate (β‐TCP) nanoparticles were included to aid the biomineralization process of bone. The synthesis process was as follows: maleic anhydride and chitosan were dissolved in DMSO, and a suspension of β‐TCP nanoparticles and GO was added. The mixture was ultrasonicated, dried and washed with water. The zwitterionic groups were finally incorporated by immersing the obtained hydrogel in a solution of L‐cysteine. Chemical cross‐linking was incorporated via click reactions between thiol groups of L‐cysteine residues and double bonds provided by maleic anhydride. SEM showed a honeycomb‐like structure with uniformly sized pores (Figure 7I). Increasing amounts of GO led to higher surface roughness and pore interconnectivity. The porosity was determined to be in the range of 57–72%, and to increase for increasing GO concentrations. In contrast, the swelling ratios, which indicated excellent water absorption ability, were found to decrease for increasing GO concentrations. This was attributed to the hydrophobic GO reducing the overall hydrophilicity of the resulting hydrogel. Furthermore, the incorporation of GO enhanced the mechanical properties of the material, although only up to a certain concentration. Beyond 1 mg mL^−1^, the mechanical properties worsened due to restacking and agglomeration of GO nanosheets. Finally, the presence of GO was found to significantly slow down the degradation of the hydrogel.

### 3.2 Hydrogels with Complex Architectures – Gradient Multi‐Layer, 3D‐Printed, and Scaffold‐Supported Hydrogels

The scope of applications of 2D materials‐based hydrogels can be expanded even further by developing materials with more complex architectures. These special architectures, which support or even enhance the functionality of these materials, can be achieved through techniques as simple as combining several bulk hydrogels to create multilayer systems,^[^ ^220^ ^]^ or as advanced and customizable hydrogel using 3D printing.^[^ ^14^, ^221^ ^]^

In recent years, hydrogels with special architectures have been particularly studied in the field of bone regeneration, with the aim of utilizing the flexibility in hydrogel architectures to improve the materials’ potential in this area.^[^ ^14^, ^220^, ^221^, ^222^ ^]^ Recently, Xu et al.^[^ ^220^ ^]^ developed a 2D‐material‐based hydrogel with a simple, bilayer structure that mimicked the periosteum region of bone. This material was obtained by stacking two bulk hydrogels: a lower one, obtained by adding β‐TCP and polyethylene glycol diacrylate (PEGDA) to an aqueous solution of gelatin methacryloyl (GelMA) and inducing chemical gelation via irradiation for 5 min at 365 nm; and an upper one, prepared by dispersing BP@Mg nanosheets in a solution of GelMA and inducing gelation as before. While the lower layer's composition was aimed at producing a tough double network hydrogel with excellent bone regeneration properties, the upper layer was designed to mimic the periosteum, and promote angio‐ and neurogenesis. SEM showed the highly porous structure of both layers, with pores larger than 100 µm. The upper layer was found to possess larger pores and higher water absorption capacity, indicated by the higher swelling ratio. Furthermore, the lower layer had a higher elastic modulus, likely due to the presence of the interpenetrating double network.

Despite the simplicity of combining several bulk hydrogels to achieve the desired architecture, 3D printing is the most common technique for achieving complex architectures in hydrogels.^[^ ^14^, ^221^, ^222^ ^]^ Its main advantage is that it allows for fully customized, complex shapes that can be adjusted to each particular use.^[^ ^233^ ^]^ 3D‐printed hydrogels can, for instance, be designed to accurately fill bone defects with irregular shapes,^[^ ^234^ ^]^ or to have grid‐like structures that optimize cell seeding and proliferation.^[^ ^234b^ ^]^ Taking advantage of this technology, Nie et al.^[^ ^14a^ ^]^ recently designed a 2D‐material‐based hydrogel aimed at healing irregularly‐shaped infected bone defects. For this purpose, a bioink was prepared by dissolving GelMA and a photoinitiator in PBS buffer, then adding β‐TCP, alginate and Ti_3_C_2_ MXene nanosheets. The ink was kept at 4 °C until use, at which point it was heated to 20 °C and 3D‐printed onto a cooling plate, which had been precooled to 4 °C, in a grid‐like pattern. Initial chemical cross‐linking on the cooling plate was achieved by irradiation for 15 s with 405 nm light, which was then followed by final physical cross‐linking via soaking of the printed material in a SrCl_2_ solution. The developed bioink had appropriate rheological properties for extrusion 3D‐printing, including good temperature sensitivity and shear thinning behavior, the latter shown by the decreasing viscosity for increasing shear rates. The 3D‐printed hydrogel possessed a grid‐like architecture, with a regular spacing of 1 mm. SEM of the internal region showed a highly porous network. EDS mapping revealed that both the MXene nanosheets and the Sr^2+^ ions were uniformly distributed in the hydrogel. The incorporation of MXene nanosheets, as well as alginate‐Sr^2+^ cross‐linking, was observed to improve the mechanical strength of the material. The hydrogel had a swelling ratio close to 1000%, and an absorption maximum near 800 nm, promising for photothermal therapy.

Similarly, earlier this year Zhang et al.^[^ ^221^ ^]^ reported the synthesis of a 3D‐printed rGO‐based hydrogel with neuralized bone regeneration properties. A bioink was prepared by dissolving GelMA and a photoinitiator in PBS buffer at 50 °C, and dispersing rGO nanosheets in the solution. The hydrogel was then 3D‐printed at 25 °C onto a precooled cooling plate in a grid‐like pattern, and chemically cross‐linked with 405 nm radiation for 1 min. The prepared bioink showed excellent shear thinning properties and good temperature sensitivity, as well as an appropriate swelling ratio, making it suitable for extrusion 3D‐printing. SEM images of the 3D‐printed hydrogel showed a regular grid architecture with 1 mm spacing, and pore sizes that decreased with increasing rGO concentrations. The mechanical properties were optimal for an rGO concentration of 0.05% and worsened for higher concentrations due to incomplete inner photo‐cross‐linking caused by the rGO shielding the curing light.

Despite the simplicity of combining multiple bulk hydrogels and the versatility of 3D printing, research on 2D materials‐based hydrogels with special architectures goes beyond these two aforementioned techniques. Specially in order to fabricate more complex systems with improved functionalities, some studies integrate more than one technique. For example, Miao et al.^[^ ^222^ ^]^ developed a gel‐scaffold construct that promotes vascularized bone regeneration by combining a 3D‐printed scaffold with a shear‐thinning hydrogel filling. The scaffold was obtained by 3D printing polycaprolactone (PCL) into a 3D grid. Meanwhile, the hydrogel precursor was prepared through thermal processing and incorporation of VEGF‐loaded BPNs. After the hydrogel precursor was allowed to cool down to room temperature, which caused the gelation of the system due to physical cross‐linking, it was injected into the 3D‐printed scaffold at 40 °C and allowed to anneal at room temperature. In this system, the PCL scaffold provided structural support to the system by improving its mechanical strength, while the hydrogel provided functionality by mimicking the dynamic microenvironment of the extracellular matrix. SEM of the DNA hydrogel showed a network of interconnected pores, with smaller pore sizes in the presence of BPNS. No significant swelling of this hydrogel in PBS was observed, and shear‐thinning behavior was evidenced by a decreasing viscosity for increasing shear rates. The incorporation of BPNS was found to significantly increase the hydrogel's storage modulus, and the hydrogel was able to withstand cyclic strain, rapidly recovering its storage modulus after each cycle. The 3D‐printed scaffold consisted of a regular grid with ≈500 µm sized pores, with the DNA hydrogel homogenously filling the empty spaces. The resulting construct could release the loaded VEGF, with the BPNS slowing down this release due to a tighter cross‐linking network as well as direct interactions with the VEGF. Overall, this construct can be viewed as proof of concept of a way to overcome the intrinsic mechanical weakness of hydrogels by incorporating mechanically tough scaffolds.

### 3.3 Hydrogel Coatings

Hydrogel coatings are hydrogel layers that can be placed on the surface of a substrate to impart special properties to said surface. This can be particularly useful for diseases and defects affecting load‐bearing bones, where the mechanical properties achievable in simple hydrogel systems are not sufficient. In those cases, mechanically tough inert materials can be used as implants, and bioactive hydrogel coatings can be applied to the surface of these implants to incorporate additional properties and functionalities, such as osteogenesis enhancement, anticancer and antibacterial properties.^[^ ^9^, ^223^, ^235^ ^]^

**Figure 8:**
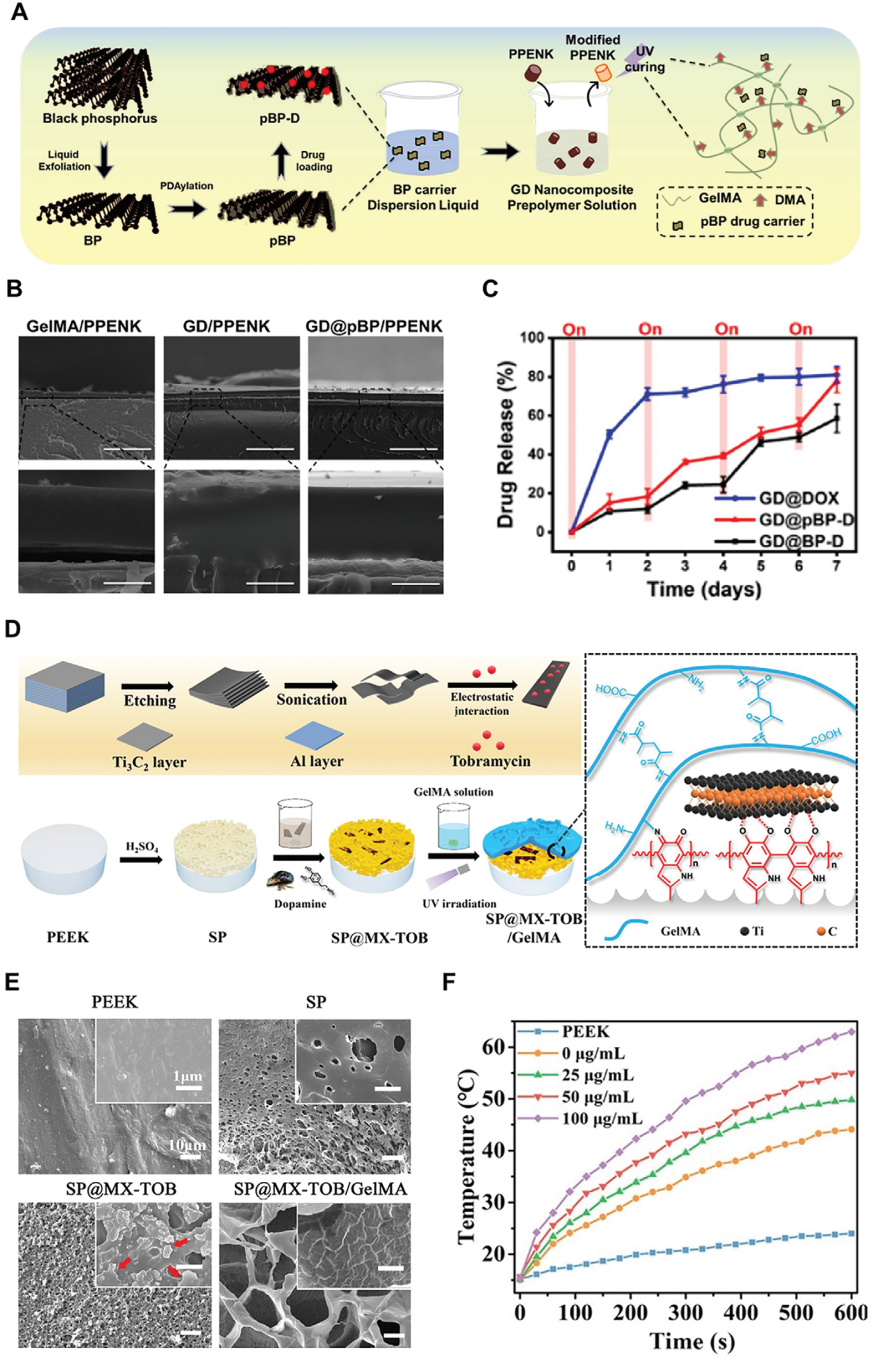
Selected examples of recently published 2D materials‐based coating hydrogels with relevance for the treatment of bone diseases and defects. A–C) Synthesis, SEM image, and doxorubicin release profile under repeated irradiation with 808 nm light of the hydrogel coatings developed by Li et al.^[^ ^9b^ ^]^ (GD: GelMA‐DMA, pBP: polydopamine‐modified BPNS). Reproduced with permission.^[^ ^9b^ ^]^ Copyright 2023, Elsevier. D–F) Schematic representation of the synthesis and composition, SEM images, and photothermal behavior of the hydrogel coating developed by Yin et al.^[^ ^223^ ^]^ Reproduced with permission.^[^ ^223^ ^]^ Copyright 2020, American Chemical Society.

One particular application for which 2D materials‐based hydrogel coatings have been researched is the post‐operative care of bone tumor resections.^[^ ^9^, ^223^ ^]^ With this in mind, Li et al.^[^ ^9b^ ^]^ developed a doxorubicin‐hydrochloride‐loaded GelMA and dopamine methacrylate (DMA) hydrogel layer containing BPNS functionalized with polydopamine (PDA), as a coating for a poly(phthalazinone ether nitrile ketone) (PPENK) implant. In short, BPNS were modified with PDA, loaded with doxorubicin hydrochloride, and added to a solution of GelMA and DMA. The resulting suspension was spin‐coated onto the surface of a PPENK substrate, and covalently photo‐cross‐linked with UV radiation (**Figure** **8**A). The resulting hydrogel had reasonable stability, with close to 60% of the initial mass remaining after 2 weeks. Cross sectional SEM showed a coating thickness of ≈30 µm (Figure 8B). Unlike for the GelMA hydrogel coating, all hydrogel coatings containing both GelMA and DMA presented no gap between the hydrogel and the PPENK substrate, suggesting that DMA improves the adhesion properties of the coating material. This was further confirmed by a 90° peel test, where coatings containing DMA showed significantly higher adhesion energies than the one without DMA. Hydrogel coatings without BPNS showed an initial burst release of doxorubicin, which was not controllable with NIR light. In contrast, samples containing BPNS were capable of NIR‐controlled doxorubicin release (Figure 8C).

In a similar study, Yin et al.^[^ ^223^ ^]^ obtained a GelMA hydrogel coating containing tobramycin‐loaded Ti_3_C_2_ MXene nanosheets, as a coating on a bioinert sulfonated PEEK (SP) substrate. Briefly, PEEK was immersed in concentrated sulfuric acid for 6 min and then rinsed with acetone and water, to produce a porous SP substrate. Next, Ti_3_C_2_ MXene nanosheets were loaded with the antibiotic tobramycin and added to a PDA solution, in which the SP substrate was immersed for one day at 37 °C. Thereafter, the SP substrate was washed with water to remove unattached nanosheets and soaked in a GelMA solution containing a photoinitiator. Finally, the soaked substrate was irradiated with 365 nm radiation during 15 min to induce a chemical gelation through the formation of new covalent bonds (Figure 8D). SEM showed the smooth surface of the PEEK, which became porous after exposure to sulfuric acid (Figure 8E). PDA and tobramycin‐loaded MXene nanosheets were observed to be evenly distributed on the surface of the SP substrate, and the formation of a 3D porous network was visible upon addition of the GelMA hydrogel layer. Additionally, PDA and tobramycin‐loaded MXene nanosheets were found to provide photothermal properties to the coating, with the achieved temperature increasing with the concentration of MXene (Figure 8F).

### 3.4 In Situ‐Gelling Hydrogels

Bone diseases such as bone cancer and osteomyelitis can cause severe lesions that leave behind irregularly shaped bone defects after surgical intervention.^[^ ^234^, ^236^ ^]^ The complex geometry of these lesions can complicate the design of medical implants that properly fit the defects. In some cases, technologies as advanced as 3D printing are not enough to obtain implants that completely and perfectly fill complex bone defects. In situ‐gelling hydrogels are a potential solution for this problem, since they can be injected into the defect, where they can adopt the irregular shape of the cavity before gelation takes place in situ.^[^ ^237^ ^]^ Beyond their ability to adapt to any shape, they also possess the significant advantage of not requiring surgical intervention to be implanted: they can be directly injected into the defect in a minimally invasive procedure.^[^ ^237^ ^]^

**Figure 9:**
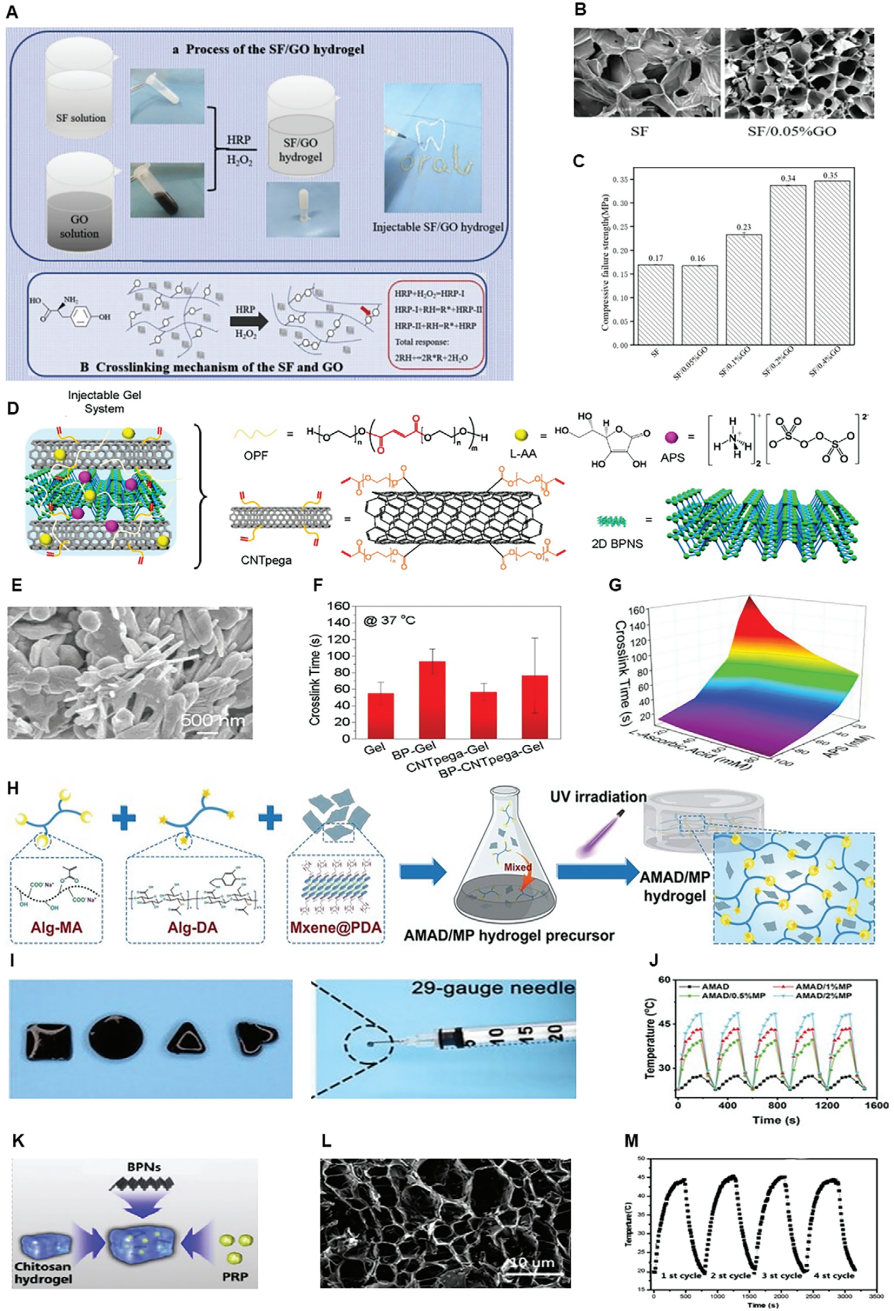
Selected examples of recently published 2D materials‐based in situ‐gelling hydrogels with relevance for the treatment of bone diseases and defects. A–C) Synthesis, SEM images, and stress at fracture of the hydrogels developed by Wang et al.^[^ ^224^ ^]^ Reproduced with permission.^[^ ^224^ ^]^ Copyright 2002, Elsevier. D–G) Schematic representation of the composition, internal SEM image, and cross‐linking times of the hydrogels developed by Liu et al.^[^ ^225^ ^]^ Reproduced with permission.^[^ ^225^ ^]^ Copyright 2020, American Chemical Society. H–J) Synthesis, injectability and complex shapes, and photothermal properties of the hydrogel developed by Wu et al.^[^ ^5c^ ^]^ Reproduced with permission.^[^ ^5c^ ^]^ Copyright 2023, Wiley‐VCH. K–M) Composition, SEM image, and photothermal properties of the hydrogel developed by Pan et al.^[^ ^226^ ^]^ Reproduced with permission.^[^ ^226^ ^]^ Copyright 2020, Elsevier.

There are two main approaches to the synthesis of in situ‐gelling hydrogels. In both cases, a precursor solution is prepared that is injected into the bone defect in its liquid state, before gelation occurs. In a first approach, all components necessary for spontaneous hydrogel formation are combined before injection, including cross‐linking initiators.^[^ ^237b^ ^]^ In this approach, the gelation time is optimized to allow enough time to inject the solution into the patient, without allowing for significant spreading of the liquid away from the lesion site before spontaneous gelation occurs. Recently, Wang et al.^[^ ^224^ ^]^ developed an injectable GO‐containing silk fibroin hydrogel loaded with bone marrow mesenchymal stem cells for bone healing applications. This hydrogel was prepared by dispersing GO nanosheets in water, adjusting the pH to 10 with NaOH, and adding a silk fibroin solution. Gelation was induced by adding horseradish peroxidase and hydrogen peroxide, which cause tyrosine residues to cross‐link (**Figure** **9**A). The gelation time of this system was found to increase for increasing concentrations of GO, with 15 min for the hydrogel without GO and 24 min for the hydrogel with the highest concentration (0.4% GO). SEM showed a 3D network with well interconnected pores (Figure 9B). The pore sizes ranged from 100 to 300 µm, with higher concentrations of GO leading to slightly smaller pores. No agglomeration of GO nanosheets was observed in any of the samples. All tested concentrations of GO yielded hydrogels with porosities above 90%. The mechanical properties of the final hydrogels improved for increasing concentrations of GO (Figure 9C), probably due to the higher degree of cross‐linking and the smaller pore sizes.

In the same year, Liu et al.^[^ ^225^ ^]^ designed an electrically conductive, mechanically strong and injectable hydrogel containing BPNS and carbon nanotubes (CNT) for minimally invasive bone repair (Figure 9D). For this purpose, polyethylene‐glycol‐acrylate‐functionalized CNT (CNTpega) and BPNS were dispersed in water, to which biodegradable oligo(polyethylene glycol fumarate) (OPF) and PEGDA were added. To induce gelation, ammonium persulfate and L‐ascorbic acid were added as cross‐linking initiator and accelerator, respectively. Figure 9G shows the effect of the concentration of L‐ascorbic acid and ammonium persulfate on the gelation time of the system. Briefly, increasing concentrations of both lead to shorter gelation times. The effect of the incorporation of BPNS and CNTpega was also evaluated and is shown in Figure 9F. The final gelation time of the optimized hydrogels was in the 1–2 min range. SEM showed a homogeneous distribution of CNTpega and BPNS within the hydrogel (Figure 9E). The hydrogels exhibited ten‐fold swelling, and a mechanical strength that was improved by the addition of CNTpega. Increasing concentrations of CNTpega were found to increase the conductivity of the hydrogel, whereas the concentration of BPNS had no significant influence on their conductivity. Finally, due to the incorporation of BPNS the hydrogel was capable of releasing phosphate ions, with an initial burst release during the first week, and slower release in the following days.

A second approach involves designing a hydrogel system where gelation is induced by an external stimulus, such as light irradiation.^[^ ^237b^ ^]^ In this case, a precursor solution is prepared that either does not contain any initiator or contains an initiator that needs an external stimulus to be activated, so that gelation cannot occur without prior stimulation. This removes the strict time constraint for immediate injection into the patient, as gelation is actively induced when needed. Earlier this year, Wu *et al.* ^[^ ^5c^ ^]^ obtained an injectable and photocurable methacrylated alginate (Alg‐MA) and dopamine‐grafted alginate (Alg‐DA) hydrogel containing PDA‐modified Ti_3_C_2_ MXene, which could assist in the repair process of irregularly shaped bone defects. A precursor suspension for this material was synthesized by preparing a solution of Alg‐MA, Alg‐DA, and a photoinitiator in PBS buffer, to which a dispersion of PDA‐modified Ti_3_C_2_ MXene in PBS buffer was added (Figure 9H). To induce chemical gelation, this system was irradiated with 405 nm radiation for 3 min. The obtained hydrogel precursor could be injected through differently sized syringe needles without causing blockages and could take the shape of the mold before gelation, yielding hydrogels with complex shapes (Figure 9I). The incorporation of MXene@PDA did not have any adverse effects on the gelation ability of the hydrogel. SEM images showed a homogenous distribution of MXene@PDA in the hydrogels, with some agglomeration for the highest concentration (2% MXene@PDA). All hydrogels exhibited highly interconnected porous structures, with pore sizes ranging from 150 to 250 µm. The incorporation of MXene@PDA was observed to increase the roughness of the originally smooth pore walls. As for the mechanical performance, MXene@PDA improved the mechanical strength of the hydrogel up to a concentration of 1%, with higher concentrations adversely affecting the mechanical properties due to the aggregation of MXene nanosheets. Furthermore, the developed hydrogel was capable of effectively recovering its original shape after repeated cycles of compression. Lastly, the incorporation of MXene@PDA imparted photothermal properties to the final hydrogel, which remained stable over five cycles of consecutive NIR irradiation (Figure 9J).

Following the same approach, Pan et al.^[^ ^226^ ^]^ developed a BPNS‐containing injectable hydrogel for the treatment of bone loss and inflammation caused by rheumatoid arthritis. In this study, deacetylated chitosan was dissolved in an acetic acid solution, a suspension of BPNS was added and the pH was adjusted to 7.2 using sodium β‐glycerophosphate. In parallel, platelet‐rich plasma (PRP) was activated by adding CaCl_2_. The formed PRP gel was freeze‐dried and ground to a fine powder, which was finally mixed into the previously prepared mixture. The gelation of the obtained precursor could be induced by a temperature increase, for instance via irradiation with NIR light. Figure 9K shows a schematic representation of the composition of the developed hydrogel. SEM showed an irregular porous structure (Figure 9L), and elemental mapping confirmed the homogenous distribution of BPNS within the hydrogel. Due to the presence of BPNS, the hydrogel was photothermally active, with a photothermal conversion efficiency of 43.19%. The power density of irradiation was optimized to 1.0 W cm^−2^, and the photothermal performance was shown to be stable over four irradiation cycles (Figure 9M).

### 3.5 Hydrogel Microparticles

Despite the clear advantages of in situ‐gelling hydrogels, developing the right chemistry for in situ gelation can be challenging. In these cases, hydrogel microparticles can provide an alternative for the repair of irregularly shaped bone defects. These microgels consist of hydrogel microparticles, which can be implanted into bone defects and adapt to their complex geometries, filling the cavity completely without the need for in situ gelation, and with a lower risk of diffusing away from the target site.^[^ ^238^ ^]^

Hydrogel microparticles can be synthesized in many varied ways, from simply dripping a hydrogel precursor into a cross‐linking solution, to using advanced techniques and tools such as microfluidics and micro‐stencil arrays.^[^ ^238^ ^]^ In a very simple process, G.V et al.^[^ ^227^ ^]^ produced dexamethasone‐loaded GO‐containing alginate hydrogel microspheres for bone regeneration. Briefly, an aqueous dispersion of GO and a solution of dexamethasone in ethanol were added to a solution of alginate. The resulting suspension was slowly dripped onto a CaCl_2_ solution to induce physical gelation. Stereomicroscopy showed spherical, 1.5 mm sized particles. The surface was smooth in the absence of GO but became wrinkled upon the incorporation of GO. The microgel had a porosity of 84%, which was higher than that of the microgel without dexamethasone, probably due to the cross‐linking of this drug to the other components of the hydrogel. Through SEM, cracks were visible on the surface, suggestive of a porous inner structure. Dexamethasone was efficiently encapsulated by these hydrogel microparticles, and a burst release was observed within the first hour, followed by a sustained release over 10 h.

**Figure 10:**
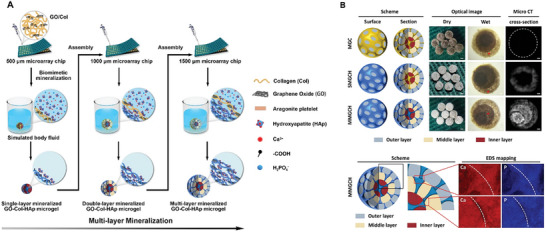
A) Synthesis process and B) internal and external structures of the 2D‐material‐based microgels developed by Zhou et al.^[^ ^228^ ^]^ MGC = multi‐layer GO‐Col microgel, SMGCH = single‐layer mineralized GO‐Col‐HAp microgel, MMGCH = multi‐layer mineralized GO‐Col‐HAp microgel. Reproduced with permission.^[^ ^228^ ^]^ Copyright 2022, Elsevier.

In contrast, Zhou et al.^[^ ^228^ ^]^ took advantage of more complex tools to prepare multi‐layer mineralized porous hydrogel microparticles inspired by the natural formation of pearls. For this purpose, a collagen solution containing GO nanosheets was uniformly applied onto a 500 µm micro‐stencil array chip and frozen at −20 °C. After lyophilization, the 500 µm particles were removed from the chip and immersed in ethanol, to which 1‐ethyl‐3‐(3‐dimethylaminopropyl)carbodiimide (EDC) and N‐hydroxysuccinimide (NHS) were added for chemical cross‐linking. After 24 h the hydrogel microparticles were washed and lyophilized, then soaked in a solution of CaCl_2_ and K_2_HPO_4_ to generate nuclei for biomineralization. Next, the microgel was immersed in simulated body fluids (SBF) for 7 days and lyophilized again. To form the second and third layers, the microparticles were successively added together with additional GO‐containing collagen solution to 1000 and 1500 µm micro‐stencil array chips, cross‐linking and biomineralizing after each step (**Figure** **10**A). Figure 10B shows the inner and outer structure of the microgel, both in the macro‐ and microscale. The so obtained mineralized multi‐layer microgel had spherical morphology, and a white surface due to the mineralized hydroxyapatite (HAp). The multi‐layer structure was visible under the microscope in the wet state, and micro‐CT showed the distribution of HAp through the samples: there was no HAp in the non‐mineralized microgel, a HAp shell comprising the outer layer of the single‐layer mineralized microgel, and full‐depth uniform distribution of HAp within the multi‐layer mineralized microgel. Regarding the inner structure, a multi‐layer porous structure was observed with SEM, with well‐connected 80–150 µm sized pores. SEM‐EDS further confirmed the uniform distribution of HAp throughout the entire multi‐layer mineralized microgel. All microgels had similar porosities (≈85%) and high swelling ratios, with the multi‐layer mineralized microgel having the highest swelling ratio of 575.63%. This microgel further exhibited high mechanical strength, and a storage modulus higher than its loss modulus, indicating a viscoelastic behavior.

## 4 Applications of 2D Materials‐Based Hydrogels for Bone Regeneration

Bone defects and fractures have traditionally been treated with bone grafting and metal protheses, which can be painful, costly, and prone to infection.^[^ ^239^ ^]^ These disadvantages have prompted the development of modern techniques such as bone tissue engineering (BTE), which involves using scaffolds, cells, and growth factors.^[^ ^240^ ^]^ An ideal scaffold should be biocompatible, non‐cytotoxic, and non‐immunogenic, with a highly porous 3D structure that can support cell and growth factor adhesion and transportation.^[^ ^241^ ^]^ One of the most promising scaffold materials are hydrogels, which have a 3D network structure and good biocompatibility, making them suitable for drug and cell delivery, tissue repair, and artificial organs. However, hydrogels have limitations, including poor mechanical strength and weak bio‐imaging capability, which can be overcome by combining them with other materials to create composite hydrogels.^[^ ^242^ ^]^ 2D nanomaterials, such as BP, offer a versatile raw material for bone regeneration. BP is a stable and non‐reactive form of phosphorus, an essential element for the human skeletal system. It can cause in situ phosphorus‐driven biomineralization, where the degradation product of BP, PO_4_^3−^, can coordinate with Ca^2+^ to promote bone formation.^[^ ^243^ ^]^ Mimicking this natural biomineralization process is essential for successful bone regeneration, especially in cranial defect repair.^[^ ^244^ ^]^ Therefore, incorporating 2D materials such as BP in BTE is a promising approach.

Researchers investigated the use of GO‐reinforced double network (DN) hydrogels for bone regeneration. Dithiothreitol‐modified graphene oxide (DGO)/aldehyde methylene sodium alginate (AMSA)/amino gelatin (AG) DN‐hydrogels showed improved osteogenic capacity compared to AMSA/AG hydrogels. They could serve as scaffolds for mechanical support and cell proliferation to promote bone regeneration.^[^ ^245^ ^]^ The limitations associated with conventional bone scaffolds, including issues of biocompatibility, mechanical strength, and the release of metal ions, create significant opportunities for exploring the application of composite hydrogels as cell carriers for scaffold synthesis. Hence, the combination of hydrogel scaffolds as cell carriers and of 2D nanomaterials for enhanced bioactivity in terms of cell proliferation and differentiation, has a broad prospect in the future of bone regeneration.^[^ ^246^ ^]^

### 4.1 Scaffold Design

2D nanomaterials incorporated hydrogels can be designed as macro or microscopic scaffolds that can be implanted in critical bone defect to support bone growth. These hydrogel nanocomposites can be classified as 1) bulk hydrogel scaffolds with enhanced mechanical strength and controlled microstructure and surface functionalities, which are often developed by conventional moulding or 3D/4D printing, 2) injectable and self‐healing bulk hydrogels, and 3) injectable microgel for cell delivery.^[^ ^247^ ^]^

In recent years, there has been an increasing demand for hydrogel scaffold that have a precisely defined 3D structure to align cells, create pathways for vascularization, or produce gradients that mimic natural tissue. One way to achieve this is through the use of pendant or backbone polymer groups that can be photo‐patterned. Another option is to utilize new 3D printing and stereo‐lithography technologies that can utilize cell‐containing or acellular “bio‐inks”. These systems require rapid gelation upon printing or extrusion to ensure spatial resolution, which is typically achieved through the mixing of monomers/polymers, nanofillers (e.g. 2D nanomaterial) with ions or photo‐initiators.^[^ ^248^ ^]^ The combination of biomaterials and cells in cell‐laden bioprinting offers a promising method for 3D cell culture that can more accurately simulate the natural cell growth process. 3D printing technology provides numerous benefits in creating multifunctional bone scaffolds that can mimic the complex structure of bone tissue. To achieve successful printing, inks or bioink (for cell‐laden gel) must have exceptional physicochemical and biological properties namely biocompatible to supports cell proliferation and growth, and functionality. For this purpose, the ideal hydrogels system should possess loosely cross‐linked network structure of hydrophilic polymers that can absorb large quantities of water without dissolving, the necessary porous and permeable characteristics to allow for cell migration, sufficient oxygen and nutrient diffusion, and closely mimic the microenvironment of cell growth.^[^ ^14a^ ^]^

Although bulk hydrogels is a simple method to achieve the scaffold architecture, 3D printing is the most common technique for creating complex hydrogel structures.^[^ ^14^, ^221^, ^222^ ^]^ 3D‐printed hydrogels can be customized to specific shapes and uses, such as filling bone defects with irregular shapes^[^ ^234a^ ^]^ or having grid‐like structures for optimized cell seeding and proliferation.^[^ ^234b^ ^]^

Hydrogels that can be injected offer numerous benefits for in vivo applications, such as filling up tissue defects without the need for invasive surgery.^[^ ^249^ ^]^ However, designing a polymer for an injectable hydrogel can be difficult too. One solution is to use a preformed hydrogel that can be injected and then solidified in situ,^[^ ^250^ ^]^ while another option is to prepare gel precursors that can undergo spontaneous or triggered gelation upon injection.^[^ ^251^ ^]^ In both cases, the polymer structure and cross‐linking chemistry must facilitate rapid gelation to avoid the leaching of soluble components into surrounding tissues.^[^ ^248^ ^]^ Injectable hydrogels can fill irregular tissue defects and promote in situ tissue regeneration, but directing stem cell differentiation in a 3D microenvironment for bone regeneration remains a challenge.

Cui et al.^[^ ^252^ ^]^ demonstrated the bone regeneration potential of chitosan‐montmorillonite hydrogels in promoting calvarial bone defect healing without the need for additional therapeutic agents or stem cells, by introducing 2D nanoclay particles with intercalation chemistry to chitosan gel network through in situ‐forming hydrogel. The presence of the nano‐silicates enhances the mechanical properties of the hydrogel, promoting the proliferation, attachment, and differentiation of encapsulated mesenchymal stem cells in vitro and in vivo.

**Figure 11:**
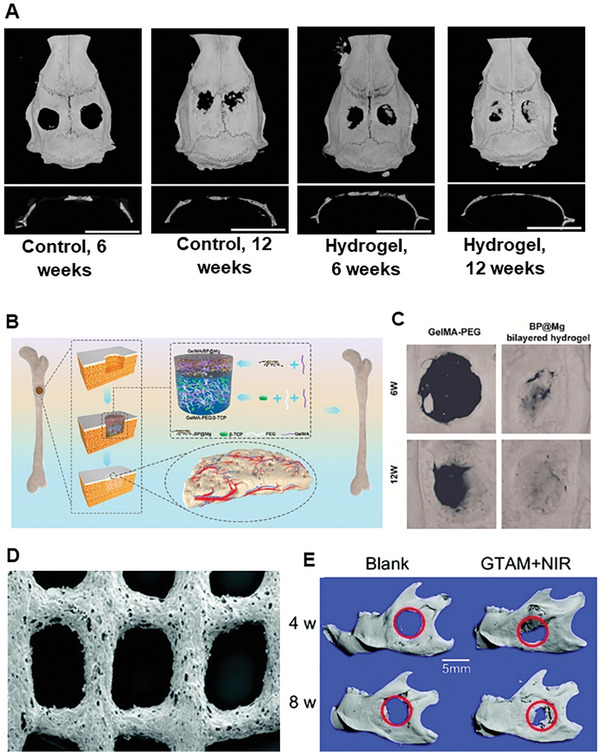
A) Micro‐CT scans of cranial bone defects on SD rat models. The effect of the zwitterionic hydrogel developed by Wang et al.^[^ ^254^ ^]^ on bone healing is shown. Reproduced with permission.^[^ ^254^ ^]^ Copyright 2023, MDPI. B) Diagrammatic representation of bilayer hydrogel developed by Xu et al.^[^ ^220^ ^]^ and C) its in vivo bone regeneration activity on calvarial defects. Reproduced with permission.^[^ ^220^ ^]^ Copyright 2022, Elsevier. D) SEM image of 3D‐printed hydrogel obtained by Nie et al.^[^ ^14a^ ^]^ and E) its in vivo bone regeneration activity on infected mandible defects. Reproduced with permission.^[^ ^14a^ ^]^ Copyright 2022, Royal Society of Chemistry.

While there have been limited reports on MXenes‐based injectable hydrogels, several studies have demonstrated their effectiveness in regenerating bone tissue. Yin et al.^[^ ^223^ ^]^ created a photothermally controlled hydrogel implant using MXene nanosheets, gelatin methacrylate (GM), and bioinert sulfonated polyetheretherketone. The implant promotes osteogenicity and can be used to treat osteosarcoma and bacterial infections. Ling et al.^[^ ^253^ ^]^ incorporated 2D Ti_3_C_2_ MXene nanosheets into a matrix of bacterial cellulose and silk fibroin to develop a hierarchically porous hydrogel scaffold. The resulting scaffold was bifunctional and showed promise in the clinical treatment of osteosarcoma, as it enhanced bone tumor ablation and repaired massive bone defects. Wang et al.^[^ ^254^ ^]^ developed a hydrogel containing GO and L‐cysteine, which showed excellent antimicrobial and non‐cytotoxic properties. Additionally, β‐tricalcium phosphate nanoparticles were added to aid the biomineralization process of bone, resulting in a biocompatible hydrogel that significantly accelerated bone regeneration in vivo (**Figure** **11**A). In another study, Xu et al.^[^ ^220^ ^]^ developed a 2D‐material‐based hydrogel that mimicked the periosteum region of bone. The hydrogel was made by stacking two bulk hydrogels, each with its specific properties. The lower layer was intended to produce a tough DN hydrogel with excellent bone regeneration properties, while the upper layer was designed to promote angio‐ and neurogenesis. This bilayer material achieved bone regeneration and simultaneously enhanced neurovascularization (Figure 11B,C).

Nie et al.^[^ ^14a^ ^]^ utilized 3D printing to create a composite hydrogel for healing *S. aureus*‐infected mandible defects of rats. The hydrogel was tested for antimicrobial and bone regeneration properties in rats. The scaffolds were implanted post‐surgery and two groups were exposed to NIR laser for 5 min. Results showed effective photothermal conversion ability in vivo. At 4 weeks post‐operation, rats looked normal and there was no localized swelling in the mandible. Micro‐CT 3D reconstruction images showed osseointegration in each group at 4 and 8 weeks (Figure 11D,E).

### 4.2 Surface Modification

For practical applications, hydrogels are often required to simultaneously possess many properties such as good biocompatibility, biodegradability, proper hydrophilic, good mechanical properties, and special functional groups which is achieved by designing multifunctional composites containing multiple natural polymers or synthetic polymers.^[^ ^15b^ ^]^

MXenes are 2D nanomaterials with unique properties – high conductivity and low toxicity, ideal for BTE applications. ‘M’ refers to the transition metal atom, ‘X’ to carbon/nitrogen, and ‘ene’ comes from ‘graphene’. They are interesting material for tissue engineering due to their unique properties.^[^ ^255^ ^]^ For example, for the practical applications of Xene‐based hydrogels such as those containing graphene, which is highly hydrophobic and is not evenly dispersed in an aqueous solution and hydrogels, a uniform distribution of graphene hydrogels is achieved by anchoring the surface with polar functional groups, including hydroxyl and carboxyl groups, through chemical modification by oxidation or reduction. However, both reduced graphene (rGO) and GO exhibit lower conductivity than original graphene since the covalent functionalization is known to potentially damage the original lattice altering its properties including the electric conductivity. Additionally, other classes of Xenes such as carbides, nitrides, or carbonitrides of transition metals are also capable of functioning as cross‐linking points in 3D platforms. With the advances in polymer chemistry, Xenes’ surface can be modified with the selective functional groups with target complementary moieties thus achieving augmented interactions in hydrogel precursors, and then preparing nanocomposite hydrogels via interdependent assembly. Meanwhile, such Xene‐hydrogel interactions possess high flexibility and adaptability to inscribe nanocomposites in the final hybrid structure.^[^ ^255^ ^]^ Therefore, any modification to the parent hydrogel structure, including the addition of 2D materials enhances the properties of the hydrogel, improving its efficiency. For example, Xu et al.^[^ ^256^ ^]^ designed a polydopamine‐modified black phosphorous (BP@PDA) nanosheet that incorporated gelatin methacryloyl (GelMA) hydrogels which significantly enhanced the electrical conductivity of the hydrogels while improving the cell migration of mesenchymal stem cells (MSCs) within the 3D scaffolds. The fabricated BP@PDA‐incorporated GelMA scaffold thus provided new insight into designing integrated biodegradable conductive BP nanomaterials within a biocompatible hydrogel for broad applications in tissue engineering of electroactive tissues, such as neural, cardiac, and skeletal muscle tissues. Conductive hybrid hydrogels are further discussed in detail in Section 4.1.

2D materials, such as MoS_2_ and WS_2_, have a wide range of biomedical applications, but their low processability and non‐specific interactions at bio‐interfaces pose a challenge. Researchers have studied the specific interactions between MoS_2_ and gelatin matrix hydrogels and showed that functionalization significantly improved their properties. Similarly, surface modification of WS_2_ nanosheets in combination with PVA provided a potential solution in processing self‐healable and mechanically strong hydrogels. These results suggest that functionalized TMDCs have great potential as highly‐processable biomaterials.^[^ ^257^ ^]^

### 4.3 Drug Delivery

Drug‐loaded bone scaffolds provide an attractive solution for bone tissue engineering as they combine the two fundamental aspects of therapy and regeneration that counter a significant challenge.^[^ ^258^ ^]^ While the scaffold plays the role of the structural physical support to attain bone repair and remodeling, drug‐loaded bone scaffolds act as local drug delivery systems that are capable of specifically targeting the bone tissue allowing additional treatment of the injured area and enhanced healing process.^[^ ^258^, ^259^ ^]^

Osteosarcoma is a common fatal tumor of the bone with the most common sites around the knee and proximal humerus. The peak incidence is reported in adolescents and adults greater than 60 years of age.^[^ ^260^ ^]^ The current form of osteosarcoma treatment involves the intravenous administration of Doxorubicin hydrochloride (DOXO); however, systematic delivery is known to be related to the onset of DOXO‐induced cardiomyopathy.^[^ ^261^ ^]^ The nanocomposite hydrogels incorporated with BP have been investigated for dual drug delivery and stimulating bone regeneration functions. In this regard, Qing et al.^[^ ^12b^ ^]^ designed an injectable multifunctional BP nanosheet (BPN) and doxorubicin (DOX) encapsulated chitosan‐based hydrogel (BP/DOX/CS) for synergistic photothermal chemotherapy of tumors and promoting osteogenesis. The fabricated BP/DOX/CS hydrogel displayed excellent photothermal effects under NIR irradiation due to the presence of BPN, good drug loading capacity, and continuous release of DOX from the gel matrix. The hydrogel also promotes the osteogenic differentiation of MC3T3‐E1 cells by releasing phosphate. Furthermore, in vivo results show that the fabricated composite hydrogel has good biocompatibility and can be injected at the tumor site to eliminate systemic toxicity. Similarly, Gan et al.^[^ ^262^ ^]^ developed a GelMA hydrogel incorporating BPN and deferoxamine (DFO) to overcome the high risk of bone delay union, where BPN controllably releases phosphorus ions^[^ ^263^ ^]^ and DFO can promote angiogenesis as an iron chelator. The resulting hydrogel scaffold showed superior degradation, good biocompatibility, and a sustained release of phosphorus ions which allows for improved osteogenesis at the bone defect site constantly. Similarly, a profile of the initial burst release of ferric ions in the first 24 h is observed with a constant slow release after 48 h. Furthermore, the in vivo results suggested that hydrogel could significantly improve osteogenesis and neovascularization in the bone site of Sprague Dawley (SD) rats with acute femoral artery occlusion. In a study conducted by Hua et al.^[^ ^5d^ ^]^ the results showed that MSC‐derived exosomes loaded with BP hydrogels promote bone regeneration in vivo by releasing exosomes and water molecules through the hydrogel's reversible cascade reaction. This approach provides a new treatment option for drug delivery and controlled release, with potential for bone tissue repair.

Researchers also provided proof‐of‐principle evidence for the application of drug‐loaded Ge‐based nanosheets (Ge NSs) as an intelligent tumor surgical adjuvant therapy. This surgical adjuvant therapeutic strategy is based on the fact that the exfoliated Ge NSs could be developed as drug‐delivery platforms to enable high loading capacity of chemotherapy drug (DOX), multi‐responsive (pH‐ and NIR‐sensitive) drug‐release behavior, NIR‐triggered deep tumor penetration, effective antitumor efficacy, high biocompatibility, and excellent theragnostic properties (multimodal‐imaging‐guided treatment). When combined with hydrogel composed of agarose and chitosan, the developed drug‐loaded Ge@hydrogel could be coated on the postoperative wound surface after tumor removal. Both hydrophilic drugs and hydrophobic drugs such as DOX and β‐elemene (loaded into polymeric nanoparticles) could be incorporated into this intelligent system.^[^ ^264^ ^]^

Shan et al.^[^ ^265^ ^]^ also developed injectable and thermos‐reversible BPNSs/docetaxel (DTX) ‐M‐hydrogel co‐encapsulating DTX micelles and BPNSs for synergistic chemotherapy and photodynamic therapy (PDT) to achieve sustained and light‐selective cancer damage with lower systemic toxicity. BPNSs/DTX‐M‐hydrogel could be intratumorally injected into the tumor as a sustained and localized depot which could sustainably release anticancer drug‐in vitro and in vivo. BPNSs exhibited superior PDT performance under 660 nm laser irradiation. Furthermore, with outstanding biodegradation and biocompatibility, BPNSs/DTX‐M‐hydrogel could attract more interest compared with other previously reported PDT agents. Moreover, the results of antitumor efficacy in vivo showed that the group using BPNSs/DTX‐M‐hydrogel with 660 nm laser irradiation exhibited powerful tumor inhibition among all treatment groups. While increasing anticancer efficacy, synergistic therapy could also reduce the drug dosage, thereby reducing side effects.

In recent years, researchers have focused on developing a more effective approach to drug delivery. One promising method is photothermal therapy (PTT), which involves using light to generate heat and trigger the release of drugs from a smart delivery system. By combining these two technologies, the hope is to create a synergistic effect that enhances drug delivery and improves treatment outcomes. Numerous studies have been conducted to explore the potential of this approach and develop innovative solutions that can be used in a clinical setting. PTT needs hyperthermia up to 50°C for tumor cell death. Too much heat can hurt adjacent tissues while insufficient heating causes tumor recurrence. Therefore, sensitization of tumor cells to PTT is crucial. Stress granules (SGs) help regulate cell viability under stress, but their role in PTT is unknown.^[^ ^215a^ ^]^ Fan et al.^[^ ^215a^ ^]^ found that PTT induces SGs through the eukaryotic initiation factor 2α‐dependent pathway, which causes resistance to BP nanosheets. A BP hydrogel delivers the SG inhibitor (Emetine) to modulate SG formation in tumors. NIR‐light irradiation enables PTT of the tumor, and light‐controlled release of Emetine inhibits PTT‐induced SG formation, sensitizing the tumor to PTT and improving tumor inhibition. In another study, He et al.^[^ ^215e^ ^]^ developed a smart delivery system for bufalin, a drug effective in treating tumors but with harmful effects on the heart. They used a BP hybrid polypeptide hydrogel that highly loads bufalin and achieved NIR‐controlled drug release with a synergistic photothermal‐chemo therapeutic effect. When combined with the photothermal‐chemo therapeutic effect, the system could efficiently eliminate tumors in vivo with good biosafety and biocompatibility. This work provides a new hydrogel platform for controlling bufalin release, promoting the practical application of antitumor therapy. Synergistic therapy using 2D materials incorporated hydrogels are explained in the Section 5.4.

### 4.4 Mechanical Reinforcement

With excellent potential for various biomedical applications, biopolymer‐based hydrogels suffer from low mechanical properties, uncontrolled degradation, and insufficient osteogenic activity. Therefore, their tendency to break often limits their application. However, the integration of secondary components like 2D nanomaterials in the hydrogel network expands the range of achievable mechanical properties while increasing the retention rate of local nanoparticles as well. Therefore, combining hydrogels with nanoparticles has shown promising solutions to expand their range of applications while showing a better capacity as bioactive reservoirs and providing controlled release rates of drugs.^[^ ^266^ ^]^ For example, the hydrogel used for bone repair requires strong mechanical properties matching the bone (Young modulus ≈30–60 kPa)^[^ ^252^ ^]^ while the hydrogel used for skin repair has weak mechanical properties matching the skin (Young modulus ≈5–20 kPa).^[^ ^267^ ^]^

Black phosphorus is an inorganic material that shows promise for use in bone repair, despite being prone to degradation in aqueous media. When black phosphorus nanosheets (BPNs) degrade, they transform into nontoxic phosphate, which is beneficial because phosphorus is a key component of bone and comprises ≈1% of the human body's weight. BP‐based hydrogels have impressive mechanical strength and intrinsic bioactivity. When placed in bone defect areas, BPNs attract bone‐forming cells to the site and capture signaling molecules that these cells release, triggering bone formation. The release of PO_4_^3−^, which is a representative anionic ligand for calcium ions extracted from physiological environments, is significantly affected by the oxidation process of BP nanosheets. Additionally, this process plays a crucial role in facilitating the creation of new calcium phosphate nanoparticles.^[^ ^225^ ^]^ During bone remodeling, proper calcium and phosphorus metabolism is necessary, along with osteoblasts and osteoclasts, to encourage bone mineralization.^[^ ^274^ ^]^ BP's degradation products are benign phosphates that play a role in encouraging calcium deposition in bone tissues, ultimately facilitating bone restoration. The nanocomposite hydrogel boasts a compressive modulus that is more than double that of the pure hydrogel group, as well as the ability to foster increased surface mineralization, which can aid in the formation of new bone.^[^ ^275^ ^]^ The team of Huang and coworkers^[^ ^275^ ^]^ created a composite hydrogel system specifically designed for bone tissue engineering. This innovative system utilizes BP nanosheets, GelMA, and cationic arginine‐based unsaturated polyesteramide. The inclusion of BPNs within the hydrogel enhances its mechanical strength while also promoting the release of phosphorus ions during degradation, which is essential for bone defect repair. Testing has revealed that the compressive modulus of the composite hydrogel is 3–4 times higher than that of the pure hydrogel. Both in vitro and in vivo experiments demonstrate that the composite hydrogel generates more mineralization than the pure hydrogel, resulting in a more effective final bone repair.

**Figure 12:**
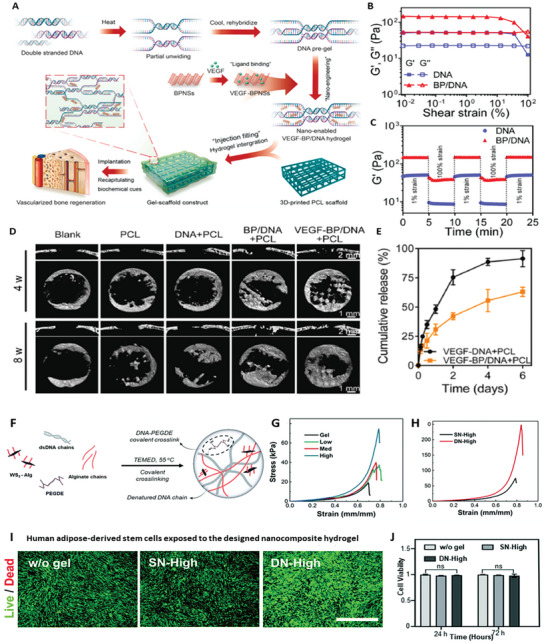
A) Schematic illustration of the concept of integrating 3D‐printed PCL scaffold and BPNSs‐enabled DNA hydrogel loaded with VEGF for vascularized bone regeneration. B) Strain sweep measurement of hydrogels: The change of the storage and loss moduli indicated the linear viscoelastic region at 0.01%–10%. C) Mechanical recovery of hydrogels as determined by the change of storage modulus under alternating high (100%) and low strain (1%). D) Micro‐CT images of cranial defects treated with constructs, including cross sections and longitudinal sections. E) The release profiles of VEGF from the gel‐scaffold constructs. Reproduced with permission.^[^ ^222^ ^]^ Copyright 2022, Elsevier. F) Schematic depicting the method for developing single network DNA‐WS_2_ hydrogels. Here, double‐stranded DNA (dsDNA) chains were covalently cross‐linked with PEGDE to form the desired nanoparticle‐impregnated material. G) Stress versus strain plots of the DNA‐WS_2_ hydrogels containing different concentrations (“Low”, “Medium”, and “High”) of WS_2_. H) Stress versus strain plots of single and double network hydrogels containing the highest concentration of WS_2_. I) Fluorescence micrographs depicting the biocompatibility of the designed nanocomposite hydrogels. Human adipose‐derived stem cells were exposed to the designed hydrogels for 72 h, after which the fluorescence images were collected (scale bar = 1000 µm) J) MTS assay of human adipose stem cells after 24 and 72 h of contact with the respective hydrogels. Reproduced with permission.^[^ ^215d^ ^]^ Copyright 2022, Royal Society of Chemistry.

It is widely recognized that bone remodeling and regeneration rely on proper vascularization to maintain metabolic balance.^[^ ^276^ ^]^ In mature bone, the vascular system provides the necessary signaling factors and nutrients to act as a communication bridge between bone and adjacent tissues.^[^ ^277^ ^]^ The high surface area of BPNSs and noncovalent interactions with bioactive molecules, such as electrostatic complexation, hydrophobicity, and *π*–*π* stacking, probably ensure high loading efficiency and sustained release profile of bioactive molecules, such as vascular endothelial growth factor (VEGF).^[^ ^278^ ^]^ As mentioned previously, one approach to improve the mechanical strength of hydrogels is to improve the degree of cross‐linking.^[^ ^268^ ^]^ For example, Huang et al.^[^ ^267^ ^]^ developed a composite polymer hydrogel with GelMA and hyaluronic acid grafting dopamine. The obtained hydrogel showed better mechanical properties with the increased cross‐linking degree of the GelMA. More recently, Chen et al.^[^ ^222^ ^]^ engineered a dynamic DNA hydrogel with vascular endothelial growth factor (VEGF) decorated BP nanosheets and integrated with a 3D‐printed polycaprolactone (PCL) scaffold to develop bioactive gel‐scaffold construct for a dual regulatory effect on angiogenesis and osteogenesis for cranial bone regeneration (**Figure** **12**A–E). Incorporating 2D BP nanosheets (BPNSs) into DNA dynamic hydrogel led to the mechanical reinforcement of the reversible network where the BPNSs significantly increased the storage modulus of the DNA hydrogel. The physical interactions between BPNSs and the DNA backbone regulate the mechanical stability of the hydrogel by forming additional cross‐linking points. A sustained release of VEGF was observed due to the non‐covalent interactions between VEGF and BPNSs.

In a recent study by Wang et al.^[^ ^281^ ^]^ a combination of methacrylate gelatin, polysaccharides (alginate and chitosan), inorganic BP and calcium phosphate (CaP) nanocrystals, were used to synthesize hydrogels. The elastic modulus of DN hydrogels was significantly enhanced through covalent cross‐linking bonds between the double networks. Among covalently cross‐linked DN hydrogels, PAM/AlgMA and PAM/ChiMA hydrogels demonstrated superior mechanical strength. Increasing the proportion of acrylamide monomer resulted in higher toughness and elastic modulus for dual cross‐linked DN hybrid gels. The study concluded that the simultaneous formation of covalent and noncovalent interactions between the natural and synthetic polymeric networks is a more effective and widely applicable approach to strengthen the mechanical properties of the DN gels.

In another work, Paul et al.^[^ ^215d^ ^]^ utilized DNA as a natural biopolymer and 2D nanosheets of WS_2_ to fabricate a mechanically tough nanocomposite hydrogel (Figure 12F–H). In the presence of alginate‐exfoliated WS_2_ nanosheets, the single network hydrogels were formed via covalent cross‐linking of DNA chains. The DNA strands were joined via chemical cross‐linking using a polyethylene glycol dioxide (PEGDE) bi‐functional cross‐linker with epoxide end groups. The alginate chains in hydrogel formulation were thereafter ionically cross‐linked with Ca^2+^ ions to form DN polymeric hydrogels. The mechanical and structural properties were tested with oscillatory shear rheology and uniaxial compression testing which elucidated the advantageous effects of nanosheets and the formation of a DN. Furthermore, the biocompatibility with hASCs was confirmed with in vitro cytotoxicity assays (Figure 12I,J). Therefore, DNA‐based hydrogels can be effectively used as a scaffold for various biomedical applications, including the delivery of drugs such as proteins and growth factors.

Furthermore, in one of the previously mentioned studies by Kamali et al.^[^ ^257b^ ^]^ the WS_2_ exfoliated nanosheets modified using organic thiol groups, impart hydrogen bonding sites on the inert nanosheets by surface functionalization. This modification contributes to the PVA hydrogel matrix's intrinsic self‐healing and mechanical properties. The PVA hydrogels prepared using pristine WS_2_ nanosheets show a much higher tensile strength of 636.3 kPa, due to the reinforcement of polymeric chains by WS_2_ nanosheets in comparison to parent PVA hydrogels which possess a tensile strength of 261.4 kPa. Furthermore, the addition of thiol‐modified WS_2_ to the hydrogel matrix significantly increases Young's modulus of the hydrogel, making it much more resistant to deformation than pure PVA hydrogel. Therefore, these results open an avenue toward developing self‐healing nanocomposites where chemically inert nanoparticles participate in the healing network rather than just mechanically reinforcing the matrix by slender adhesion.

Under varying conditions, producing a hydrogel possessing both robust mechanical strength and self‐healing capabilities through conventional techniques can be challenging. The former characteristic arising from robust and covalent bonds within the gel network while the latter is a product of dynamic and non‐covalent interactions.^[^ ^282^ ^]^ In order to achieve good mechanical properties, strong interactions between hydrogel moieties are necessary, but their mobility is also required to facilitate the reoccurrence of interactions around cracks and repair damages.^[^ ^283^ ^]^ A system that utilizes multivalent noncovalent interactions could incorporate these contrasting characteristics.^[^ ^284^ ^]^ Because the sum of net interactions is strong enough to hold hydrogel components but it is reversible to recover the damages.^[^ ^285^ ^]^ Gelatin's weak mechanical properties and inability to recover defects affect its efficiency in tissue engineering, making it less useful.^[^ ^286^ ^]^ However, the combination of gelatin and MoS_2_ sheets results in hybrid hydrogels that possess useful mechanical and biological properties such as self‐healing and molecular recognition features. A study by Zebardasti et al.^[^ ^248a^ ^]^ analyzed the mechanical properties of neat gelatin and MoS_2_gel hydrogels by analyzing the compressive properties. The compressive strength of MoS_2_ gel containing 1% CDMoS_2_ was found to be three times higher than that of neat gelatin. This was due to the multivalent host‐guest interactions between CDMoS_2_ and adamantly segments embedded in gelatin. Li et al.^[^ ^288^ ^]^ found that adding rGO to GM/acryloyl‐β‐cyclodextrin (Ac‐CD) hydrogels improved their mechanical strength. The hydrogels were able to withstand deformation without significant damage. The GM/Ac‐CD/rGO_0.6_ hydrogel maintained its structural stability under repeated external mechanical forces. The higher content of the rGO nanocomposite increased the cross‐linking density of GM/Ac‐CD/rGO hydrogels, resulting in improved toughness.

Therefore, incorporating 2D nanomaterials like BPNs, MoS_2_, and rGO into hydrogels presents a significant advancement in bone tissue engineering, enhancing mechanical properties and promoting calcium deposition for bone restoration. Supported by experimental data, these enhancements offer promising avenues for mimicking natural bone tissue integrity and bearing capacity, paving the way for improved bone repair and regeneration strategies.

### 4.5 Electrical and Photothermal Stimulation

In the realm of scaffold‐mediated bone tissue engineering, photo‐triggered and electrically conductive hydrogels provide promising functionalities as they can be triggered by light or electrical field at a specific wavelength and voltage providing the final hydrogels with specific photonic and electrical properties.^[^ ^269^ ^]^ Additionally, since the structural anisotropy of 2D materials such as BP results in their unique properties including electrical conductivity, optical, thermoelectric, and topological features, and unusual mechanical behavior, their incorporation into hydrogels to form nanocomposite hydrogels also opens up the possibility of inducing electrical or photothermal simulation through hydrogel scaffolds to enhance bone regeneration.^[^ ^270^ ^]^ Due to this, BP and other 2D nanomaterials have gained huge attention for their biomedical applications such as PTT, PDT, light triggered drug delivery, and bioimaging.

**Figure 13:**
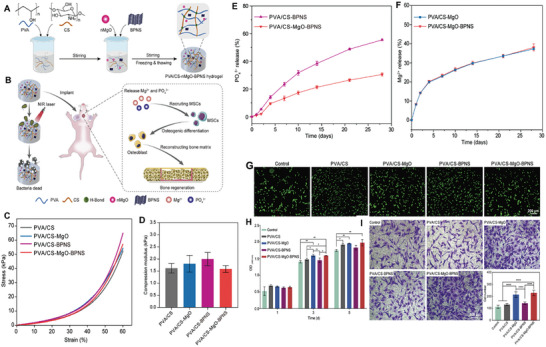
A) Schematic diagram of the preparation of PVA/CS‐MgO‐BPNS hydrogel. B) PVA/CS‐MgOBPNS hydrogel exhibited a superior antibacterial effect under NIR irradiation and potentiated bone regeneration in situ by recruiting MSCs and promoting bone matrix reconstruction. C) Stress‐strain curves D) and compression modulus of different groups. E) Cumulative release of Mg^2+^ from the PVA/CS‐MgO and PVA/CS‐MgO‐BPNS hydrogels. F) Cumulative release of PO_4_^3−^ from the PVA/CS‐BPNS and PVA/CSMgO‐BPNS hydrogels. G) Viability of MSCs cultured with hydrogels as determined by Live/Dead staining. H) Cell proliferation was evaluated with the CCK‐8 assay. I) MSC migration was evaluated by the trans‐well migration assay after 12 h of culture; the number of migrated cells was determined by averaging the counts from five random fields per well. Reproduced with permission.^[^ ^271^ ^]^ Copyright 2022, Elsevier Ltd. All rights reserved.

The photothermally active BP can be exploited for its versatile utility in combination with a hybrid hydrogel system. Tan et al.^[^ ^244^ ^]^ fabricated a composite remotely‐activable ECM‐mimetic chitosan/collagen‐based hydrogel integrated with mesenchymal stem cell (MSC)‐membrane‐coated BP. The results revealed that the ECM‐mimetic BP‐incorporated hydrogel could enhance osteoblast migration/differentiation and stimulate the biomineralization process under remote NIR activation to promote bone healing, which offers new opportunities for repairing cranial defects in the clinics. Similarly, Qin et al.^[^ ^271^ ^]^ designed a multifunctional hydrogel for synergistic photothermal and antibacterial bone regeneration by adding magnesium oxide nanoparticles and BPNSs into PVA/chitosan hydrogel (**Figure** **13**A–I). The prepared PVA/CS‐MgO‐BPNS hydrogel could kill more than 99.9% of *Staphylococcus aureus* (S. *aureus*) and *Escherichia coli* (E. *Coli*) under the synergistic effect of NIR irradiation and CS intrinsic antibacterial properties. Additionally, the released Mg^+^ ions stimulate the migration of MSCs to hydrogels and synergize with released phosphate to promote osteogenic differentiation while also promoting the calcium phosphate particle formation therefore improving the biomineralization ability. Thus, the BP nanomaterials are often doped into 3D hydrogel scaffolds for bone regeneration in which they could function in multiple ways for bone growth and therapy.^[^ ^272^ ^]^

Pan et al.^[^ ^226^ ^]^ combined BP nanosheets into a platelet‐rich plasma (PRP) chitosan thermal responsive hydrogel for the treatment of arthritis and bone defects caused by rheumatoid arthritis (RA). The local heat generated by BP under NIR irradiation along with the generated reactive oxygen species (ROS) are transferred to the diseased joint to remove the proliferative synovial tissue. This was accompanied by precisely controlling the release of BP degradation products to provide sufficient raw materials for osteogenesis and promoting the repair of bone defects caused by RA.

Kamali et al.^[^ ^257b^ ^]^ fabricated PVA nanocomposite hydrogels by physical cross‐linking methods and investigated the samples of hydrogels with only PVA, pristine WS_2_ nanosheets (PVAW), Dithiothreitol (DTT)‐modified WS_2_ (PVAFW), and hydrogels with DTT‐modified WS_2_ nanosheets for their intrinsic self‐healing properties. Healing efficiency of 98.2% and 93% were achieved using microwave and NIR laser irradiation respectively, along with a shorter required time due to the excellent photothermal conversion and microwave absorption properties of WS_2_ nanosheets. The H‐bonding is facilitated with microwave and NIR irradiation due to the thermally induced motion of polymeric chains resulting in chain slippage, cleavage, and reformation at the rupture surface. Additionally, the issues arising from improper alignment of polymeric chains become less significant because new bonds are formed on the newly created interface by the large number of chain arrangements induced by the thermal motions.

Conductive hydrogels not only can replicate the ECM of natural bone tissue but also can effectively imitate and expand the effects of endogenous electric fields, hence constructively repairing the damaged bone tissue. Therefore, since electrical stimulation (ES) can remarkably aid bone healing, a conductive scaffold that can deliver ES locally at the affected site is desirable for bone defect healing.^[^ ^273^ ^]^ The conductive properties of conventional hydrogels are not particularly high and are often increased by the addition of 2D materials. It has been observed that in the GelMA hydrogels, the electrical impedance values dramatically decreased to 12 kΩ after BP loading, while that of GelMA hydrogel was 46 kΩ.^[^ ^256^ ^]^ Liu et al.^[^ ^225^ ^]^ fabricated BP and carbon‐nanotube (CNT) co‐loaded hydrogels for bone injury repairment, and the synergy of these materials witnessed better bone tissue engineering effects due to the increased electrical conductivity of the carbon tube under electrical stimulation. In another example, Jing et al.^[^ ^274^ ^]^ proposed a photosensitive conductive hydrogel incorporating magnesium‐modified black phosphorous into GelMA that can induce skeletal‐associated neural network reconstruction and bone regeneration with high antibacterial activity for the treatment of infected bone defects. The conductive nanosheets and bioactive ions released from BP@Mg synergistically improve the migration and secretion of Schwann cells, promoting neurite outgrowth while facilitating innerved bone regeneration as well.^[^ ^256^ ^]^Therefore, the development of multifunctional scaffolds has become a promising strategy to effectively promote bone defect repair.

**Figure 14:**
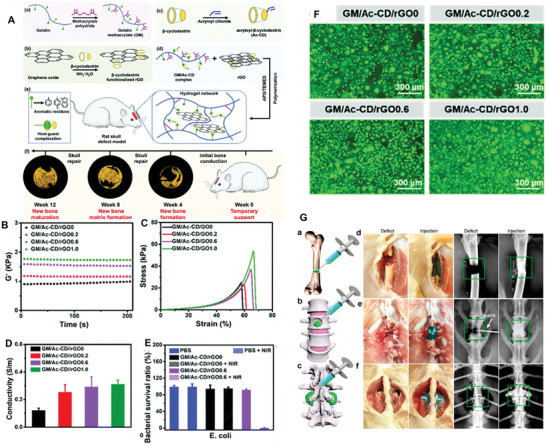
A) Scheme of the fabrication of GM/Ac‐CD/rGO hydrogels. The synthesis of GM (a), rGO (b), and Ac‐CD (c); (d) Schematic diagram of the GM/Ac‐CD/rGO hydrogel network constructed by double‐bond radical polymerization and host–guest complexation; (e) Application of the hydrogel patch as an implant in initial bone conduction; (f) Skull bone repair in a rat skull defect model. B) The *G*′ of GM/Ac‐CD/rGO hydrogels. C) The compression and stress‐strain of GM/Ac‐CD/rGO at strains of 20%. D) Conductivity of GM/Ac‐CD/rGO hydrogels. E) Photothermal‐induced antibacterial properties of GM/Ac‐CD/rGO hydrogels against *E. coli. F)* MC3T3‐E1 cell growth on GM/Ac‐CD/rGO hydrogels after co‐incubated for 7 days. Reproduced with permission.^[^ ^275^ ^]^ Copyright, Royal Society of Chemistry. G) In vivo bone filling ability of the in situ‐gelling gel developed by Liu et al.^[^ ^225^ ^]^: diagrams, photographs, and X‐ray images of bone defects and gel injection for rabbit (a,d) femur defect, (b,e) vertebral body defect and (c,f) posterolateral spinal fusion. Reproduced with permission.^[^ ^225^ ^]^ Copyright 2020, American Chemical Society.

In another example, Li et al.^[^ ^275^ ^]^ fabricated a series of multifunctional hydrogels based on gelatin methacrylate (GM), acryloyl‐β‐cyclodextrin (Ac‐CD), and β‐cyclodextrin (β‐CD)‐functionalized rGO for skull defect regeneration having stable mechanical properties, non‐swelling property, conductivity, and photothermal antibacterial properties (**Figure** **14**A–G). The Ac‐CD was added as a host macromolecule to improve the toughness of the hydrogels with rGO as the conductive element to possess the hydrogel conductive properties. The in vitro/in vivo results confirmed the biocompatibility of the GM/Ac‐CD/rGO hydrogel with a simultaneously promoting effect for the proliferation and osteogenic differentiation of MC3T3‐E1 cells after 7 and 14 days of incubation, and further accelerated in vivo bone defect repair in a rat skull defect model. The results exhibited that these multifunctional hydrogels have shown promising applications in bone tissue formation and accelerated bone defect repair, indicating their great potential for clinical application.

**Table** **3** summarizes the studies on 2D materials incorporated composite hydrogels for bone regeneration.

**Table 3:** Summary of the properties and biological performances of the nanocomposite hydrogels.

Hydrogel	2D material	Properties	Biological performance	References
DNA bioactive gel scaffold	BP nanosheets	In the strain range of 0.01%–10%, both storage modulus G′ and loss modulus G″ of DNA and BP/DNA hydrogels were constant with the values of G′ higher than G’’ with BPNSs increasing the storage modulus of DNA hydrogel.	The VEGF‐BP/DNA + PCL construct demonstrated a superior capacity for cranial defect regeneration. The highest bone formation percentage and density of new bone were observed in the VEGF‐BP/DNA + PCL construct.	[222]
GelMA‐BP@PDA scaffold	BP	Zeta potential BP@PDA nanosheets: – 61.85 mV The storage modulus (*G*′) and loss modulus (*G*″) correspondingly increased with the increased amount of BP@PDA. Impedance value: GelMA–BP@PDA‐0.3 (≈12 kΩ) << GelMA hydrogel (≈46 kΩ). Highest conductivity: GelMA–BP@PDA‐0.3 (0.19 S m^−1^.)	–	[256]
MoS_2_/gelatin	β‐Cyclodextrin modified‐MoS_2_ (CDMoS_2_)	The compressive strength of 1% CDMoS_2_ gel is three times higher than that of the neat gelatin, due to the multivalent host‐guest interactions between CDMoS_2_ and adamantly segments embedded in gelatin.	–	[257a]
PVA hydrogel matrix	Exfoliated 2H‐WS_2_ (thiol functionalized) nanosheets modified with Dithiothreitol (DTT)	Tensile strength: DTT‐WS_2_/PVA hydrogel (748.5 kPa) > WS_2_‐PVA hydrogels (636.3 kPa) >> PVA hydrogels (261.4 kPa). Young's modulus: DTT‐WS_2_/PVA > PVA Self‐healing ability: 18.76% of the original tensile strength could be recovered for WS_2_‐PVA. It can be increased to 89.92% by soaking the fresh hydrogels in (NH_4_)_2_SO_4_. Healing efficiency of 98.2% and 93% using microwaves or NIR laser, respectively.	–	[257b]
Gelatin	Deferoxamine – BP nanosheets (BPN‐DFO)	–	Col‐I, BMP4, and RUNX2 were all up‐regulated in the BNP‐DFO hydrogel group after 7 and 14 days of incubation, indicating that hydrogel can promote the osteogenesis of hBMSC in vitro.	[262]
DNA hydrogel scaffold	Alginate exfoliated‐ WS_2_ nanosheets	‐ An increase in the ultimate stress with the addition of exfoliated WS_2_ to the DNA‐based system ‐ A significant increase from 3.62 to 8.13 kPa with an increase in WS_2_ concentration from 0 to 0.75%. ‐ The ionically cross‐linked alginate network enables the DN system to possess improved shear properties in comparison to the single network hydrogel.	MTS assays, performed after 24 and 72 h, displayed greater than 95% cell viability for both single and DN systems confirming the non‐toxic nature of WS_2_ as well as the formulated single and DN hydrogels	[215d]
(GelMA)/ hyaluronic acid graft dopamine (HA‐DA)	β‐cyclodextrin (βCD)‐functionalized GO	‐ The higher the GelMA content, the higher the *G*′ ‐ The 10% GelMA and 5% HA‐DA exhibited the best mechanical strength.	‐ The cycle heating test showed that GO‐βCD exhibited photothermal stability with the majority of bacteria killed when the temperature reaches 50 °C ‐ The concentration of Nitric oxide released from BNN6 under NIR irradiation was higher than that without NIR.	[267]
GelMA (GM), Acryloyl‐β‐cyclodextrin (Ac‐CD), and β‐cyclodextrin (β‐CD)‐functionalized hydrogels	r‐GO	The storage modulus G′ of GM/Ac‐CD/rGO hydrogel gradually increased from 0.99 to 1.16, 1.53, and 1.73 kPa as the content of the rGO in the hydrogel increased from 0 to 0.2, 0.6, and 1.0 mg mL^−1^ Maximum compression strain for GM/Ac‐CD/rGO0, GM/Ac‐CD/rGO0.2, GM/Ac‐CD/rGO0.6, and GM/Ac‐CD/rGO1.0 was ≈59.0, 60.2, 65.1, and 68.9%, respectively. The GM/Ac‐CD/rGO0.6 hydrogel can recover to its original state from a compressed state in a short time once the loading was released at strains of 20%, 40%, and 60% Conductivity for the hydrogel GM/Ac‐CD/rGO0.2, GM/Ac‐CD/rGO0.6, and GM/Ac‐CD/rGO1.0 increased from 0.25 to 0.29 and 0.31 S m^−1^, respectively, due to rGO providing a pathway for electron transfer.	NIR‐induced photothermal antibacterial property of the GM/Ac‐CD/rGO0.6 hydrogel group with a 100% killing ratio of both S. aureus and E. coli with 10 mins of exposure. rGO nanocomposite significantly increased protein adsorption up to three times the GM/Ac‐CD/rGO0.6 hydrogel exhibited denser in ALP staining than in other groups, higher level of ALP genes of the GM/Ac‐CD/rGO0.6 group. New bone coverage area in the GM/Ac‐CD/rGO0 group and GM/Ac‐CD/rGO0.6 group was significantly more compared with the control groups at 4, 8, and 12 weeks.	[276]
Platelet‐rich plasma (PRP)‐chitosan hydrogel	BP nanosheets	The temperature of BPNs/Chitosan/PRP thermoresponsive hydrogel increased by ≈25 °C under the uniform NIR irradiation	Hydrogel solution in a liquid state caused explosive release of MTX within 24 h, and then reached the plateau phase. In comparison, the gel solution after NIR irradiation caused the MTX to be slowly released.	[226]
Carbon nanotube ‐poly(ethylene glycol)‐acrylate (CNTpega)	BP	‐ The CNTpega‐gel displayed the highest strength ‐ The incorporation of BP nanosheets was found to slightly reduce the mechanical strength of the gels. ‐ Approximately 0.008 S m^−1^ was the highest observed at a CNTpega concentration of 16 mg mL^−1^ and a BP nanosheet concentration of 0.8 mg mL^−1^.	Cells cocultured with BP‐gel and BP‐CNTpega‐gel showed faster proliferation with a higher density of cells determined. ‐ Elevated proliferation under electrical stimulation. ‐ The BP‐CNTpega‐gel was able to fill the femur and vertebral defect, forming a stable gel that bridged both sides of the fracture location.	[225]
GM	Acryloyl‐β‐cyclodextrin (Ac‐CD), and β‐cyclodextrin (β‐CD)‐functionalized rGO	‐ The Storage modulus *G*′: Increased for GM/Ac‐CD/rGO from 0.99 to 1.16, 1.53, and 1.73 kPa with 0, 0.2, 0.6, and 1.0 mg mL^−1^ rGO content respectively ‐ The GM/Ac‐CD/rGO hydrogels with different rGO contents had no apparent damage and the network of the hydrogel under each formula still maintained integrity under the strain of 40%.‐The Δ*T*_s_ increased from 5.0 °C to 11.0 °C and 14.0 °C for GM/Ac‐CD/rGO0.2, GM/Ac‐CD/rGO0.6 and GM/Ac‐CD/rGO1.0, respectively,.	‐ No bacteria survived in the GM/Ac‐CD/rGO0.6 hydrogel group and the killing ratio of both *S. aureus* and *E. coli* reached 100% when the irradiation time was extended to 10 min. ‐ After culturing for 7 days, cell proliferation with the GM/Ac‐CD/rGO hydrogels was higher than TCP. ‐ The Col I protein levels of the GM/Ac‐CD/rGO0.6 group were greater than both GM/Ac‐CD/rGO0 and control group after culturing for 7 days, and the OCN protein levels in the GM/Ac‐CD/rGO0.6 group were higher than that in the other two groups at both day 7 and day 14 ‐ Bone volume/tissue volume (BV/TV) of the GM/Ac‐CD/rGO0.6 hydrogel group was higher than that in the GM/Ac‐CD/rGO0 and control group at any time point	[275]

## 5 Applications of 2D Materials‐Based Hydrogels for Bone Cancer Therapy

The diagnosis, treatment, and prognosis of bone cancers remain a formidable challenge in the clinical scenario. Some common and severely dangerous bone malignancies are osteosarcoma, chondrosarcoma, and Ewing's sarcoma. Limitations in existing conventional cancer therapies such as surgical resection, chemotherapy, and radiation therapy warrants newer modalities with multi‐functionalities.^[^ ^277^ ^]^ Trends in advanced strategies focus on targeted therapy, minimizing cytotoxic effect on healthy tissues, combinations that address immunotherapy, simultaneous diagnosis/prognosis, toward a holistic approach for bone cancer therapy. The innovative cancer therapeutics approaches harness the optical properties of 2D materials, their large specific surface area for photo‐responsive therapies mediated by near infrared (NIR) irradiation and serve as carriers for various drugs.^[^ ^277^, ^278^ ^]^ 2D materials including graphene, GO, black BP, MXenes, and TMDCs exhibit an array of characteristics ideal for bone cancer therapeutics through various mechanisms such as oxidative stress, apoptosis, and autophagy.^[^ ^277^, ^279^ ^]^ Interestingly, the potential for selective localized cytotoxic effects against cancer cells, with possibility of sparing the normal tissue from adverse effects, gains attention on 2D materials as emerging anti‐cancer modalities.^[^ ^277^, ^279^ ^]^ Various 2D materials‐based hydrogel strategies have been designed for bone cancer therapy by targeted drug delivery, photo‐therapy, combination therapy, theragnostic and theragenerative application. The progress in this direction promises multi‐functional modalities for potential clinical application against bone cancer.

### 5.1 PDT and PTT

**Figure 15:**
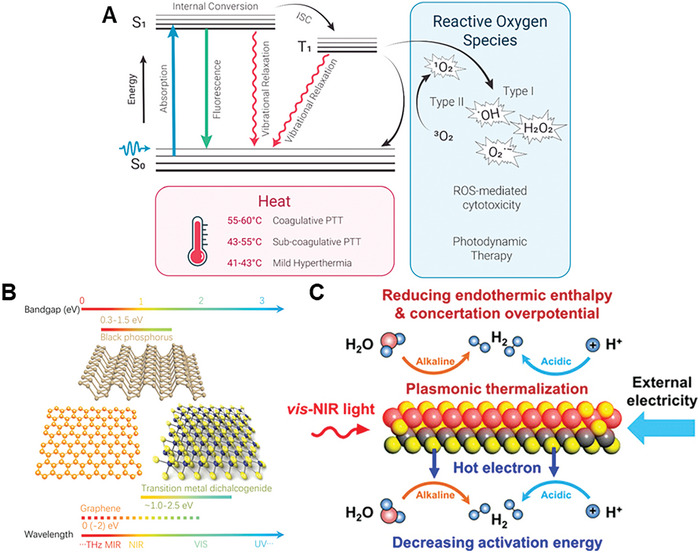
A) Simplified Jablonski diagram illustrating the mechanism of photosensitization processes in PDT and PTT. (S_0_ − ground state, S_1_ – excited singlet state, T_1_ − excited triplet state, ISC − intersystem crossing). Reproduced with permission.^[^ ^281^ ^]^ Copyright 2023, American Chemical Society. B) Electronic bandgaps of typical 2D materials. Intrinsic single‐layer graphene is a zero bandgap semi‐metallic material while its Fermi level can be tuned up to 1 eV under external electric field, covering the range from THz to visible wavelength. The TMDCsand BP have layer number‐dependent bandgap. Reproduced with permission.^[^ ^284^ ^]^ Copyright 2017, Wiley‐VCH. C) Schematic illustration of the localized surface plasmon resonances (LSPR)‐induced photothermal and hot electron effects of MXenes. Reproduced with permission.^[^ ^286^ ^]^ Copyright 2021, Wiley‐VCH GmbH.

The light responsiveness of 2D materials has been harnessed for PDT and PTT to combat cancers. These photo‐therapies are non‐invasive and patient‐specific, rendering localized treatment targeting cancer cells while sparing non‐malignant cells.^[^ ^280^ ^]^ In PDT, photosensitizers (PS) or photoactivated molecules excited by light triggers photochemical reactions that efficiently destructs cells/tissues, while PTT uses selective local heating.^[^ ^280^ ^]^ PDT is an emerging alternative oncologic intervention for tumor cell ablation and oxidation.^[^ ^278^ ^]^ In general, PS in PDT requires oxygen, which undergo photodynamic reaction and generate cytotoxic ROS. On light absorption, a PS molecule converts from the ground state to a short‐lived excited singlet state, which undergoes intersystem crossing to a relatively more stable excited triplet state (**Figure** **15**A). During the return of PS to ground state, type I or type II photodynamic reactions occur. In type I reaction, activated PS transfers electron to a substrate to generate various ROS (such as O_2_^•−^, HO^•^_2_), while in type II, direct transfer of energy to molecular oxygen creates highly reactive singlet oxygen (^1^O_2_). Interestingly, the generated ROS have extremely low diffusion distance within cells and tissues that enable spatiotemporal cytotoxicity on tumors, while very mildly affecting the surrounding cells.^[^ ^278^, ^281^ ^]^ Enhanced PDT has witnessed three generations of PSs for higher absorption wavelengths and improved targeting strategies.^[^ ^282^ ^]^ In this arena, 2D materials including GO, MXene, BP, and TMDCs have gained attention for improved and enhanced PDT.^[^ ^282^ ^]^ The electronic energy band structure of 2D materials determine the photoexcitation process.^[^ ^283^ ^]^ Graphene is a semimetal with valence state band (VB) and conduction band (CB) in contact and hence zero overlap.^[^ ^283^ ^]^ However, GO has π‐conjugated sp^2^ domains that create larger bandgap by confining electrons and holes in smaller space, which is surrounded by insulating matrix of sp^3^ domains.^[^ ^283^ ^]^ BP is a metal‐free semiconductor with a direct bandgap associated with the layer. The bandgap sensitively dependent on the number of BP layers, varies from 0.3 to 2 eV.^[^ ^283^, ^284^ ^]^ Similarly, in MoS_2_ (a TMDC) bandgap varies with its thickness, from indirect bandgap in bulk to direct bandgap in monolayer. As the number of MoS_2_ layers increases, the corresponding bandgap energy will decrease from 1.9 to 1.29 eV. Thus, GO, BP and TMDC exhibit direct and indirect band semiconductor properties (Figure 15B). They exhibit similar ROS generation mechanism for on light excitation.^[^ ^283^ ^]^ For instance, above bandgap light excitation of TMDC results in photogenerated electron‐hole pairs in the CB and VB. The photoexcited electrons in the CB with singlet character can relax to a band with triplet character, which then transfers energy to triplet oxygen (^3^O_2_) and forms ^1^O_2_, in O_2_ presence. Meanwhile, in the presence of water, the photogenerated electron‐hole pairs react with H_2_O and dissolved O_2_ to form ^•^OH and O_2_^•−^. The ROS production process involves both electron transfer and energy transfer between TMDC and H_2_O and O_2_.^[^ ^283^ ^]^ In MXenes, the mechanism varies, as they are metalloids and have a high free electron density. The ^1^O_2_ generation mechanism of MXenes is attributable to localized surface plasmonic resonance (LSPR) effect.^[^ ^285^ ^]^ The generation of ^1^O_2_ occurs by transfer of photo‐excited electrons from Ti_3_C_2_ to nearby oxygen molecules.^[^ ^283^ ^]^

Similar to PDT, PTT is considered an alternative oncologic medicine for temperature‐mediated ablation of tumor cells.^[^ ^278^ ^]^ In PTT, the temperature raises and causes local photocoagulation on light (NIR region) irradiation.^[^ ^281^ ^]^ The intrinsically high NIR absorbance characteristic property of 2D materials is exploited for PTT.^[^ ^281^, ^287^ ^]^ The layers of 2D materials that determines the unique tunable bandgap (0.3–2.0 eV for BP), the thickness‐dependent, high charge mobility regions for NIR light absorption determines the photothermal conversion efficiency (PCE).^[^ ^287^ ^]^ Ultrathin structures of 2D materials show rapid photo‐response and provide excellent in‐plane electron mobilities to achieve high PCE.^[^ ^288^ ^]^ Interestingly, MXenes exhibit maximum light‐to‐heat conversion efficiency (100%) with promising photothermal application. MXenes have free charge carrier densities due to the presence of transition metals and exhibit a strong electromagnetic interference (EMI) shielding effect attributed to its metallic conductivity. The electromagnetic waves undergo multiple internal reflections within layered structures of MXene that leads to more absorption. In addition to the EMI shielding effect, the abundant free charge carriers of MXenes also bring about LSPRs (Figure 15C).^[^ ^288^ ^]^ Based on the power of irradiation and PCE, PTT achieves sub‐coagulative (43–55 °C) or coagulative (55–100 °C) temperatures to induce rapid cell death via protein denaturation and cell membrane damage.^[^ ^281^ ^]^ Localized hyperthermia (41−43 °C) with milder heating increase selectivity and susceptibility of tumor cells to chemotherapy or radiation therapy, by inducing heat shock proteins and altering tumor perfusion and metabolic state.^[^ ^278^, ^281^ ^]^

The intrinsic demerits of 2D materials for PTT such as short‐term therapeutic and inhomogeneous photothermal effect and easy oxidation (or natural degradation) can be potentially overcome by designing nanocomposites.^[^ ^289^ ^]^ Xing et al.,^[^ ^289^ ^]^ developed smart cellulose/BP nanosheets (BPNS) nanocomposite hydrogels via a facile, green chemical cross‐linking in alkaline condition. The synthesis of BPNS‐integrated cellulose (cellulose/BPNSs) hydrogels was prepared by gelation of cellulose chains with the aid of epichlorohydrin (ECH) as a cross‐linker in the presence of BPNSs.^[^ ^289^ ^]^ BPNS‐integrated cellulose hydrogels exhibited multi‐pores with good mechanical properties and NIR‐induced photothermal potential attributed to BP. These composite hydrogels have demonstrated biosafety and biocompatibility for both in vitro and in vivo conditions. Further, on intratumoral administration, the composite hydrogels showed effective PTT against cancer in mice.^[^ ^289^ ^]^ Shao et al.,^[^ ^290^ ^]^ designed a sprayable PTT system by incorporating BP nanosheets with a thermosensitive hydrogel composed of Poly(d,l‐lactide)‐poly(ethylene glycol)‐poly(d,l‐lactide) (PDLLA‐PEG‐PDLLA: PLEL) for postoperative cancer treatment. NIR irradiation mediated photothermal responsive of BP@PLEL hydrogel induced rapid sol–gel transition that enabled formation of a gelled membrane at the administration site. The sprayable BP@PLEL hydrogel demonstrated high PTT efficacy to eliminate residual tumor tissues in vivo at postoperative site and prevent the recurrence of cancer.^[^ ^290^ ^]^

### 5.2 Targeted Drug Delivery and Therapy

Recent strategies focus on the precision delivery of chemotherapeutic drugs to tumor site, critical for enhanced therapeutic efficacy.^[^ ^291^ ^]^ Designing of targeted delivery of drugs and therapy for bone cancers addresses two prime requirements: availability of drugs in the tumor microenvironment and limiting its adverse effect on healthy/normal cells. 2D materials‐based composites have been designed for osteosarcoma targeting using specific antibodies, siRNA/chemotherapeutic drug delivery, induce oxidative stress, and other strategies that demonstrate localized cytotoxicity against cancer cells while sparing normal cells.^[^ ^292^ ^]^ Further, to achieve the drug concentration in target tissues, designing strategies with predictable drug release kinetics and control over release is inevitable.^[^ ^293^ ^]^ The ultrahigh specific surface area of 2D materials promises application in drug delivery.^[^ ^140^, ^293^ ^]^ However, various methods of encapsulations including hydrogels have been chosen to prevent agglomeration of 2D materials, while improving biocompatibility and dispersion.^[^ ^293^ ^]^ The drug loading in 2D materials are mostly mediated by electrostatic interactions/secondary interactions that exhibits burst release, followed by sustained drug release through simple diffusion.^[^ ^294^ ^]^ The release can further be precisely controlled by irradiation mediated photothermal conversion in 2D materials that causes the delivery of loaded drug.^[^ ^140^, ^229^ ^]^ Various 2D materials incorporated hydrogels have been designed with efficient drug loading and predictable release kinetics to achieve burst, controlled or sustained drug release at targeted site.^[^ ^9^, ^215^, ^291^, ^295^ ^]^

Qiu et al.,^[^ ^291^ ^]^ developed a biodegradable drug delivery system with the concept of light activation of BP incorporated in agarose hydrogel for cancer therapy. The system uses photosensitizer that converts light into heat and melts the drug‐loaded hydrogel‐based nanostructures. The targeted drug release and kinetics can precisely be controlled by light parameters, BP, and hydrogel composition. The external NIR light excitation sufficiently penetrates deeper and ensures precision cancer therapy.^[^ ^291^ ^]^ Sustained delivery of DOX drug was achieved using a nanogel consisting of sodium alginate‐modified graphene oxide (SA‐GO) and N‐isopropyl acrylamide (NIPAM).^[^ ^296^ ^]^ The designed PNIPAM/SA‐GO nanogel system exhibited an efficient drug entrapment and release, biocompatibility and anti‐cancer activity in vitro.^[^ ^296^ ^]^ Saravanabhavan et al.^[^ ^292b^ ^]^ developed GO/CS/siRNA complex nanoparticles by ionic gelation method for efficient pH‐based release of siRNA. The targeted release of siRNA at tumor pH exhibited cytotoxic effect on Saos‐2 and MG‐63 osteosarcoma cells. Further, the GO‐CS complex showed negligible inflammatory response in both RAW 264.7 cells and bone marrow derived macrophages.^[^ ^292b^ ^]^

Tao et al.^[^ ^295^ ^]^ designed a poly(d,l‐lactide)‐poly(ethylene glycol)‐poly(d,l‐lactide) (PLEL)‐based thermosensitive micellar nanocomposite hydrogel platform incorporated with ultrathin Ti_3_C_2_ MXene nanosheets as photothermal agent and vascular disrupting agent (combretastatin A4, CA4) for synergistic antitumor therapy. The nanocomposite hydrogel exhibited favorable PTT in the NIR‐II (1064 nm) biowindow with a photothermal conversion efficiency of 41.4% and sustained drug release. The micellar system demonstrated efficient cellular uptake and selectively kill tumor vascular endothelial cells. The in vivo investigations reveal the thorough elimination of solid tumors using nanocomposite micellar hydrogel. This strategy could emerge as a potential approach targeting the unique vasculature physiology that essentially sustains solid tumors.^[^ ^295^ ^]^

### 5.3 2D Materials‐Based Hydrogels for Theragenerative Applications

The persistence of malignant tumor cell remnants, coupled with the absence of bone tissue for integration or new bone formation, continues to present significant challenges, contributing to cancer relapse and treatment failures.^[^ ^297^ ^]^ Theragenerative biomaterial systems with ability of combining therapy and regeneration have emerged as an integrative strategy for enhancing osteosarcoma therapy and combating the aggressive malignancy while promoting bone regeneration.^[^ ^297^, ^298^ ^]^ These approaches are beneficial treatment strategies that promote new bone formation in the bone defects caused by cancers and associated surgical removal treatments. Most of 2D materials that were discussed in earlier sections can provide dual functionality of sequential therapy to treat cancer and sufficient bioactivity for bone regeneration.

Facile fabrication of a 3D nHA‐rGO hydrogel scaffold by simple self‐assembly has been reported.^[^ ^299^ ^]^ The 3D structure self‐assembled during the stirring process and were transformed into stable scaffolds by lyophilization and thermal reduction. The therapeutic efficacy of the scaffold with NIR light (808 nm) irradiation was confirmed against MG‐63 cells. Implantation of nHA‐rGO scaffolds at the tumor site, followed by remote irradiation with NIR, raised the temperature to 60 °C within 4 min. Subsequent irradiation resulted in decreased xenograft tumor size and halted further growth after PTT. Moreover, the scaffolds promoted adhesion, proliferation, and osteogenic mineralization of rat bone marrow stem cells (rBMSCs) in vitro, as well as promoted bone regeneration in rat cranial defects. Thus, the hydrogel‐based theragenerative scaffold demonstrated potential for bone cancer therapy and regenerating bone in large defects due to cancers.^[^ ^299^ ^]^

**Figure 16:**
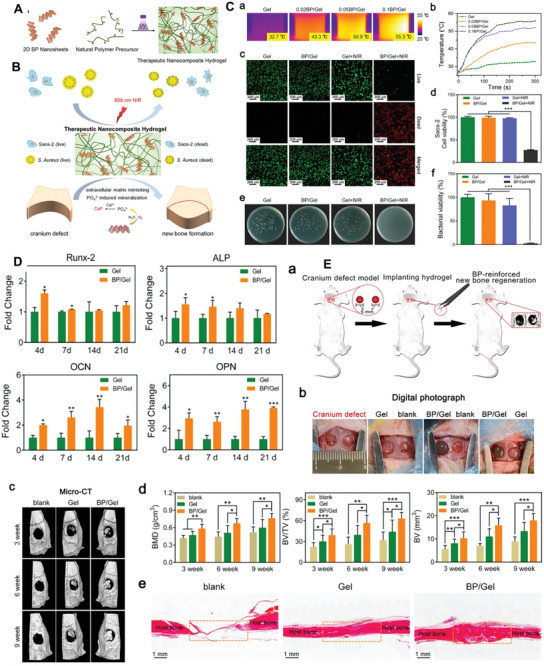
A) Schematic illustration of the preparation of therapeutic hydrogel from GelMA prepolymer and BP nanosheets. B) BP nanosheets have been incorporated into gelatin‐based matrix to generate therapeutic nanocomposite hydrogel (BP/Gel) with multiple functions including NIR photothermal osteosarcoma ablation, anti‐microbial activity, enhanced mineralization and osteogenesis in vitro and in vivo. Reproduced with permission.^[^ ^263^ ^]^ Copyright 2019, Royal Society of Chemistry. C) In vitro photothermal performance of the therapeutic hydrogels: (a) Infrared thermographic photographs of BP/Gel nanocomposite hydrogels irradiated by 808 nm NIR laser (1 W cm^−2^). (b) Photothermal heating curves of the nanocomposite hydrogels in vitro. (c) Live/dead staining assay of Saos‐2 cells on GelMA, BP/Gel, GelMA + NIR, and BP/Gel + NIR group. Live cells: green fluorescence, dead cells: red fluorescence) (scale bar, 200 µm). (d) CCK8 assay of Saos‐2 cells at different groups (*p < 0.05, **p < 0.01, ***p < 0.001). (e,f) Photothermal antibacterial performance: optical images and bacterial viability of *S. aureus* of GelMA, BP/Gel, GelMA + NIR, and BP/Gel + NIR group (*p < 0.05, **p < 0.01, ***p < 0.001). D) Osteogenic potential of therapeutic BP/Gel nanocomposite hydrogel.: (i) Gene analysis of hMSCs including Runx‐2, ALP, OCN, and OPN genes after hMSCs incubated for 4, 7, 14, and 21 days. E) In vivo osteogenesis performance (a) Schematic diagram of the establishment of SD rat cranium defect and hydrogel implantation. (b) The defect areas were implanted with pristine gelatin gel and BP/Gel for comparison. (c) Micro‐CT images of the harvested cranium obtained from SD rats at week 3, 6 and 9. (d) Quantitative analysis of the bone regeneration capability of BP/Gel hydrogel based on the micro‐CT histomorphometry, bone mineral density (BMD) showing the average bone density of the defect area, the bone volume/tissue volume (BV/TV) indicating the volume percentage of new born tissue over the entire defect and the bone mass (BV) indicating the specific new bone volume of the defect site (*p < 0.05, **p < 0.01, ***p < 0.001). (e) HE staining of tissue sections obtained from the rat cranial defects 9 weeks post‐treatment. Images were taken at two different magnifications. M represents the implant material, NB represents new bone, NV represents new vessels. The new bone formation in the central region of the cranial defects are marked in the images. The bone defects sections enclosed within the dotted lines are magnified in the bottom row images. Reproduced with permission.^[^ ^263^ ^]^ Copyright 2019, Royal Society of Chemistry.

BP based composite scaffolds have been designed to mediate PTT with NIR irradiation while simultaneously promote phosphorus‐driven biomineralization.^[^ ^263^, ^298^, ^300^ ^]^ Miao et al.,^[^ ^263^ ^]^ reported facile engineering of natural matrix with BP nanosheets to generate nanocomposite hydrogel (BP/Gel) (**Figure** **16**A,B).

The high surface area and charge of BP nanosheets strongly interacts with the gelatin that reinforces the UV mediated cross‐linking network (Figure 16A). The BP nanosheets incorporated into the natural matrix‐based nanocomposite BP/Gel hydrogel exhibited an array of therapeutic functions including near infrared (NIR) photothermal performance, anti‐bacterial activity, enhanced mineralization and osteogenesis in vitro and in vivo (Figure 16B). Under NIR irradiation, the temperature of the BP nanocomposite hydrogels increased from 27.4 to 43.3 °C and reduced the viability of osteosarcoma cells (Saos‐2) in vitro by PTT, which is attributed to the presence of BP (Figure 16C). The BP/Gel hydrogel group, on NIR laser irradiation showed lower number of *S. Aureus* bacterial colonies compared to hydrogel without exposure to irradiation. The photothermal efficiency of BP/Gel on NIR mediated irradiation, kills more than 98% of bacteria, thereby remarkable to prevent infections in osteosarcoma treatment (Figure 16C). Figure 16D reported the osteogenic‐related genes expression such as Runx‐2, ALP, OCN, and OPN in human mesenchymal stem cells (hMSCs) cultured in BP/Gel hydrogel. The late sign of osteogenic differentiation and mineralization processes, OCN and OPN genes, expression were significantly higher in BP/Gel hydrogel.^[^ ^263^ ^]^ The in vivo osteogenic regenerative potential has been investigated in a rat cranial defect model (Figure 16E). The new bone coverage was higher in BP/Gel nanocomposite hydrogel implanted group at 3, 6, and 9 weeks, represented in the Figure 16E.^[^ ^263^ ^]^ The quantitative histomorphometric analysis of micro‐CT images reported higher values of bone mineral density (BMD), bone mass/tissue volume (BV/TV) and bone mass (BV) in BP/Gel nanocomposite hydrogel, after 6 and 9 weeks of implantation. Similarly, Hematoxylin and Eosin (HE) staining evaluated prominent bone formation in BP/Gel hydrogel group after 9‐week post‐surgery. Thus, the BP/Gel nanocomposite hydrogel demonstrated an effective theragenerative potential for bone cancer therapy and subsequent bone regeneration at the implant site.^[^ ^263^ ^]^

In a recent study, Li et al.,^[^ ^12b^ ^]^ encapsulated BP nanosheets (BPNS) and DOX in an injectable chitosan‐based hydrogel (BP/DOX/CS). The BP/DOX/CS hydrogel exhibited good compatibility and MC3T3‐E1 cells differentiation, attributed to the phosphate release. The temperature of BP/DOX/CS rose up to 56.3 °C, sufficient to cause tumor ablation. The temperature of BP/DOX/CS increased rapidly within 5 min and reached 51 °C, after NIR irradiation in mouse model. The hydrogel effectively combined the PTT and chemotherapy by continuous release of DOX, eliminating the K7M2‐WT tumor cells. Anti‐tumor efficacy in vivo revealed efficient elimination of tumor without systemic toxicity. This multi‐modal therapeutic and regenerative approach is advantageous as a localized and injectable strategy for osteosarcoma management.^[^ ^12b^ ^]^

**Figure 17:**
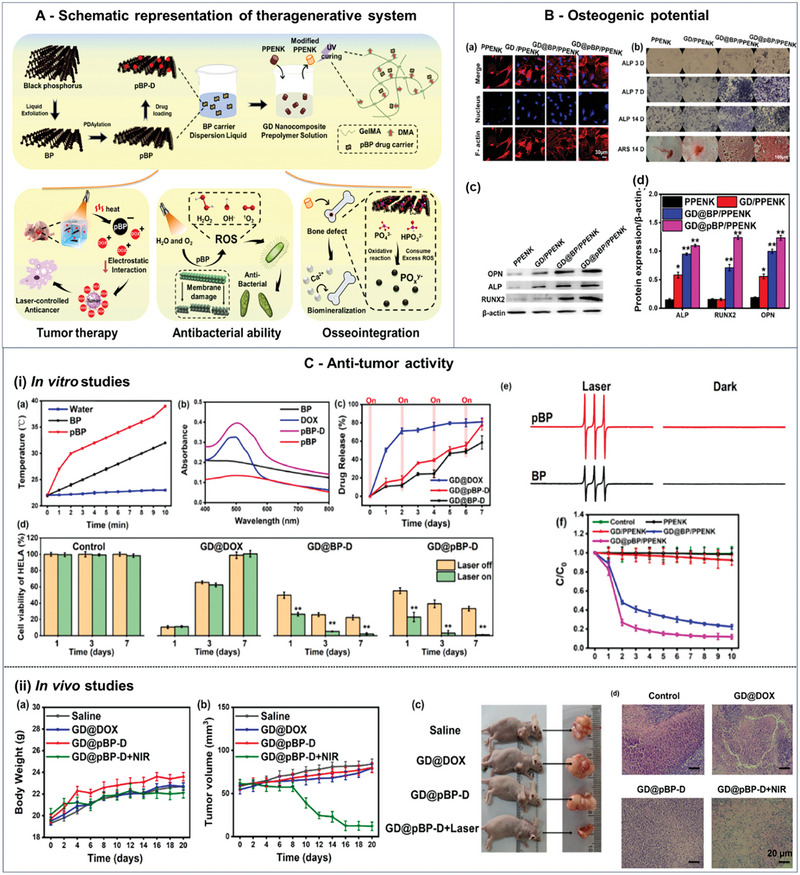
A) Schematic illustration of the preparation of GelMA/DMA nanocomposite photo‐cross‐linking hydrogel coated on PPENK implant for tumor therapy, antibacterial activity, and osseointegration. B) In vitro osteogenic ability: (a) Fluorescence images of MC3T3‐E1 cells stained with TRITC‐phalloidin (red) and DAPI (blue) (scale bar, 30 µm). Osteogenic differentiation of MC3T3‐E1 cells by (b) ALP staining on 3, 7, 14 days and alizarin red staining on 21 days (scale bar, 100 µm). (c) Western blot detection and (d) quantitative analysis of OPN, ALP, and RUNX2 protein levels in each group. C) Antitumor activity: (i) Photothermal performance‐controlled DOX release in vitro. (a) Photothermal heating curves on irradiation with an NIR laser (808 nm, 1.0 W cm^−2^) for 10 min. (b) UV–vis absorption spectra of DOX, BP, pBP, and pBP‐D. (c) Laser‐controlled DOX release from different samples in 7 days. (d) Cell viability of HELA cells incubated with different samples under laser off/laser on (808 nm, 1 W cm^−2^, 10 min per day) for 1, 3, and 7 days. (e) EPR spectra of ^1^O_2_ generated by BP and pBP under dark and laser stimulation. (f) Comparative trend of C/C_0_ of DPBF under laser stimulation. C represented the OD value at different times, C_0_ represented the initial OD value. (ii) In vivo studies: (a) Body weight of mice recorded every other day. (b) Corresponding growth curves of tumors in mice with different treatments. (c) Images of representative tumor in mice with different treatments. (d) Histological micrographs of tumor tissues stained with H&E after the treatments on day 21. Scale bar, 20 µm. Reproduced with permission.^[^ ^294^ ^]^ Copyright 2023, Elsevier B.V.

Jian and colleagues reported hydrogel designed by incorporation of 2D BP modified by polydopamine (pBP), into photo‐cross‐linkable gelatin methacrylate/dopamine methacrylate hydrogel coating the phthalazinone (PPENK) and poly (aryl ether nitrile ketone) construct for anti‐tumor therapy and bone regeneration (**Figure** **17**A).^[^ ^294^ ^]^ The multi‐functional construct exhibited NIR laser (808 nm) irradiation mediated photothermal release of anti‐cancer drug doxorubicin hydrochloride, PDT and ROS generation to combat bacterial infection (Figure 17A). The osteogenic potential using MC3T3‐E1 cells (Figure 17B), showed improved cell adhesion, cell spreading and fluorescence intensity of F‐actin in the hydrogels with BP and pBP compared to PPENK coated hydrogels. The GD@pBP/PPENK exhibited significantly enhanced expression of OPN and RUNX2 indicating mineralization potential in vitro. Further, the ALP staining and alizarin red staining showed ALP content and calcium deposition in the cells on GD@BP/PPENK and GD@pBP/PPENK compared to PPENK and GD/PPENK. Finally, in consistent with gene expression, the western blot depicted enhanced expression of ALP, OPN, and RUNX2 in GD@BP/PPENK and GD@pBP/PPENK attributed to the release of BP and pBP (Figure 17B).^[^ ^294^ ^]^ On NIR (808 nm) irradiation, the photothermal efficiency of pBP (ΔT = 16.1 °C) was improved compared to bare BP (ΔT = 9.8 °C), attributed to polydopamine modification. The chemotherapeutic drug DOX was efficiently loaded in BP and pBP, characterized by a slight red shift in UV–vis spectra. The controlled release of the loaded DOX were reported on NIR exposure for 10 min (on) at 1, 3, 5, and 7 days, while alternating with laser off cycles rest of the time (Figure 17C‐i).^[^ ^294^ ^]^ The system reported laser stimulation based controlled drug release kinetics mediated by photothermal efficiency. As the temperature rises, the electrostatic attraction weakens thereby releasing the drug in a controlled fashion.^[^ ^294^ ^]^ The anti‐tumor effect of nanocomposite hydrogel was reported using HELA cells and controlled drug release on laser irradiation. The controlled drug release from GD@BP‐D and GD@pBP‐D mediated by laser on/off cycles reduced viability significantly, attributed to the sustained the cytotoxic drug effect for 7 days (Figure 17C‐i).^[^ ^294^ ^]^ Figure 17C‐ii showed the in vivo anti‐cancer efficiency of nanocomposite coated hydrogels in tumor‐bearing mice, upon NIR laser irradiation‐based DOX release. The study reported tumor size reduction, cancer cells death and apoptosis.^[^ ^294^ ^]^ In addition, the fabricated mussel inspired hydrogel coated multi‐functional implant promoted osteointegration in SD rat femoral condyle defect model.^[^ ^294^ ^]^

Yin et al.^[^ ^223^ ^]^ devised a combined approach by integrating anti‐bacterial potential with the osteosarcoma ablation activity in a multifunctional implant that mainly consists of MXene nanosheets, GelMA hydrogels, and bioinert sulfonated polyetheretherketone (SP). The tobramycin loading displayed anti‐bacterial properties against gram‐negative/gram‐positive bacteria, while NIR irradiation (808 nm) mediated synergistic photothermal effects of MXene and polydopamine killed osteosarcoma cells. The 3D porous multifunctional implant demonstrated osteogenesis, mineralization and in vivo osseointegration.^[^ ^223^ ^]^ In a recent study, a dual‐functional theragenerative 3D composite scaffold from hybridization of photo‐cross‐linked silk fibroin (SF) biopolymer with MXene (Ti_3_C_2_) nanosheets have been developed.^[^ ^301^ ^]^ The aerogel scaffold fabrication with controlled pore size, macroscopic geometry, and mechanical stability was achieved by 3D printing and photo‐cross‐linking of methacrylate‐modified SF (SF‐MA) self‐assembly‐driven hydrogel. MXene 2D nanosheets were integrated into the 3D printed scaffold to impart remotely controlled photothermal antiosteosarcoma ablation function. These composite scaffolds mediated the growth and proliferation of preosteoblast cells (MC3T3‐E1) and bone mineral deposition, ideal for bone regeneration. In addition, generated heat (45–53 °C) accelerated the localized release of anti‐cancer drug (sorafenib) and mediated photothermal ablation of cancer cells (MG‐63 cells).^[^ ^301^ ^]^

**Figure 18:**
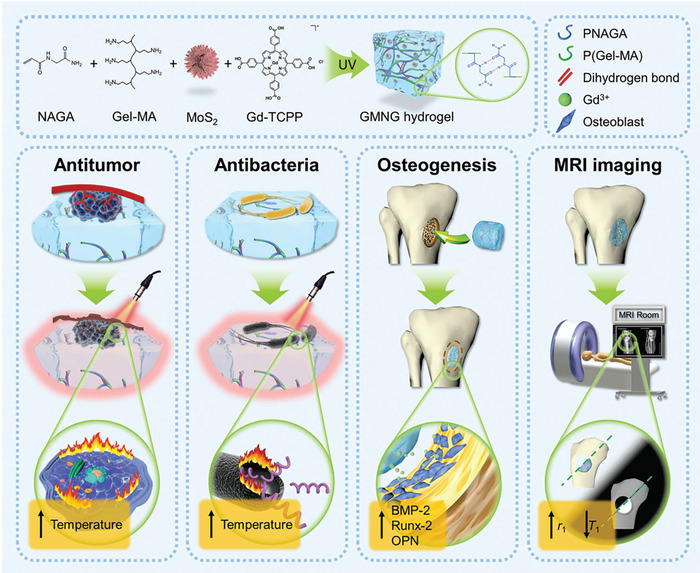
Schematic illustration of the preparation of (Gd)‐complex and molybdenum sulfide (MoS_2_) co‐doped N‐acryloyl glycinamide (NAGA)/gelatin methacrylate (Gel‐MA) (GMNG) hydrogel and its multifunctionality. Reproduced with permission.^[^ ^302a^ ^]^ Copyright 2023, Wiley‐VCH.

**Figure 19:**
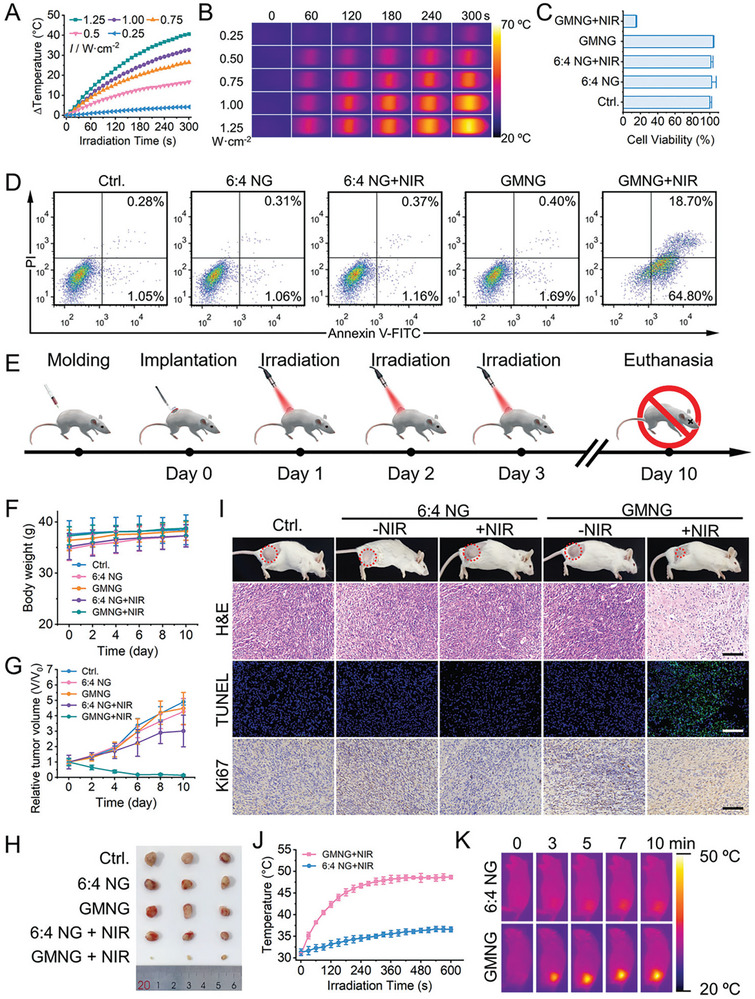
A) Temperature‐increase curves and B) infrared imaging photos of GMNG hydrogel after 808 nm NIR irradiation with different power densities. C) Cell viability of MG‐63 cells after different treatments, where NIR is an 808 nm laser of 1.25 W cm^−2^ for 300 s. D) Cell apoptosis of MG‐63 cells seeded on GMNG hydrogel after different treatments measured with Annexin/PI staining. E) Schematic illustration of in vivo antitumor study timeline. F) Time‐dependent body weight curves of mice with different treatments. G) Time‐dependent volume curves of solid tumors after different treatments. H) Photos of solid tumors from mice after different treatments. I) Photos of tumor‐bearing mice and H&E, TUNEL, and Ki67 staining results of tumors from mice after different treatments. Scale bar, 500 µm. J) Temperatures change curves of solid tumor sites under 808 nm NIR irradiation (1.0 W cm^−2^, 10 min). K) Real‐time infrared thermal images of the tumor‐bearing mice (implanted with 6:4 NG or GMNG hydrogel) under 808 nm NIR irradiation (1.0 W cm^−2^, 10 min). Reproduced with permission.^[^ ^302a^ ^]^ Copyright 2023 Wiley‐VCH.

Molybdenum sulfide (MoS_2_), a well‐known 2D TMDC has good photothermal conversion efficiency and are explored for cancer therapy and imaging.^[^ ^302^ ^]^ Huang et al.,^[^ ^302a^ ^]^ demonstrated the multifunctional potential of a gadolinium (Gd)‐complex and MoS_2_ co‐doped N‐acryloyl glycinamide (NAGA)/GelMA hydrogel (GMNG). The multi‐functional GMNG hydrogel exhibited excellent PTT to kill tumor cells and anti‐bacterial activity, osteogenesis, and enhanced contrast for magnetic resonance imaging (MRI) (**Figure** **18**).^[^ ^302a^ ^]^ The Gd‐complex and Gd^3+^ release from hydrogel imparted MRI imaging performance and osteogenic properties to the GMNG hydrogel. The photothermal conversion efficiency of MoS_2_ nanoroses conferred the hydrogel system excellent PTT to combat cancer recurrence and bacterial infection both in vitro and in vivo. On NIR irradiation, the temperature increased due to PTT, the reduction in MG‐63 cells viability revealed the anti‐tumor potential (**Figure** **19**) in vitro and tumor size reduction in an in vivo mice model. Additionally, bone defect repair ability using GMNG hydrogel was demonstrated in SD rat tibia defect model.^[^ ^302a^ ^]^ The addition of MoS_2_ nanoroses in the GMNG hydrogel showed reliable photothermal ability in vitro (Figure 19A,B). The GMNG hydrogel with and without NIR cultured with MG‐63 cells showed promising cellular activity, which was similar to the control group. However, NIR irradiation of GMNG hydrogel decreased the cell viability of MG‐63 cells on GMNG hydrogel lower than 20% (Figure 19C). The quantitative flow cytometric analysis (Figure 19D) showed the higher apoptosis rate of GMNG+NIR‐treated cells compared to GMNG without NIR, thereby confirmed the effective PTT of GMNG hydrogel for tumor in vitro. The photothermal ability of GMNG hydrogels against cancer in vivo, was explored in tumor model established in Kunming mice (Figure 19E). There was no significant difference in body weights of animals in hydrogels implanted groups with and without NIR radiation (Figure 19F). The tumor volume evidently decreased in GMNG hydrogel groups treated with 808 nm NIR (GMNG (+NIR)), while increased in other groups (Figure 19G). The volume of tumors collected surgically from the GMNG (+NIR) group, was smaller than other groups, thereby confirmed the PTT on tumor in vivo (Figure 19H,I). Further, the H&E, TUNEL, and Ki67 staining demonstrated the antitumor mechanism of GMNG hydrogel in vivo, after photothermal tumor‐ablation (Figure 19I). The H&E staining showed less blue/purple color (nuclei) in the GMNG (+NIR) group, that implied higher number of apoptotic osteosarcoma cells in the GMNG (+NIR) group than in other groups (Figure 19I). In GMNG (+NIR) group, the highest TUNEL immunofluorescence intensity, while the least proliferation of cancer cells (dark spots of Ki67 stained image) were reported, which implied highest level of apoptosis and the least active proliferation of tumor cells compared to other groups (Figure 19I). Besides, the temperature at tumor sites implanted with GMNG hydrogels and irradiated were increased to ≈48 °C after irradiation for 10 min, which would kill the tumor cells sparing the surrounding tissues (Figure 19J,K).^[^ ^302a^ ^]^ Therefore, the system emerges as a unified therapeutic‐prophylactic strategy that prevents tumor recurrence infection and promotes bone remodeling.^[^ ^302a^ ^]^ These 2D materials‐based nanocomposite hydrogels are emerging as advanced strategies with multi‐functional translational potential for bone theragenerative systems.

### 5.4 Synergistic Therapy

The multi‐faceted feature of 2D materials in combination with hydrogels have been designed for generating multi‐functional systems that integrates different therapeutic strategies such as synergistic or combination therapy.^[^ ^295^, ^303^ ^]^ The synergistic effects in combined therapies result in improved tumor destruction and simultaneously reduce the dosage of chemotherapeutic drugs and adverse effects on healthy tissues. Moreover, 2D materials can be used for immune‐mediated cancer therapy by the stimulation of the immune system, leading to the release of a large number of antigens to provide a strong anti‐cancer immune reaction and prevent tumor recurrence or metastasis.

The 2D materials hydrogels have been designed to deliver chemotherapeutic drugs and simultaneously enable PTT/PDT which in turn serves as adjuvant therapy.^[^ ^295^, ^303^ ^]^ These combined systems promise a robust approach to resurrect cancer and combat recurrence. Xing et al.,^[^ ^303b^ ^]^ developed hydrogel nanoplatforms, based on cellulose, MXene Ti_3_C_2_ nanosheets, and the chemotherapeutic drug DOX, for dual‐modality photothermal/chemotherapy against cancer. On NIR (808) irradiation, the MXene/DOX/cellulose composite hydrogel exhibited PTT and dynamic release of DOX. The system exhibited a localized rise in temperature to >40 °C that rendered cancer cells more susceptible to chemotherapeutic drug. The decrease in cancer cells viability in vitro and tumor size reduction in vivo demonstrated the synergistic therapeutic potential of the developed nanocomposite hydrogel platform.^[^ ^303b^ ^]^

**Figure 20:**
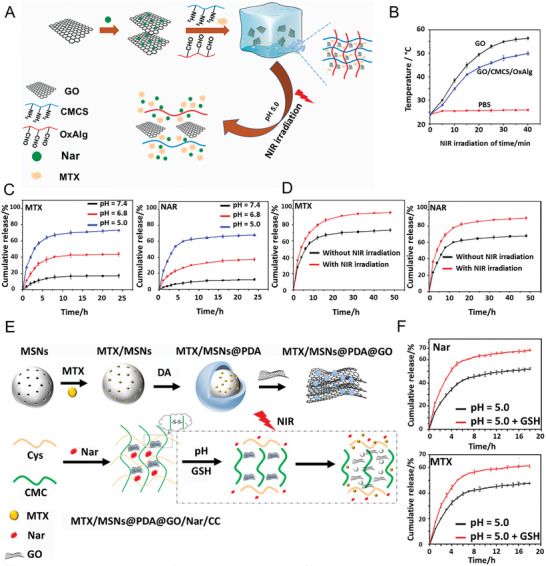
A) Schematic illustration of the dual‐responsive DDDS preparation. B) Photothermal curves of 0.1 m PBS (5 mL, pH 5.0) dispersed with GO and GO/CMCS/OxAlg of 100 µg mL^−1^ under NIR irradiation. C) Release curves of MTX and Nar from the DDDS in PBS (50 mL, 0.1 m) with different pH, and D) with and without NIR irradiation. Reproduced with permission.^[^ ^307^ ^]^ Copyright 2022 Elsevier Ltd. All rights reserved. E) Schematic illustration of a pH, redox, and the NIR‐responsive DDDS system. F) Release curves of Nar and MTX in 0.1 m PBS of pH 5.0 without and with 10 mm GSH. Reproduced with permission.^[^ ^308^ ^]^ Copyright 2023, Elsevier B.V.

Graphene and GO are frequently explored in combined therapies due to their desirable photothermal efficiency, leading to tumor cell ablation, the release of drugs, and increased cellular uptake.^[^ ^304^ ^]^ However, PTT alone may be insufficient to induce thermal damage to deeply situated cancer cells or to eradicate metastatic cells due to its limited depth of tissue penetration.^[^ ^305^ ^]^ Dual‐drug delivery systems (DDDS) can be a promising approach benefiting from the synergistic effects of two drugs with different therapeutic effects, which can enhance the anticancer effect of other strategies and reduce the side effects of chemotherapeutic drugs.^[^ ^306^ ^]^ In this regard, to induce apoptosis of cancer cells in osteosarcoma, naringin (NAR) as an active ingredient of Rhizoma Drynariae total flavonoids was loaded onto graphene oxide (GO) through *π*–*π* stacking and hydrogen bonding, and then co‐encapsulated with methotrexate (MTX) in a covalently cross‐linked hydrogel based on carboxymethyl chitosan (CMCS) and oxidized alginate (OxAlg) that was synthesized by Schiff base reaction (**Figure** **20**A).^[^ ^307^ ^]^ The aldehyde (–CHO) groups of OxAlg could cross‐link with the amino (─NH_2_) groups of CMCS through the formation of acylhydrazone (─N═C─) bonds that can be hydrolyzed in an acidic medium, providing the pH‐responsive delivery of Nar and MTX from the prepared platform (Figure 20C). The photothermal activity of the developed DDDS under NIR irradiation was observed due to the photothermal conversion capacity of GO that can cause the ablation of tumor cells and simultaneously lead to faster diffusion of MTX from the DDDS due to the produced hyperthermia (Figure 20B,D). Therefore, this developed system can be considered a promising candidate for effective treatment of osteosarcoma. In another attempt with the same concept, a pH, redox, and the NIR‐responsive DDDS system was constructed for chemo/photothermal therapy of osteosarcoma (Figure 20E).^[^ ^308^ ^]^ MTX was loaded into the mesoporous silica nanoparticles (MSNs), which were then encapsulated by PDA to fabricate a pH‐ and NIR irradiation‐responsive drug delivery carrier. MSN can provide a promising carrier for the controlled delivery of drugs due to rich mesopores. PDA could be firmly attached to the surface of MSNs, providing outstanding photothermal activity and high pH sensitivity. Then, the core‐shell structured MTX/MSNs@PDA was embedded into the nanosheets of GO to improve its photothermal conversion capability. Subsequently, the prepared MTX/MSNs@PDA@GO was co‐encapsulated with Nar into the hydrogels of CMC and cystamine (Cys) generated through an amidation reaction. Therefore, in addition to PDA, the amide linkage in the CMC/Cys (CC) hydrogels provides pH sensitivity that leads to pH‐responsive delivery of Nar and MTX from the DDDS. Furthermore, the –S–S– linkage in Cys can be reduced to sulphydryl (–SH) by glutathione (GSH), causing the redox‐responsive delivery of two drugs and the degradation of the hydrogels (Figure 20F). NIR irradiation‐responsive delivery of Nar and MTX from the DDDS could also be achieved due to the excellent photothermal effect of both PDA and GO. This would allow chemo‐photothermal therapy of osteosarcoma.

**Figure 21:**
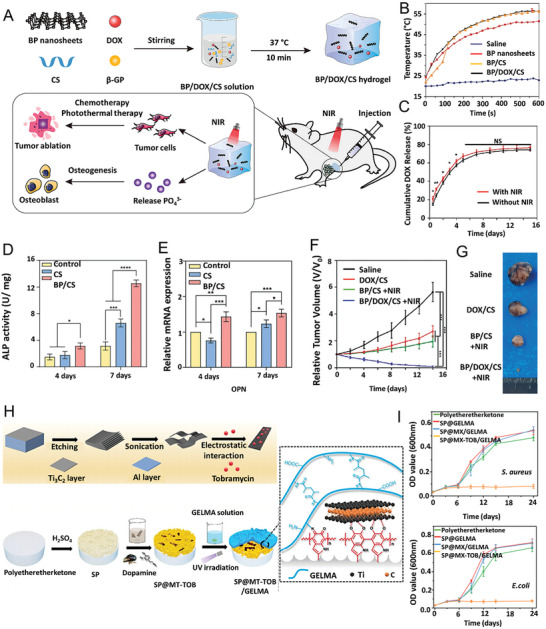
A) Schematic illustration of the preparation of the injectable BP/DOX/CS hydrogel for synergistic photothermal‐chemotherapy of osteosarcoma and osteogenesis. B) Photothermal curves of different preparations under 10 min NIR irradiation. C) Cumulative release curves of DOX in BP/DOX/CS hydrogel with and without NIR irradiation (*p < 0.05, **p < 0.01, NS, no significance). D) ALP activity and E) expression of OPN at days 4 and 7 (*p < 0.05, **p < 0.01, ***p < 0.001, ****p < 0.0001). F) Relative tumor volume curves of different treated groups. G) Photograph of dissected tumors of different treated groups at the end of the experiment. Reproduced with permission.^[^ ^12b^ ^]^ Copyright 2023, Elsevier B.V. All rights reserved. H) Schematic illustration of the preparation of the multifunctional SP@MX‐TOB/GelMA. I) Bacterial growth curves of *S. aureus* and *E. coli* after treatment with different groups. Reproduced with permission.^[^ ^223^ ^]^ Copyright 2020, American Chemical Society.

Besides these promising platforms for the treatment of bone cancer, it is worth noting that tumor resection causes organ dysfunction and poor quality of life.^[^ ^309^ ^]^ Therefore, the integration of tumor ablation with osteogenesis‐promoting capabilities is highly preferred in the treatment of osteosarcoma. Moreover, reducing the toxicity of drugs is an absolute necessity for promoting osteogenesis. The presence of 2D BP nanosheets in the platforms meaningfully can reduce the long‐term toxicity phenomenon of released DOX during bone regeneration as well as possessing high‐photothermal‐conversion efficiency for efficient removal of tumor, favorable biocompatibility, and controlled biodegradability. In contrast, graphene or GO cannot degrade in vivo.^[^ ^298^, ^310^ ^]^ Considering that, the hydrophobic BP nanosheets as a photothermal agent, low dose of hydrophilic DOX as an anti‐cancer drug, and high dose of the hydrophilic osteogenic peptide as an osteogenic factor, as well as β‐tricalcium phosphate (β‐TCP) nanoparticles, were incorporated in a hierarchical porous nanocomposite scaffold that was cryogenically 3D‐printed to provide synergistic effect of chemotherapy and PTT to eliminate cancer cells at first.^[^ ^298e^ ^]^ Then the continuous release of osteogenic peptides from the 3D‐printed hydrogel led to bone defect reconstruction. Beyond the effective photothermal effect of BP nanosheets, the distinct bioactive phosphorus‐based chemotherapy effect was also observed by BP due to the induction of G2/M phase arrest causing apoptosis‐ and autophagy‐mediated cell death in different cancer cells, but not in normal cells. This suggests the meaningful role of BP nanosheets in future advanced anti‐cancer therapies by combining photothermal therapy and chemotherapy.^[^ ^311^ ^]^ In a different study, Li et al. developed an injectable multifunctional hydrogel for synergistic photothermal chemotherapy of osteosarcoma followed by osteogenesis. For this purpose, BP nanosheets and DOX were encapsulated in an injectable physically cross‐linked thermosensitive hydrogel consisting of CS and β‐glycerophosphate (β‐GP) (**Figure** **21**A).^[^ ^12b^ ^]^ The presence of BP nanosheets in the hydrogel increased the temperature to ≈56 °C under NIR irradiation for 10 min, which is sufficient for tumor ablation, and it was lower than the temperature of BP nanosheets when dispersed only in water because the encapsulation of BP nanosheets in hydrogel reduces heat diffusion (Figure 21B). Moreover, the NIR irradiation enhanced the initial release of DOX, which is due to the degradation of the polymer matrix caused by local hyperthermia, resulting in the quick and effective therapeutic response (Figure 21C). The expression of ALP as a marker to evaluate early osteogenic differentiation of osteoblast precursors and the expression of osteogenic‐related genes including OPN, RUNX2, and BMP‐2 was the highest in BP/CS group, which confirmed that the prepared platform could improve osteogenic differentiation and mineralization in vitro (Figure 21D,E). The osteogenic activity of the fabricated hydrogel can be associated with the upregulating effect of CS on expression levels of osterix and bone sialoprotein. In addition, β‐GP itself can cause mineral deposition. Moreover, BP nanosheets can easily degrade to nontoxic phosphate in the interaction of oxygen and water. Subsequently, phosphate ions can enhance calcium capture to form calcium phosphate (CaP) deposition and promote bone regeneration. It is important to note that BP nanosheets under NIR irradiation could enhance osteogenesis both in vitro and in vivo by upregulating the heat shock protein (HSP) expression. The synergistic PTT and chemotherapy of osteosarcoma were observed under NIR irradiation using this platform (Figure 21F,G), which provides outstanding potential for treating bone‐related tumors.

Besides BP, other 2D Xenes have also been utilized for PTT of bone cancers in combination with other strategies.^[^ ^304^ ^]^ Following the surgical removal of osteosarcoma, the health of patients remains at risk due to cancer recurrence, postoperative infections, and large bone loss.^[^ ^312^ ^]^ To overcome these challenges, Yin et al. prepared a novel multifunctional hydrogel that contains MXene nanosheets, tobramycin (TOB), PDA, GelMA hydrogels, and bioinert sulfonated polyetheretherketone (SP) to achieve tumor ablation, antibacterial activity, and osteogenesis (Figure 21H).^[^ ^223^ ^]^ Ti_3_C_2_Tx‐based MXenes show high photothermal conversion efficiency that can be useful for PTT of cancer. Moreover, Ti_3_C_2_Tx‐based MXenes have large surface areas alongside plenty of negatively charged groups that enable the affinity for positively charged antibacterial drugs like tobramycin (TOB) through strong electrostatic interactions. Therefore, a coating consisting of TOB‐laden MXene nanosheets and GelMA hydrogels was fabricated on SP. On the other hand, GelMA hydrogels accelerate bone regeneration due to the existence of inherent cell attachment‐promoting arginine−glycine−aspartic acid (RGD) sequences. Moreover, polyetheretherketone is considered a promising polymer for orthopedic implants due to the similar Young's modulus to human cortical bone. PDA as a photothermal agent was also applied, which can chelate with Ti elements of MXenes to improve photothermal function. The released TOB causes antibacterial activity by preventing mRNA from being translated into protein and enhances the germicidal properties of Ti_3_C_2_, which suppresses bacterial growth through contact killing (Figure 21I). Considering these results together, the fabricated multifunctional hydrogel revealed a synergistic effect for the treatment of postoperative bone loss after osteosarcoma resection.

**Figure 22:**
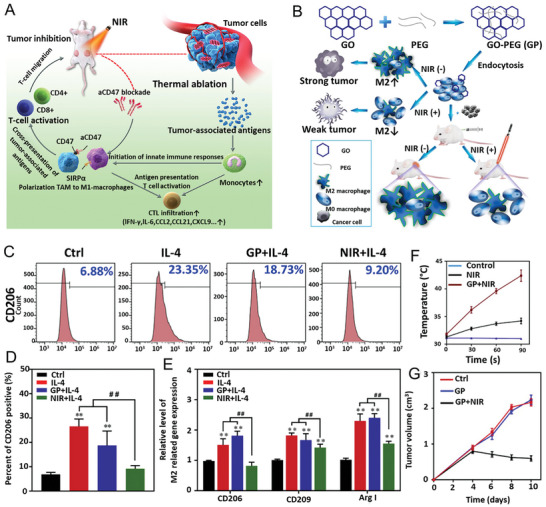
A) The schematic illustration of the proposed mechanism of anti‐tumor immune responses induced by the combination of photothermal therapy with aCD47. Reproduced with permission.^[^ ^315^ ^]^ Copyright 2020, The Author(s). B) The schematic illustration of the effect of photothermal therapy on macrophage polarization and tumor cells. C,D) Flow detection of cell surface marker CD206 of different groups and corresponding statistical results after NIR irradiation for the 90 s (808 nm NIR and 0.7 W cm^−2^). E) Gene expression of CD206, CD209, and Arg I. The error bars indicate means ± SD and *N* = 3: **p < 0.05 compared with the control group, ##*p* < 0.01 compared with IL‐4 and GP+IL‐4 group. F) Temperature curve of different treated groups. G) Tumor volume of different groups. Reproduced with permission.^[^ ^318^ ^]^ Copyright 2020, Elsevier B.V. All rights reserved.

2D materials can also be considered as ideal carriers of immune regulators. For example, GO and rGO have been explored as promising vaccine adjuvants to enhance the immune response against antigens, aiming to improve cancer treatment outcomes. For this purpose, Yue et al. developed a platform by combining GO with ovalbumin (OVA, a protein antigen that can elicit cellular and humoral immune responses for cancer immunotherapy) to investigate how this combination could trigger immunological response against cancer.^[^ ^313^ ^]^ After vaccination, the GO triggered the production of cytokines followed by attracting a significant number of antigen‐presenting cells (APCs) to the injection sites. After cellular uptake, the flat GO was inclined to become a folding shape in the APCs, which can induce a process called autophagy and act as a reservoir for the antigen. Consequently, it led to the activation of specific CD8 T cells in vivo. Moreover, the GO‐OVA vaccine demonstrated a superior anti‐tumor effect and significant reduction of tumor size compared to the OVA‐treated group. These results highlight that the utilization of GO as an adjuvant resulted in increased cytotoxicity against tumor cells and enhanced immunological responses expressing the specific antigen, ultimately leading to the rejection of the tumors. Another study indicated that PEGylated graphene oxide (GOP) triggered a time‐ and dose‐dependent immune response in macrophages.^[^ ^314^ ^]^ At higher doses with a single injection, GOP significantly amplified the immune response, suggesting its potential as an adjuvant for immunotherapy. Therefore, such 2D materials can be used as multifunctional platforms for combining immunotherapy with other cancer therapeutic methods to enhance the therapeutic efficiency against cancer. For example, Xie et al. performed a study to synergistically enhance cancer treatment by the combination of BP‐based photothermal therapy (PTT) with anti‐CD47 antibody (aCD47)‐based immunotherapy (**Figure** **22**A).^[^ ^315^ ^]^ PTT using BP displays a dual impact by directly eliminating tumor cells and recruiting increased levels of monocytes to the tumor site, triggering the innate immune responses as well as inducing the release of tumor‐specific antigens from destroyed cancer cells to trigger cytotoxic T lymphocytes‐mediated adaptive immunity.^[^ ^316^ ^]^ CD47, a protein expressed in human cells, is notably elevated in various tumor cells. When it binds with signal regulatory protein‐alpha (SIRPα) expressed on macrophages, effectively blocks the phagocytosis process by macrophages, which extends to both innate and adaptive immunity. aCD47 disrupts the CD47/SIRPα axis and shows promising efficacy against different cancers. However, simply blocking the SIRPα‐CD47 interaction cannot induce an adequate antitumor immune response.^[^ ^317^ ^]^ Therefore, combining BP‐induced PTT with aCD47 not only suppresses cancer cell growth more effectively but also alters macrophage behavior toward a pro‐immune response, enhancing the destruction of tumor cells and potentially inhibiting their growth at distant sites, showing promise in preventing metastatic cancers and cancer recurrence. Taken together, this combined therapy triggers both local and systemic immune reactions against cancer. In another study, Deng et al. assessed the effect of NIR light irradiation on the anti‐tumor capabilities of macrophages using GO in combination with PEG (GP) as a photothermal material (Figure 22B).^[^ ^318^ ^]^ Loading PEG helps GO to reach the desirable biocompatibility. To assess the polarization of macrophages, the RAW264.7 macrophage cell line was seeded in 24‐well plates and exposed to different groups. The results showed increased levels of the surface protein CD206, a marker protein for M2 polarization after treatment with the IL‐4 group. However, the NIR+IL‐4 group exhibited decreased levels of CD206, indicating that PTT can weaken IL‐4‐induced M2 polarization of macrophages and reduce the expression of M2‐related genes, including CD206, CD209, and Arg I (Figure 22C–E). This could consequently reduce the migration and invasion abilities of osteosarcoma HOS cells, and with efficient heat generation, finally led to the reduction of tumor size (Figure 22F,G).

These findings highlighted the potential of PTT in hindering osteosarcoma invasion by modulating M2 polarization, offering a promising strategy against tumor progression. However, these approaches can be optimized by the integration of 2D materials into hydrogel matrices to benefit from advantages such as improved drug loading and targeted drug delivery, controlled release, higher retention time of therapeutic agents in the tumor site, and biocompatibility, while minimizing off‐target effects. This can potentially enhance the synergistic effects observed in the combination therapy and provide advancements in combating cancer.

## 6 Applications of 2D Materials‐Based Hydrogels for Bone Infections

Osteomyelitis refers to bone infections that can arise from open injury to bone and its surrounding soft tissues.^[^ ^319^ ^]^ Additionally, such infections can be caused by infected implants (bone implant‐associated infection) or can occur during orthopaedic surgery, which is susceptible to pathogenic bacterial infections.^[^ ^320^ ^]^ Osteomyelitis is typically caused by bacteria, and *Staphylococcus sepsis* and *Staphylococcus epidermidis* are responsible for many cases.^[^ ^321^ ^]^

Currently, anti‐infective therapy through administration of antibiotics and other antibacterial agents (e.g., peptides and proteins) is the primary treatment strategy of osteomyelitis. Despite the success rate of this method, it is associated with many challenges namely: i) long‐term administration of antibiotics is costly; ii) the efficacy of antibiotics has diminished over time due to the gradual emergence of bacterial resistance; iii) some antibacterial agents such as the metal‐based ones have insufficient biocompatibility; and iv) overuse and high‐dose of antibiotics have detrimental side effects^[^ ^322^ ^]^}. Along with the anti‐infective therapy, surgical management is also often essential for removal of pathogens, their biofilms, and the dead bone.^[^ ^323^ ^]^ Nevertheless, surgery requires multiple procedures which can result in considerable costs, extended periods in hospitals, lengthy antibiotic administrations, and even biomechanical changes.^[^ ^324^, ^325^ ^]^

Among various strategies for the management of osteomyelitis (e.g., surgery and administration of antibiotics), hydrogel‐based therapies have received great attention, mainly due to the design customization, operational simplicity, and precision of hydrogels in the treatment of bone infections.^[^ ^326^ ^]^ Combined with unique features of 2D materials, namely surface functionalization feasibility, good biocompatibility, excellent photoluminescence property, photostability, and photo‐to‐heat conversion property, hydrogels based on 2D materials can play an important role in effective bone infection therapeutics.^[^ ^327^ ^]^ Given this background, in the following sections, various applications of hydrogels based on 2D materials for antibiotic delivery, biofilm disruption, photothermal therapy, and imaging for bone infection therapeutics purposes are discussed.

### 6.1 Antibiotic Drug Delivery

2D materials demonstrate unique advantages in both endogenous and exogenous antibacterial strategies due to their specific 2D structure and properties. They offer large surface area which is beneficial for drug delivery. Furthermore, they can utilize their 2D structures to physically puncture microorganisms, providing an effective alternative to traditional antibacterial methods.^[^ ^328^ ^]^ 2D materials can also facilitate the loading and release of inhibitors which is crucial for bone formation. For instance, Ou et al. showed that the miR‐214 inhibitor can be loaded into polyethyleneimine (PEI) with the help of graphene oxide (GO). Through formation of a shell, controlled release of the inhibitor was achieved. The delivery approach of GO‐PEI‐miR‐inhibitor was shown to offer a combination of effective transfection and adjustable release duration.^[^ ^329^ ^]^

**Figure 23:**
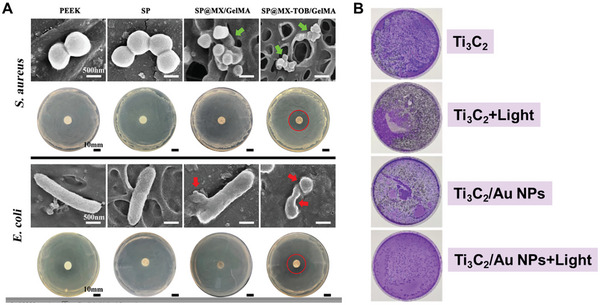
A) MXene‐based hydrogels with embedded antibiotics for prevention of bacterial infection. B) SEM images of bacteria on different samples for 1 day and the inhibition zones of the substrates. (A,B) Reproduced with permission.^[^ ^223^ ^]^ Copyright 2020, American Chemical Society.

Previous studies showed that the inclusion of 2D materials such as MXene into hydrogels can be beneficial for antibiotic delivery, due to the enhanced drug loading capacity, increased stability, and sustained release profiles.^[^ ^330^ ^]^ Therefore, various forms of drugs and antibiotics can be incorporated into such hydrogels to be delivered to injured and infected tissues. Providing localized release of such drugs can avoid off‐target delivery and prevent any potential side effects.^[^ ^326^ ^]^ Yin et al. developed MXene‐GelMA hydrogels loaded with tobramycin (TOB) to prevent bacterial infection and promote osteogenicity. The antibacterial effect of hydrogels was evaluated toward *S. aureus* and *E. coli*. The hydrogels showed a significant destructive effect toward *S. aureus* which lasted more than 24 h. Such antibacterial performance was attributed to the role of Ti_3_C_2_ nanosheets and released TOB, which enhanced the germicidal properties (**Figures** 21H and **23**).^[^ ^223^ ^]^ In another study, when lysozyme was loaded into MXene‐polydopamine (PDA), high antibacterial performance (>95%) against *S. aureus* was achieved, which was used for wound disinfection.^[^ ^331^ ^]^ Similarly, the addition of silver nanoparticles (AgNPs) into MXene‐based hydrogels provided them with superior antibacterial properties which can be used for infectious wounds.^[^ ^332^ ^]^ Apart from MXene, graphene nanosheets have also demonstrated potential for these applications. In a recent study, polyetheretherketone (PEEK)‐graphene composite was coated with hydroxyapatite (HA)‐based drug‐laden for bone defect repair and osteomyelitis. *S. aureus* and *E. coli*. were used to evaluate the antibacterial properties, and results indicated the material eradicated nearly a total of bacterial strains.^[^ ^333^ ^]^ He et al. employed BP nanosheets combined with CS to address post‐operation infections and osteosarcoma management. The presence of BP nanosheets enabled bactericidal ROS generation for infection control, while CS was used as a drug release platform. It was shown that *E. coli* and *S. aureus* were effectively eradicated. The bactericidal properties of the CS were attributed to the hydrophobic interactions, as well as the electrostatic interaction between the chitosan and bacteria cell wall.^[^ ^334^ ^]^

Another effective strategy for bone infection therapeutics is macrophage phenotype regulation by hydrogels. It is known that macrophages undergoing a shift to the M1 phenotype release an extensive quantity of cytokines that promote inflammation; while cells transitioning to the M2 phenotype can produce cytokines that have anti‐inflammatory effects and can inhibit the inflammatory response.^[^ ^335^ ^]^ Fu et al. demonstrated the macrophage phenotype regulation by using alginate/graphene oxide/sericin/nanohydroxyapatite (Alg/GO/Ser/nHAP) nanocomposite hydrogels. It was shown that sericin can induce macrophage phenotype transformation, and thus can inhibit inflammatory response, which would improve the immune microenvironment of the bone defects.^[^ ^336^ ^]^ GO‐loaded processed pyritum (PP) was used in another study for the design of composite hydrogels. Analysis of immune factors showed that the hydrogels loaded with PP/GO could effectively induce macrophage differentiations into the M2‐type (reduced local inflammation); while bone regeneration was also promoted.^[^ ^337^ ^]^ Using the same strategy, Zou et al. loaded interleukin‐4 (IL‐4) and bone morphogenetic protein‐2 (BMP‐2) into GO for the development of composite hydrogel. Their findings suggested that the introduction of this hydrogel significantly diminished nearby inflammation, while simultaneously boosting bone regeneration after 8 weeks of implantation.^[^ ^338^ ^]^

### 6.2 Biofilm Disruption

Biofilms are formed by microbial infections and typically consist of bacteria and self‐secreted extracellular polymeric substances (EPS).^[^ ^339^ ^]^ They are resistant to frequently used antibiotics and thus can cause implant failure or even amputation.^[^ ^340^ ^]^ Therefore, developing anti‐biofilm materials and eradicating biofilms, especially for infected bone defects is of great importance for long‐term use.^[^ ^341^ ^]^

Previous studies have shown that the presence of 2D materials in hydrogels can enhance the hydrogel's functionality for biofilm disruption. For instance, GO can penetrate the biofilm and bacterial cell membrane, reaching the cytoplasm to enhance transmission effectiveness and eliminate the bacteria.^[^ ^342^ ^]^ Wu et al. developed GO‐based hydrogels loaded with Antisense DNA oligonucleotides (ASO) for biofilm management. The in vivo results showed that the biofilm formation was inhibited by decreasing the expression of YycFG with antisense; while the viability of *S. aureus* was modulated by GO. Such high antibiofilm performance and good biocompatibility of hydrogels make them ideal for minimally invasive infection management.^[^ ^343^ ^]^ BP nanosheets were utilized in another study combined with ZnO nanowires for the effective eradication of biofilms against implant‐associated infections. Benefiting from the presence of ZnO nanowires, a 99.5% eradication ratio of biofilm was achieved in vivo. Such superior performance was attributed to the antibacterial activities of ZnO nanowires, which can be triggered by NIR irradiation at a moderate temperature. Due to the enhanced sterilization ability, ZnO nanowires were able to permeate the biofilms and thoroughly kill the bacteria. This strategy can thus be employed in future medical devices for in situ antibiofilm adhesion and infection prevention.^[^ ^344^ ^]^ Another promising 2D material for biofilm disruption is MXene. Recent studies revealed that due to the high surface area of MXene, bacterial attachment is prevented, and a higher density of bactericides is obtained.^[^ ^345^ ^]^ When gold nanoparticles were incorporated into the aqueous solution of MXene, *S. aureus* was inhibited and killed upon photoactivation. Its biofilm production was also prevented in vitro. It was further demonstrated that the conversion of M1 macrophages to M2 macrophages can be facilitated, which showed promises for infected wounds’ healing.^[^ ^346^ ^]^

### 6.3 PTT

**Figure 24:**
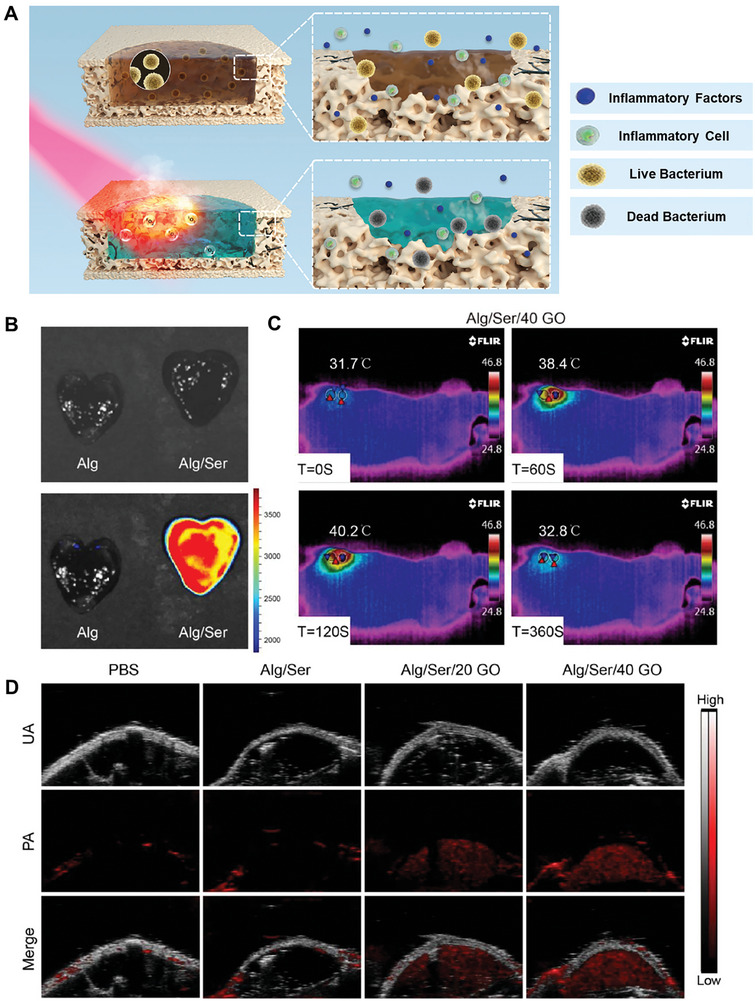
A) Illustration of the PTT for infection therapy and impaired bone repair. B) Fluorescence imaging of hydrogels, C) imaging of hydrogels using temperature variations caused by photothermal effect, D) photoacoustic imaging of hydrogels. (A) Reproduced with permission.^[^ ^349^ ^]^ Copyright 2022 Wiley‐VCH. (B–D) Reproduced with permission.^[^ ^352^ ^]^ Copyright 2021 Elsevier B.V. All rights reserved.

PTT has recently drawn great attention as a replacement for conventional antibiotics for infectious diseases and cancer therapeutics. The PTT mechanism relies on the absorption of laser energy by some agents known as photothermal agents and its conversion into heat under laser irradiation. 2D materials are in fact promising candidates to be used as photothermal conversion agents. This is due to their great photothermal effect under NIR irradiation. Furthermore, under laser irradiation, they are able to produce ROS, which is advantageous for inhibiting the growth of tumor cells and killing bacteria.^[^ ^223^, ^327^ ^]^ Such a mechanism is illustrated in **Figure** **24**A. Similar to PTT, microwave therapy which operates in the frequency range of 0–300 GHz has also been shown to be effective for non‐invasive treatment of osteomyelitis. For example, Jin et al. observed that 2D materials based on tubular carbon nanotubes (CNTs) combined with flake nanoflower‐shaped molybdenum disulfide (MoS_2_) can be effective for treatment through formation of heat and ROS under microwave irradiation.^[^ ^347^ ^]^

Due to the photothermal conversion efficiency and strong NIR absorption capacity of MXene, hydrogels based on MXene have been frequently reported for infection therapy and impaired bone repair. In a study conducted by Wu et al., PDA‐functionalized MXene nanosheets were used in alginate hydrogels. The photothermal antibacterial efficacy against both *S. aureus* and *E. coli* was evaluated in vitro. To do so, 808 nm NIR light was used to irradiate the hydrogels, and the real‐time thermographic images were captured. Results demonstrated that NIR irradiation can be used for controlling the photo‐responsive heating behavior of hydrogels. This was further used to regulate the M1/M2 macrophages for alleviating inflammation and acceleration of bone regeneration.^[^ ^348^ ^]^ When MXene was introduced into GelMA hydrogels, personalized composite hydrogels with excellent photothermal antibacterial and osteogenic abilities were obtained. The photothermal effect resulted in the destruction of microorganisms, which eventually reduced the inflammation of infected bones.^[^ ^14a^ ^]^ In a recent effort by Wang et al., CoFe_2_O_4_/MXene nanosheets hydrogel was developed for coating PEEK‐based implants. Light‐responsive antimicrobial efficiency of the materials was demonstrated (eliminating over 99% of bacteria), which supported the great potential of these hydrogels for infectious bone defect repairment.

Another promising 2D material for infected bone defect repair is BP. Jing et al. reported the fabrication of GelMA‐BP hydrogels. Magnesium ions were first bound to BP nanosheets through electrostatic attraction and then incorporated into hydrogels at different concentrations. Upon PTT, several inflammatory cells and bacteria experienced a noticeable drop, and the infection was controlled. Furthermore, due to the release of bioactive ions from the BP nanosheets, innerved bone regeneration was facilitated.^[^ ^349^ ^]^ BP nanosheets along with zinc sulfonate ligand (ZnL_2_) were further integrated into the hydroxyapatite (HA) scaffold. Results showed that at a mild photothermal temperature of less than 50 °C, over 99% of the bacteria can be eliminated. The bacterial death mechanism was believed to be due to the synergistic effects of ZnL_2_‐BPs with positive charges and the formation of ROS, which resulted in intracellular biomolecule destruction. Moreover, it was observed that continuous release of Zn^2+^ and PO_4_^3−^ facilitated osteogenesis.^[^ ^350^ ^]^ Eventually, the study performed by Li et al. suggested the importance of PDA in enhancing the photothermal efficiency of BP‐based hydrogels. At a wavelength of 808 nm, the drug release rate was monitored, and it was found that the addition of PDA not only controlled the drug release but also improved the efficiency of the carrier. The generated ROS further resulted in the elimination of bacterial infection.^[^ ^351^ ^]^

### 6.4 Imaging and Diagnosis

Due to the induced energy bandgaps and presence of defects, nano‐sized graphene and GO are photoluminescent which enable them to be used for diagnostics and biological imaging.^[^ ^353^ ^]^ Therefore, the incorporation of these materials into hydrogels can be used for multi‐modal imaging in orthopedic operations. Jiang et al. employed the same strategy via the inclusion of GO into alginate‐sericin hydrogels. To assess the photoluminescent property of the hydrogels, fluorescence microscopy was utilized. The alginate hydrogel was shown to be non‐photoluminescent, while alginate‐sericin hydrogel produced fluorescence upon excitation at 430 nm (Figure 24B). Given the photothermal property of GO, the changes in the temperature upon NIR irradiation can also be used for imaging and detecting the location of injected biomaterials in vivo as demonstrated in Figure 24C. Furthermore, based on the photoacoustic effect of NIR laser absorbers, photoacoustic imaging can also be used for non‐invasive imaging as shown in Figure 24D.^[^ ^352^ ^]^ He et al. successfully developed silk fibroin nanofiber‐GO hydrogels as a platform for tumor imaging. The up‐conversion fluorescence spectra were measured using a spectrophotometer (by excitation with a 980 nm laser), and the infrared camera was used for photothermal imaging. Results showed that the material can be used as an imaging agent and the signal was easily detected on the tumor. Furthermore, upon exposure to an 808 nm laser, infrared thermal images were obtained for the tumor‐bearing mice.^[^ ^354^ ^]^ The above studies have shown the significance of 2D materials, especially GO, for biomedical imaging applications. Nevertheless, this field is still unexplored and future studies are needed to explore the full potential of hydrogels based on 2D materials for targeted imaging, real‐time monitoring, multimodal imaging, and early disease detection.

**Table** **4** summarizes the studies on 2D materials incorporated composite hydrogels for bone infection management.

**Table 4:** Summary of hydrogels incorporated with 2D materials used for treatment of bone infections.

Strategy	Base Hydrogel Material	Incorporated 2D Material	Additive(s)	Function	References
Antibiotic/Drug Delivery	GelMA	MXene	SP, pDA, TOB	Significant killing effect on *S. aureus* which lasted more than 24 h	[223]
Antibiotic/Drug Delivery	HA	Graphene	STAC	Eradicated nearly total of bacterial strains	[333]
Antibiotic/Drug Delivery	Chitosan	BP	DOX	A near‐total eradication against *S. aureus* and *E. coli*	[334]
Antibiotic/Drug Delivery	Alginate	GO	Silk sericin, Nanohydroxyapatite	Regulated the transformation of macrophage phenotype and induced osteogenesis	[336]
Antibiotic/Drug Delivery	Poly(ethylene glycol) diacrylate (PEGDA) and carboxymethyl chitosan (CMC)	GO	Processed Pyritum (PP)	Macrophage differentiation into the M2‐type and promotion of bone regeneration	[337]
Antibiotic/Drug Delivery	poly(ethylene glycol) diacrylate (PEGDA) and carboxymethyl chitosan (CMC)	GO	IL‐4 and BMP‐2	Promoted both macrophage M2‐type differentiation and bone marrow mesenchymal stem cell osteogenesis differentiation	[338]
Biofilm Disruption	Alginate	GO	Antisense DNA Oligonucleotides (ASO)	Biofilm formation was inhibited, and the viability of *S. aureus* was modulated	[343]
Biofilm Disruption	PDA	BP	ZnO Nanowires	99.5% eradication ratio of biofilm was achieved in vivo	[344]
Biofilm Disruption	MXene	MXene	Au Nanoparticles	*S. aureus* was killed, and its biofilm formation was prevented	[346]
Photothermal Therapy	Alginate	MXene	PDA	Controlled the photo‐responsive heating behavior, regulated the macrophages for inflammation, bone regeneration	[348]
PTT	HA	Graphene	STAC	Photothermal Effect for killing osteosarcoma cells	[333]
Photothermal Therapy	GelMA	MXene	β‐TCP, Sodium alginate	Photothermal antibacterial and osteogenic abilities	[14a]
PTT	GelMA	BP	Magnesium Ions	Improved the inflammatory microenvironment, improved facilitated innerved bone regeneration	[349]
PTT	HA	BP)	ZnL2	Antibacterial photothermal treatment at a temperature less than 50 °C, osteogenesis	[350]
PTT	GelMA	BP	PDA	Controlled the drug release rate, enhanced photothermal efficiency	[351]
Imaging	Alginate	GO	Sericin	Fluorescence, photothermal, and photoacoustic imaging	[352]
Imaging	Silk fibroin nanofiber	GO	Lanthanide‐doped rare‐earth up‐conversion nanoparticles	Luminescence and photothermal imaging	[354]

## 7 Conclusion and Future Perspectives

In conclusion, as we demonstrated in this review, the integration of 2D materials like MXenes, graphene, GO, TMDCs, and BP into hydrogels holds immense potential for advancing bone regeneration and therapy for bone diseases. The unique properties of 2D materials, such as their high surface area, mechanical strength, tunable physicochemical, optical, and electrical characteristics, have enabled the development of composite hydrogels with enhanced biocompatibility, bioactivity, therapeutic, and bioimaging efficacy. Through synergistic interactions between 2D materials and hydrogel matrices, these composite systems have demonstrated promising outcomes in promoting osteogenesis, angiogenesis, and tissue integration, while also exhibiting antimicrobial and anti‐cancer properties, and controlled drug delivery capabilities, among others.

However, future research efforts should focus on addressing several key challenges and exploring new opportunities in the field of 2D materials‐based hydrogels for bone regeneration and disease therapy. First, there is a need for comprehensive understanding of the biocompatibility, degradation kinetics, and long‐term safety profile of these composite materials in vivo. Additionally, optimization of fabrication techniques and incorporation strategies to achieve precise control over material properties, such as mechanical strength, porosity, and degradation rate, will be crucial for tailoring hydrogel performance to specific clinical applications.

Furthermore, investigations of the underlying mechanisms governing the interactions between 2D materials released from hydrogel matrices and biological systems, including cellular signaling pathways, immune responses, and tissue remodeling processes, will provide valuable insights for the design of more effective therapeutic strategies. Moreover, exploring novel functionalities of 2D materials, such as their potential for photoresponsive or electrical stimulation‐based therapies, could open up new avenues for targeted, personalized, and minimally invasive treatments of bone‐related disorders.

Currently, the primary challenges hindering the clinical translation of 2D materials include the complexity of synthesis processes and the imperative for stability in material size and dispersion within aqueous solutions.^[^ ^355^ ^]^ Synthesizing 2D materials entails intricate procedures, and the capacity to scale up production while upholding quality and consistency remains crucial. Additionally, since many 2D materials contain exogenous substances recognized by the immune system, despite surface modifications, they may provoke abnormal thrombosis and vascular damage. This issue stands as a pivotal hurdle in the future application trajectory of these inorganic materials. Moreover, the establishment of regulatory guidelines for 2D materials in medical applications is imperative. Setting safety and efficacy standards for the utilization of 2D materials in bone regeneration and bone disease therapy is essential to facilitate their transition from laboratory research to clinical settings, necessitating stringent clinical trials and human evaluations. In general, collaborative interdisciplinary efforts between material scientists, bioengineers, clinicians, and regulatory agencies will be essential for translating research advancements in 2D materials‐incorporated hydrogels into clinically relevant solutions. By harnessing the unique properties of 2D materials and leveraging the versatility of hydrogel platforms, we can pave the way for the development of next‐generation therapies that address the unmet clinical needs in bone regeneration and disease management, ultimately improving patient treatment outcomes and their quality of life.

## Conflict of Interest

The authors declare no conflict of interest.
